# A single-cell, spatial transcriptomic atlas of the *Arabidopsis* life cycle

**DOI:** 10.1038/s41477-025-02072-z

**Published:** 2025-08-19

**Authors:** Travis A. Lee, Natanella Illouz-Eliaz, Tatsuya Nobori, Jiaying Xu, Bruce Jow, Joseph R. Nery, Joseph R. Ecker

**Affiliations:** 1 Plant Biology Laboratory, Salk Institute for Biological Studies, La Jolla, CA, USA.; 2 Genomic Analysis Laboratory, Salk Institute for Biological Studies, La Jolla, CA, USA.; 3 Howard Hughes Medical Institute, Salk Institute for Biological Studies, La Jolla, CA, USA.; 4 Present address: Sainsbury Laboratory, University of East Anglia, Norwich, UK.; 5 These authors contributed equally: Travis A. Lee, Natanella Illouz-Eliaz, Tatsuya Nobori.

## Abstract

*Arabidopsis* has been pivotal in uncovering fundamental principles of plant biology, yet a comprehensive, high-resolution understanding of its cellular identities throughout the entire life cycle remains incomplete. Here we present a single-nucleus and spatial transcriptomic atlas spanning ten developmental stages, encompassing over 400,000 nuclei from all organ systems and tissues—from seeds to developing siliques. Leveraging paired single-nucleus and spatial transcriptomic datasets, we annotate 75% of identified cell clusters, revealing striking molecular diversity in cell types and states across development. Our integrated approach identified conserved transcriptional signatures among recurrent cell types, organ-specific heterogeneity and previously uncharacterized cell-type-specific markers validated spatially. Moreover, we uncover dynamic transcriptional programs governing secondary metabolite production and differential growth patterns, exemplified by detailed spatial profiling of the compact yet complex apical hook structure; this profiling revealed transient cellular states linked to developmental progression and hormonal regulation, highlighting the hidden complexity underlying plant morphogenesis. Functional validation of genes uniquely expressed within specific cell contexts confirmed their essential developmental roles, underscoring the predictive power of our atlas. Collectively, this comprehensive resource provides an invaluable foundation for exploring cellular differentiation, environmental responses and genetic perturbations at high resolution, advancing our understanding of plant biology.

Multicellular organisms have evolved distinct organs to perform specific complex tasks and address challenges that may be present throughout their life. Unlike most animals, plants continuously undergo post-embryonic organogenesis to form new organs throughout development. In the model plant *Arabidopsis*, many tissues and organs present throughout its life cycle are composed of identical or similarly classified cell types that share functionality across organs and developmental stages^1^. For example, epidermal cells are the interface of the plant with the external environment for all organs, while internally localized vascular cells universally function in water and nutrient transport^2,3^. Despite the current understanding of plant anatomy, there is limited understanding of the molecular constituents that define cell types and states within the native context of diverse organs present throughout development.

While cell-type marker genes exist for many recurrent cell types across distinct organs, the form and function of cell types can vary depending on the tissue context or cellular niche from which they originate. Intra-cell-type heterogeneity is exemplified by *Arabidopsis* sepal and silique epidermal pavement cells, which undergo rapid cellular elongation depending on transcript dosages of the epidermal master regulator *ATML1* (ref. 4) or regulators of the cell cycle^5^ or in response to fertilization signals^6^, demonstrating that identically classified cell types can undergo varied developmental programs dependent on internal cues. In addition to cell-type classification, cellular states (which can be defined as variations in molecular phenotypes that do not impact the developmental potential^7^) may further differentiate individual cells within cell types and may reflect developmental factors, such as the cell cycle^8^, tissue/cellular age^9^, the spatial location and cellular neighbourhoods within tissues^10^, and external stimuli^11^. The molecular identity of a cell can thus be defined as the cumulative expression of transcripts that determine both cell type and state.

Single-cell RNA sequencing has been used to provide detailed maps of cell types in plants^12^. Still, its application is generally restricted to selected organs, tissues and cell types^13–17^ within individual studies^13–19^, posing a bottleneck towards a standardized comprehensive understanding of cell types and states in this model organism. While a recent study addresses this gap through the generation of single-nucleus transcriptomic datasets of tissues present throughout *Arabidopsis* development^20^, a challenge remains in the annotation and validation of large numbers of new marker genes, many of which may have no known function or are uncharacterized. To overcome this challenge, spatial transcriptomic methods present a powerful tool for the native detection of transcripts in situ within tissues, which simultaneously facilitates the validation of newly identified marker genes and the elucidation of spatially regulated expression^21–25^. Furthermore, the complementary application of single-cell and spatial transcriptomic technologies enables the investigation and elucidation of spatially and temporally regulated cellular states that potentially underlie biological function^26^ and environmental interaction^27^ within individual cell types. To date, however, there has not been wide application of single-cell and spatial omics to diverse *Arabidopsis* organs within individual studies. Here we present a single-nucleus transcriptome atlas with companion spatial transcriptome atlases as a foundational dataset of the *Arabidopsis* life cycle. These datasets reveal diverse molecular identities integrating cell type and state, including universal and development- and organ-specific cell-type marker genes.

The paired application of single-nucleus and spatial transcriptomic methods facilitated the identification of new cell-type-specific genes across diverse organs and enabled confident annotation of 138/183 (75%) of the clusters identified in our study. Furthermore, our paired single-nucleus and spatial transcriptomic datasets enabled the transcriptome-wide analysis of cellular states associated with rapid differential growth within the etiolated *Arabidopsis* seedling hypocotyl. Lastly, the examination of mutants in genes that we found to be under the combined regulation of cell-type and developmental specificity revealed functional roles of these transcripts in plants at the physiological level only within their native expression contexts. This study will be a powerful foundation for hypothesis generation that may facilitate discoveries within highly specific cell populations throughout the *Arabidopsis* life cycle.

## Results

### A spatiotemporally resolved atlas of the *Arabidopsis* life cycle

**Extended Data Fig. 1 |:**
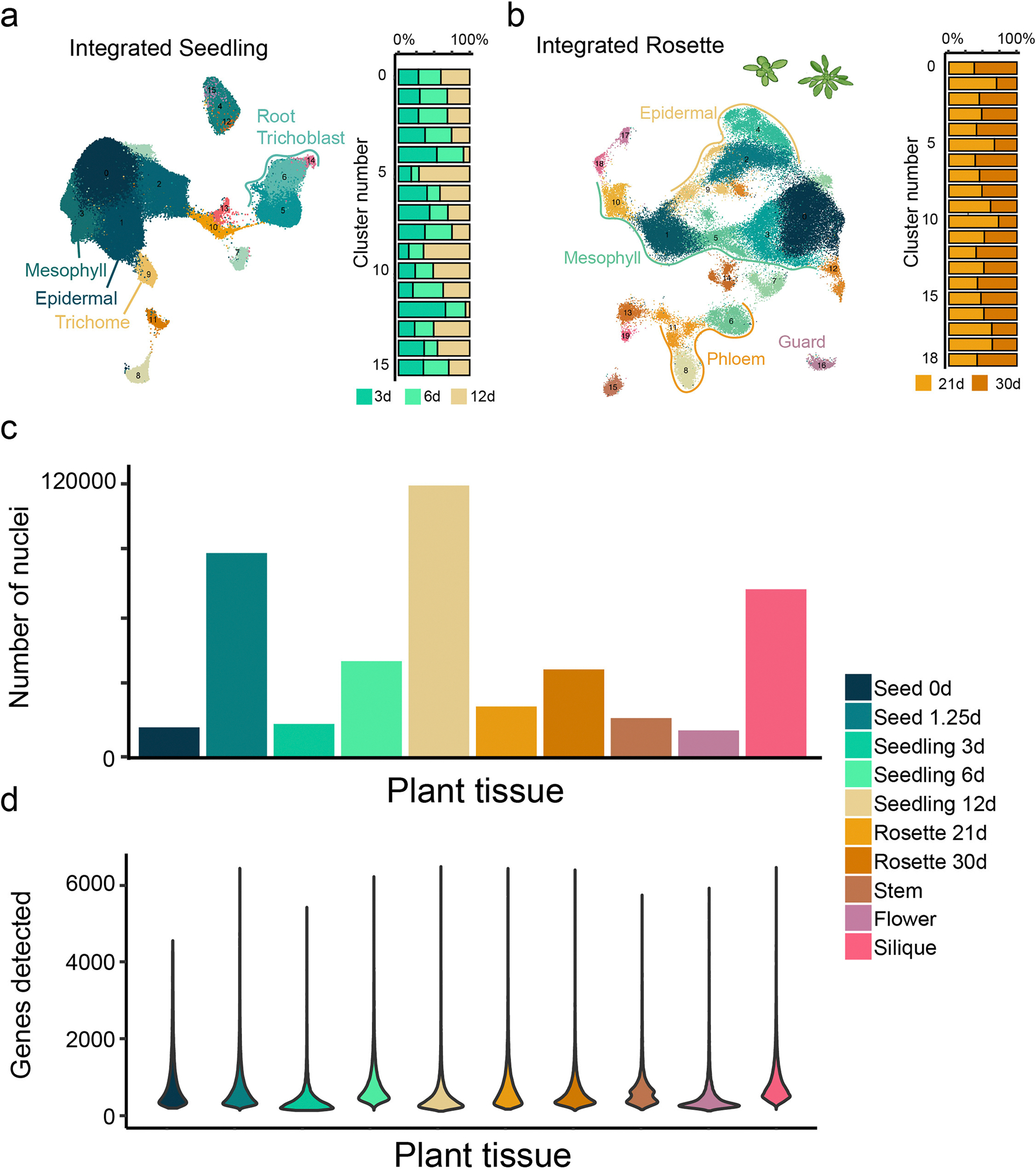
Overview and integration of single-nuclei datasets. **a**, Number of nuclei within each dataset. **b**, Number of genes detected per nucleus within each dataset. **c**, Integration of the 3d-, 6d-, and 12d-old seedling datasets. Barplot depicts the distribution of cells per dataset for each cluster. **d**, Integration of the 21d- and 30d-old rosette nuclei datasets. Barplot depicts the distribution of cells per dataset for each cluster.

**Fig. 1 |:**
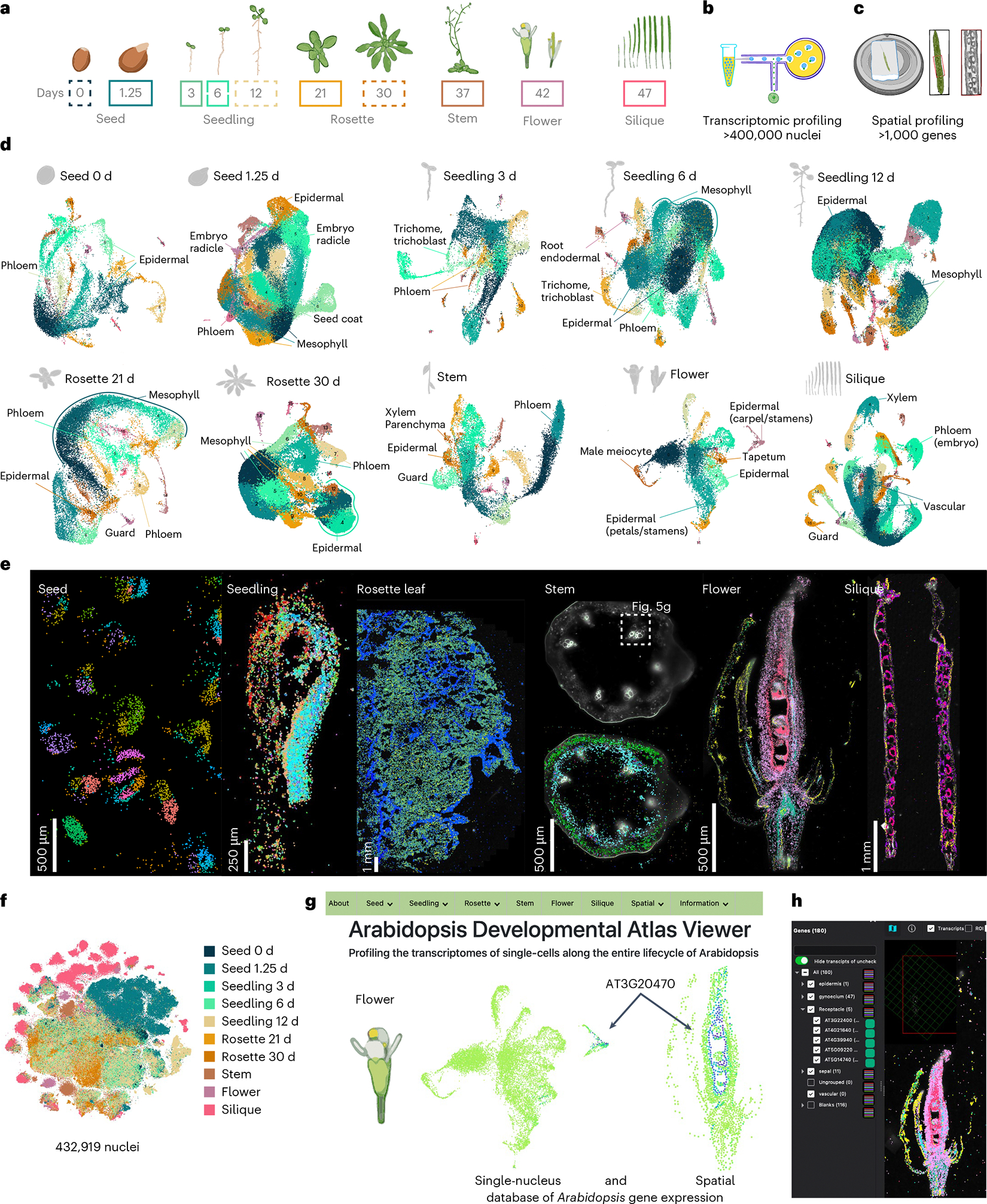
A spatially resolved transcriptional atlas of the *Arabidopsis* life cycle. **a**, The collected tissues over the ten developmental time points spanning the plant life cycle, including imbibed and germinating seeds (0 days and 1.25 days); three stages of seedling development (3 days, 6 days and 12 days); developing and fully expanded rosettes (21 days and 30 days); the stem (37 days), including basal, apical and branched regions; flower tissue (stages 6–15; ref. 30); and siliques (stages 2–10; ref. 29). Developmental time points surrounded by solid lines denote matching single-nucleus and spatial transcriptomic datasets. **b**,**c**, Paired single-nucleus and spatial transcriptomic datasets generated in our study. **d**, Uniform manifold approximation and projection (UMAP) plots of each dataset with select cluster annotations displayed for each dataset. **e**, Spatial transcriptomic assays analysed in this study, including *Arabidopsis* seed, seedling, mature leaf, stem, flower cross and longitudinal sections, and silique. The scale bar lengths are shown. **f**, *t*-distributed stochastic neighbour embeddings of the fully integrated dataset. Nuclei are coloured according to the tissue of origin. **g**, Web browser access to the Arabidopsis Developmental Atlas Browser and CELLxGENE instances of flower single-nucleus and spatial datasets. **h**, A spatial transcriptomic dataset within the MERSCOPE Visualizer software. For each micrograph in **e**, results were observed across ten independent seeds, eight independent seedlings, two independent leaves, one stem, three independent flowers (one in longitudinal orientation and two in cross orientation) and three independent siliques. See Methods for detailed information.

To generate a comprehensive atlas of *Arabidopsis* development, we collected six distinct organ systems that encompass diverse tissues as well as whole organisms at ten discrete developmental time points throughout the *Arabidopsis* life cycle corresponding to established developmental road maps^28–30^. This includes imbibed and germinating seeds, three stages of seedling development, developing and fully emerged rosettes, the stem, flowers and siliques (Fig. 1a). For each organ system, we also generated a paired spatial transcriptomic dataset (Fig. 1b–e; mature rosette leaf^27^). For the single-nucleus datasets, 432,919 nuclei from the ten developmental time points passed accepted droplet-based single-nucleus filtering metrics^31^ (median unique molecular identifier (UMI), 916; Extended Data Fig. 1 and Supplementary Fig. 1) and were independently clustered. For the seedling and rosette samples that constitute developmental time series, we also performed integrative analyses that revealed clusters corresponding to known cell types (Extended Data Fig. 1c,d), which confirms the reproducibility of these datasets and enables the investigation of developmentally regulated processes, such as root hair development (Supplementary Fig. 2) and leaf senescence (Supplementary Fig. 3), as reported recently^20^, across developmentally distinct samples. We also merged all single-nucleus RNA sequencing (snRNA-seq) datasets into a global dataset (Fig. 1f) encompassing all cell types across the *Arabidopsis* life cycle.

### Comprehensive identification of transcriptional identities

**Extended Data Fig. 2 |:**
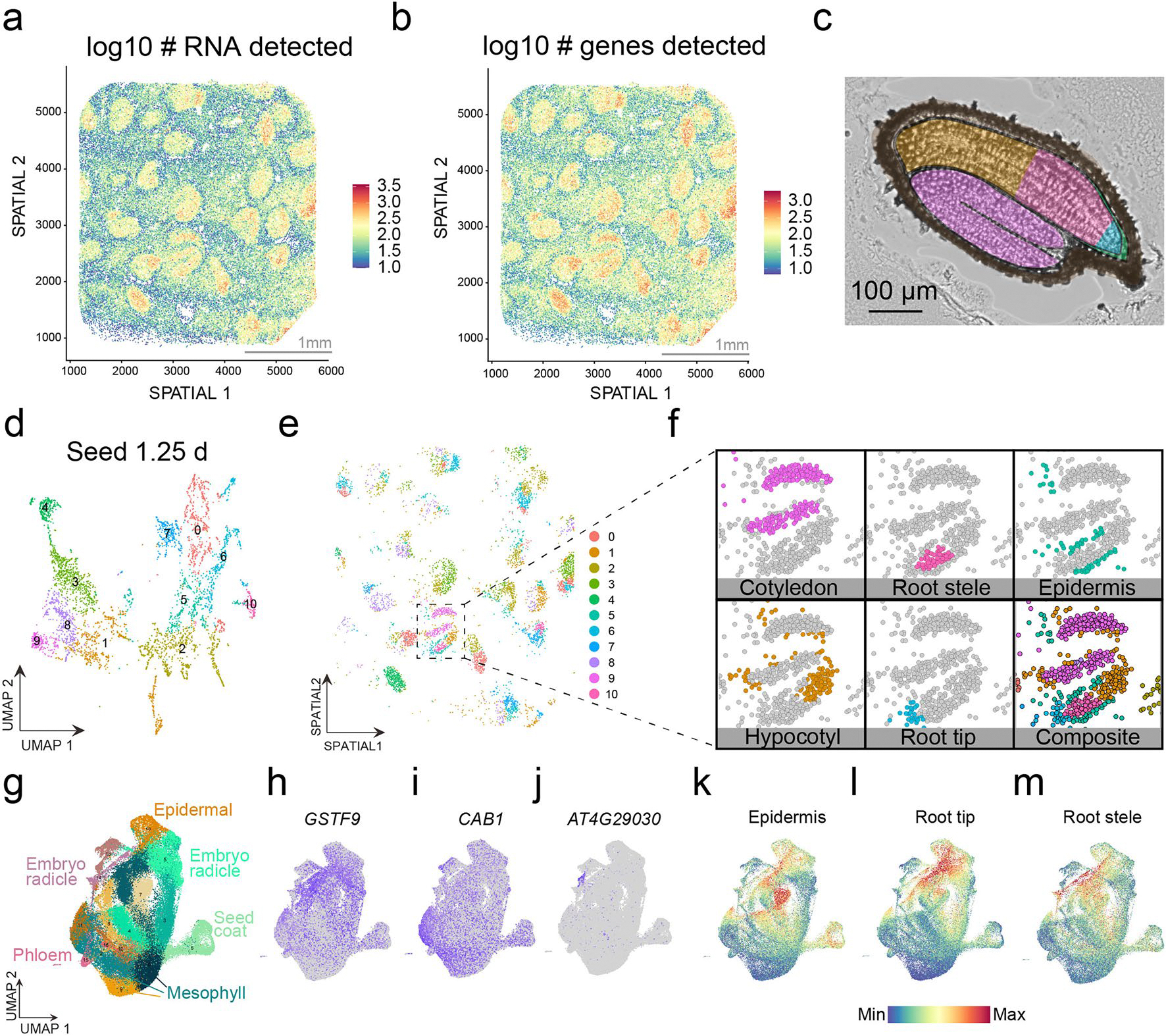
Spatially resolved cell type–specific expression in germinating *Arabidopsis* seeds. **a** and **b**, Quality metrics of the Curio spatial transcriptomics dataset of germinating seeds. Log10 values of (**a**) the number of transcripts and (**b**) the number of genes detected per spot in spatial coordinates. Scale bar length is depicted. **c**, Representative micrograph of a cryosectioned seed. Regions of the seedling that correspond to clusters in the spatial transcriptomic dataset are false colored accordingly. Scale bar length is depicted. **d**, Dimensionality reduction and *de novo* clustering of spatial transcriptomics data of 1.25-day-old germinating seeds. **e** and **f**, Mapping of clusters to spatial coordinates. Colors correspond to cell types and anatomical regions of germinating seeds (**c**). **g** to **m**, Cross-comparison of spatial transcriptomics derived cluster markers in the matched droplet-based single nucleus dataset. (**g**) Dimensionality reduction and clustering of the droplet-based 1.25 d seed dataset in Fig. 1d. (**h** to **j**) Expression of cluster markers identified in the spatial transcriptomics dataset corresponding to the (**h**) stele (cluster 4), (**i**) cotyledon (cluster 5), and (**j**) unannotated (cluster 6) in the droplet-based dataset. (**k** to **m**) Average expression of cluster markers identified in the spatial transcriptomics dataset corresponding to the (**k**) epidermis (cluster 10), (**l**) root tip (cluster 10), and (**m**) root stele (cluster 14) in the droplet-based dataset. For the micrograph depicted in c, results were observed in over ten seeds.

**Extended Data Fig. 3 |:**
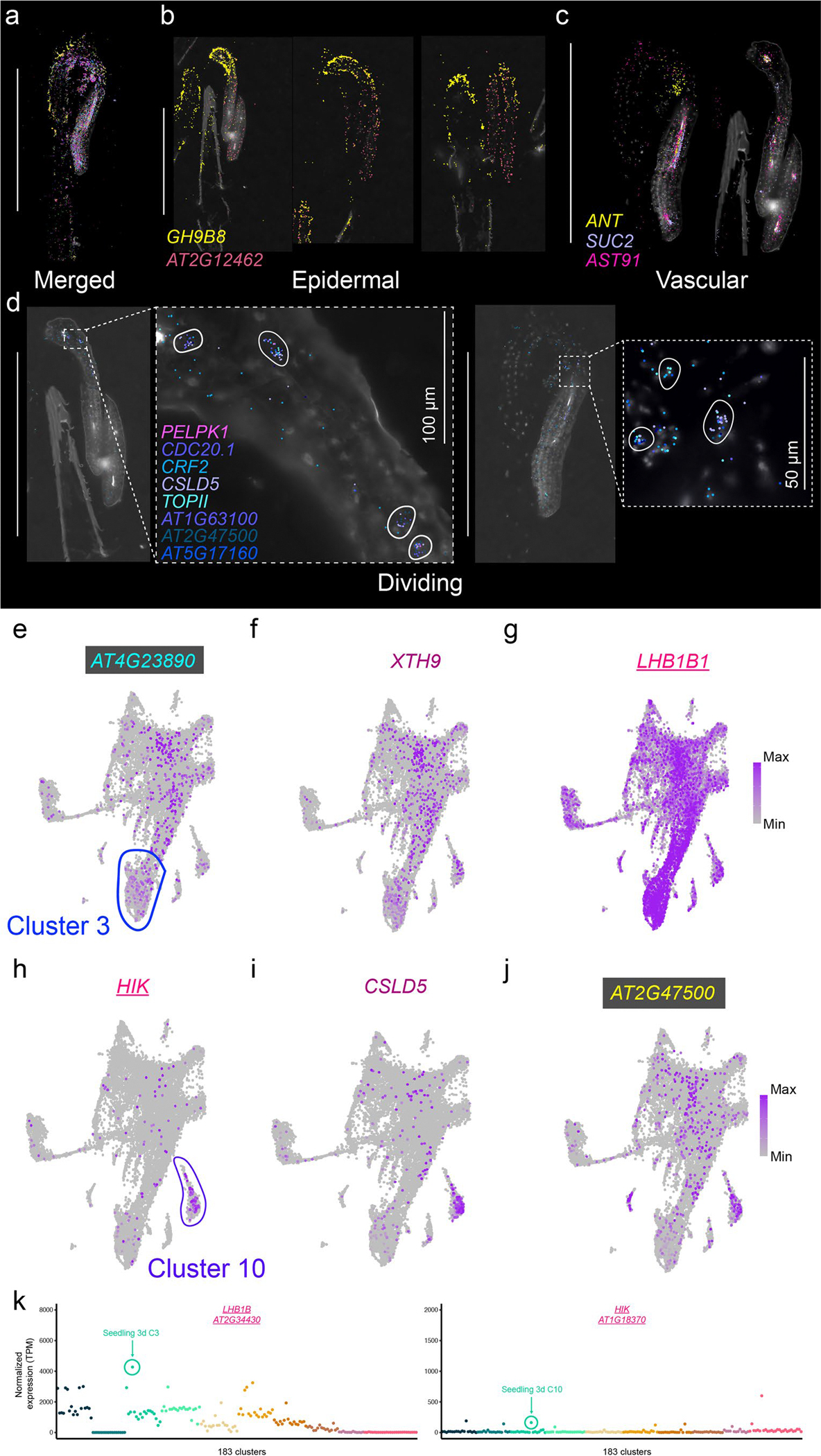
Spatially resolved cell identities in *Arabidopsis* seedlings. **a**, Merged image showing the spatial distribution of transcripts representing distinct cell types and regions in 3-day-old *Arabidopsis* seedlings. **b**, Spatial localization of transcripts for GLYCOSYL HYDROLASE 9B8 (*GH9B8)* and *AT2G12462*, marking epidermal cells in the hypocotyl and cotyledons, respectively. **c**, Spatial distribution of transcripts for AINTEGUMENTA (*ANT)*, *SUC2*, and *AST91*, representing vascular cell identities. **d**, Spatial expression of transcripts associated with dividing cells in cluster 10 of the 3-day-old seedling dataset. Genes shown include *PELPK1*, *CDC20.1*, *CRF2*, *CSLD5*, *TOPII*, *AT1G63100*, *AT2G47500*, and *AT5G17160*. Insets highlight cells with high transcript abundance of these division-associated genes. **e**-**j**, Expression of select spatial markers displayed in (**a**)-(**d**) in the 3-day-old seedling single nucleus dataset. (**e**), *AT4G23890* is specifically enriched in cluster 3 (outlined in blue), associated with mesophyll identity. (**f**), *XTH9*, a cell wall remodeling gene, shows broad expression with enrichment in the same region as *AT4G23890*. (**g**), *LHB1B1*, a light-harvesting complex gene, is enriched in cluster 3 cells but is also highly expressed across a wide range of cells, indicating widespread photosynthetic activity. (**h**), *HIK*, required for cytokinesis and is involved in physical cell division, is enriched in cluster 10 (outlined in purple), corresponding to dividing cells. (**i**), *CSLD5*, a cellulose synthase-like gene involved in cell plate formation during cytokinesis, displays punctate expression across dividing populations. (**j**), *AT2G47500* shows broad expression with enrichment in dividing clusters. Color intensity represents normalized expression levels (purple gradient, min to max). Scale bars: 1 mm ([**a**]–[**c**], left panel in **d**); 100 μm (left inset in **d**); 50 μm (right inset in **d**). **k**, Normalized pseudobulk expression of spatial transcriptomic markers depicted in Fig. 2 across 183 clusters. Points are colored by dataset. The cluster with the highest expression of the spatial marker within the matched 3d-old-seedling dataset is indicated. For the micrographs depicted in (**a**)-(**d**), results were observed in eight seedlings.

**Extended Data Fig. 4 |:**
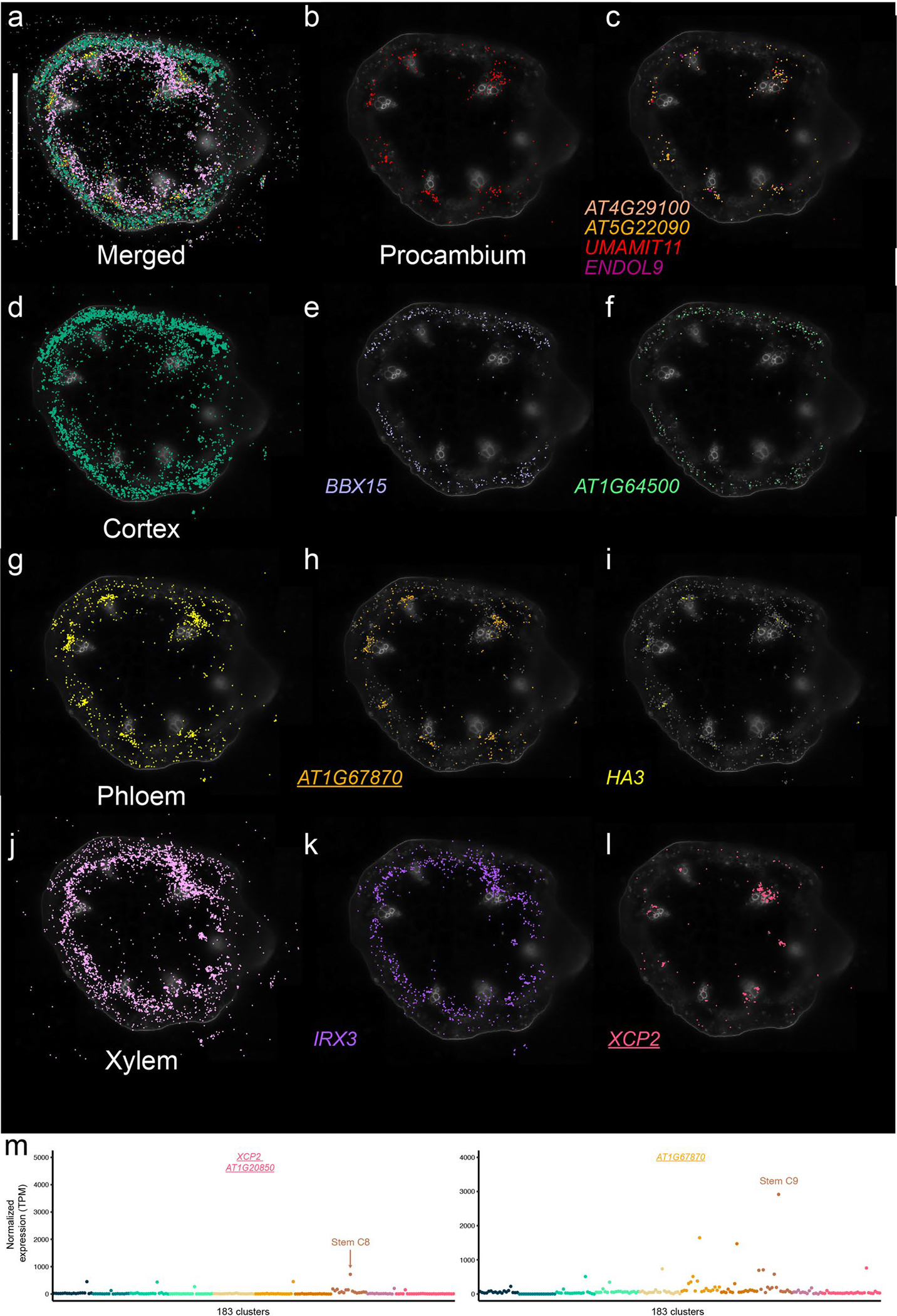
Spatially resolved cell type–specific expression in the *Arabidopsis* stem. **a**, Merged image showing the spatial distribution of transcripts representing distinct cell types and regions in the *Arabidopsis* stem. **b**, Merged image of transcripts enriched in procambium cells. **c**, Spatial expression of *AT4G29100*, *AT5G22090*, *UMAMIT11*, and *ENDOL9* in procambium regions. **d**, Merged image of transcripts localized to cortex cells in the stem. **e**-**f**, Spatial distribution of (**e**) *BBX15 and ( f) AT1G64500* transcripts in cortex cells. **g**, Merged image of transcripts localized to phloem cells in the stem. **h**-**i**, Spatial distribution of (**h**) *AT1G67870* and (**i**) *HA3* transcripts in phloem cells. **j**, Merged image of transcripts localized to xylem cells in the stem. **k**-**l**, Spatial distribution of (**k**) *IRX3* and (**l**) *XCP2* transcripts in xylem cells. Scale bar = 1 mm. **m**, Normalized pseudobulk expression of spatial transcriptomic markers depicted in (**h**) and (**l**), across 183 clusters. Points are colored by dataset. The cluster with the highest expression of the spatial marker within the matched stem dataset is indicated. For the micrographs depicted in **a**-**l**, results were observed in one to one stem.

**Extended Data Fig. 5 |:**
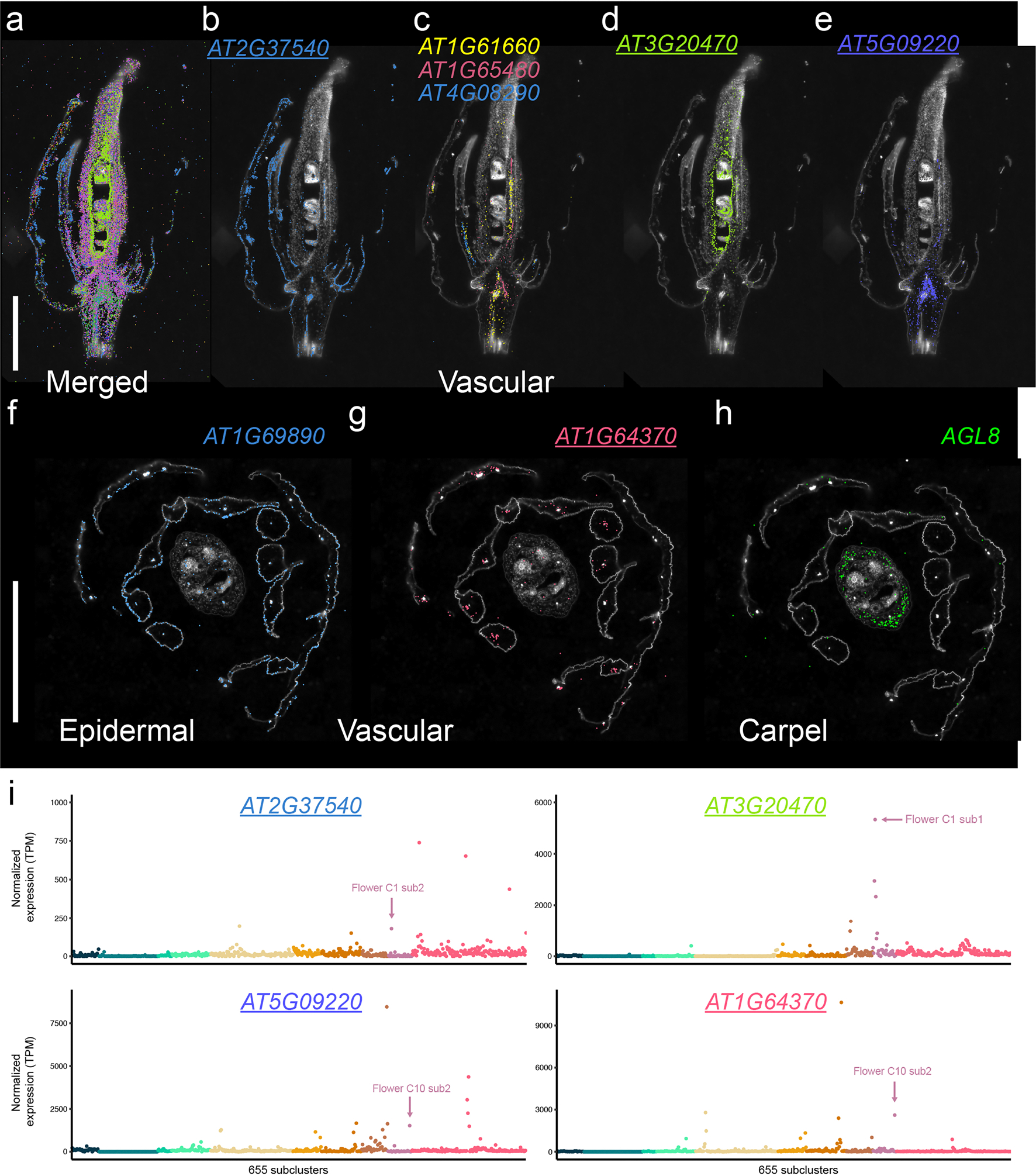
Spatially resolved cell identities in the *Arabidopsis* flower. **a**, Merged image showing transcripts expressed in distinct cell types and regions of the flower. **b**, Spatial distribution of *AT2G37540* transcripts marking epidermal cells in the outer layers of sepals and petals. **c**, Spatial distribution of *AT1G61660*, *AT1G65480 - FLOWERING LOCUS T* (*FT*), and *AT4G08290* transcripts identifying vascular tissues within the flower. **d**, Localized expression of *AT3G20470* in vascular regions. **e**, Localized expression of *AT5G09220* in the lower portion of the flower. **f**, Expression of *AT1G69890* in epidermal tissues in a cross-section of the flower. **g**, Expression of *AT1G64370* in vascular cells within a floral cross-section. **h**, Expression of *AGAMOUS-LIKE 8* (*AGL8*) / *FRUITFULL* (*FUL*) in the carpel region of the flower. Scale bar = 1 mm. **i**, Normalized pseudobulk expression of spatial transcriptomic markers depicted in (**b**), (**d**), (**e**), and (**g**), across 655 subclusters. Points are colored by dataset. The subcluster with the highest expression of the spatial marker within the matched flower dataset is indicated. For the micrographs depicted in **a**-**e**, results were observed in one longitudinally sectioned flower. For the micrographs depicted in **f**-**h**, results were observed in two cross-sectioned flowers.

**Extended Data Fig. 6 |:**
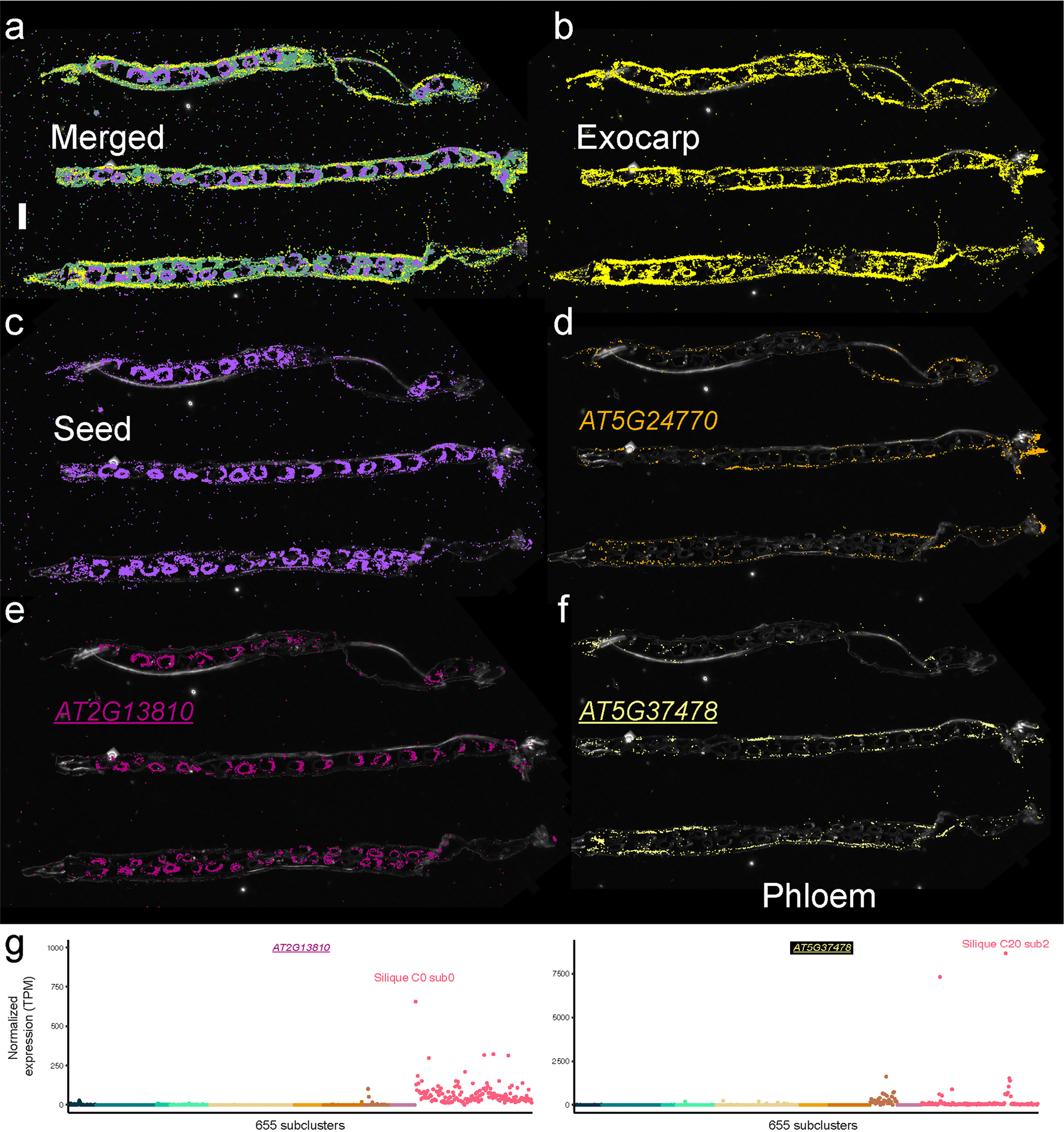
Spatially resolved cell identities in the *Arabidopsis* silique. **a**, Merged image displaying transcripts expressed in distinct cell types and regions of the Arabidopsis silique. **b**, Merged image highlighting transcripts localized to the exocarp cells. **c**, Merged image showing transcripts localized to the developing seeds within the silique. **d**, Spatial distribution of *AT5G24770* - VEGETATIVE STORAGE PROTEIN 2 (*VSP2*) transcripts in the exocarp cells. **e**, Spatial distribution of *AT2G13810* transcripts in the developing seeds. **f**, Spatial distribution of *AT5G37478* (TARGETING PROTEIN FOR XKLP2 protein family [*TPX2*]) transcripts in the exocarp cells. Scale bar = 1 mm. **g**, Normalized pseudobulk expression of spatial transcriptomic markers depicted in (**e**), and (**f**) across 655 subclusters. Points are colored by dataset. The subcluster with the highest expression of the spatial marker within the matched silique dataset is indicated. For the micrographs depicted in **a**-**f**, results were observed in three siliques.

**Fig. 2 |:**
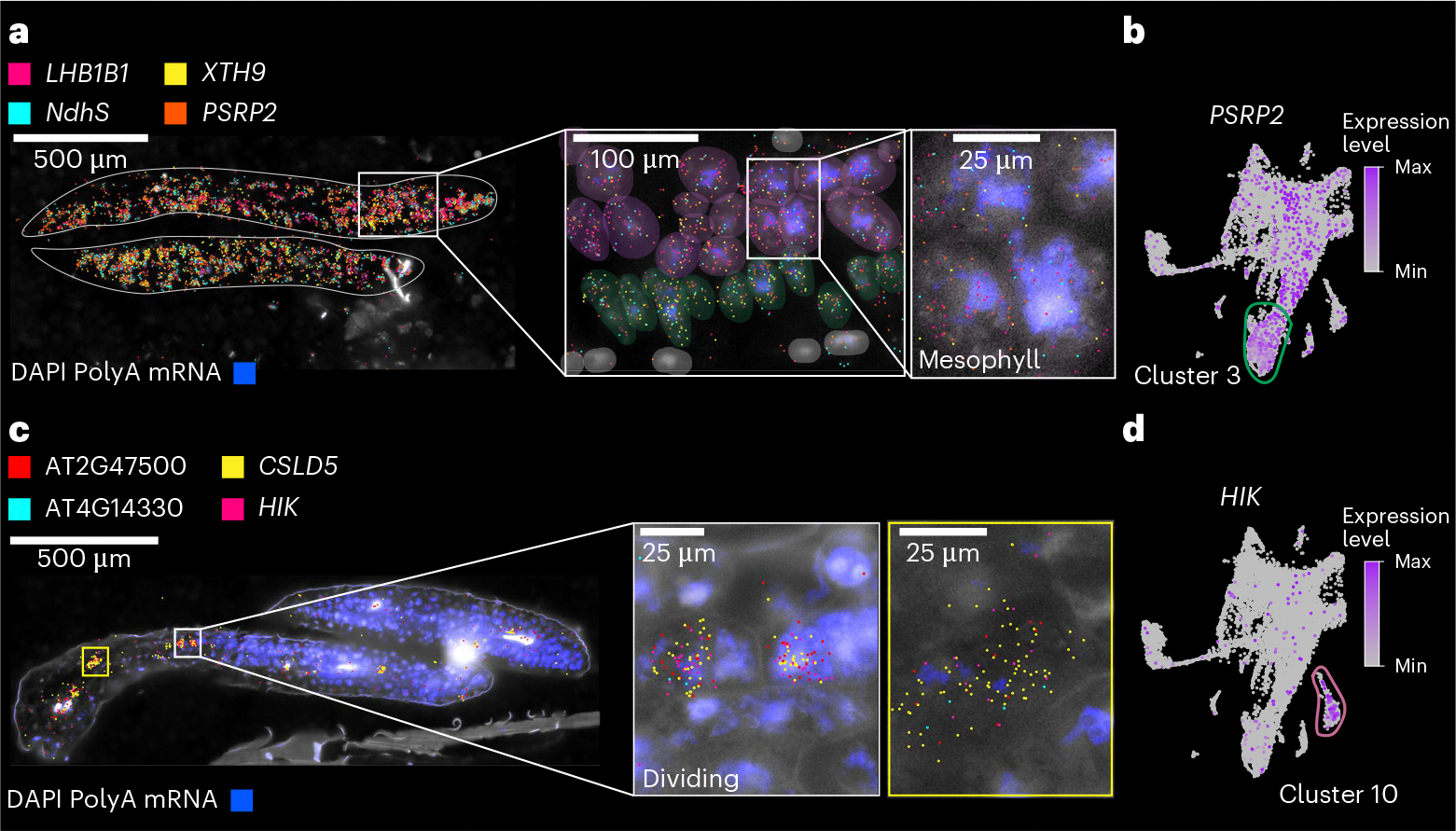
Spatial validation of snRNA-seq data in *Arabidopsis* tissues/seedlings. **a**, Spatial transcriptomic visualization of selected gene expression (*LHB1B1* (AT2G34430), *XYLOGLUCAN ENDOTRANSGLUCOSYLASE/HYDROLASE 9* (*XTH9*; AT4G03210), *NAD(P)H dehydrogenase subunit S* (*NdhS*; AT4G23890) and *PLASTID-SPECIFIC RIBOSOMAL PROTEIN 2* (*PSRP2*; AT3G52150)) in the *Arabidopsis* seedling (zoomed in on the cotyledons). The higher-magnification images highlight the localization of transcripts within mesophyll cells. DAPI staining (blue) indicates nuclei. The scale bar lengths are shown. **b**, UMAP plot of single-nucleus transcriptomes showing expression of *PSRP2* (purple dots), enriched in cluster 3 (outlined in green). **c**, Spatial mapping of cluster 10 annotated as ‘dividing cells’ in the three-day-old seedling, with transcripts from known and new cell-cycle- and division-related genes (AT2G47500, *CSLD5* (AT1G02730), AT4G14330 and *HIK* (AT1G18370), marked in magenta, yellow, cyan and pink, respectively). The insets show individual dividing cells with colocalized transcripts. DAPI staining (blue) indicates nuclei. The scale bar lengths are shown. **d**, UMAP plot highlighting expression of *HIK* enriched in cluster 10 (outlined in purple), corresponding to dividing cells in the tissue. For the micrographs in **a** and **c**, results were observed in eight seedlings. DAPI, 4′,6-diamidino-2-phenylindole.

To compare cell types across organs and development, we analysed each dataset independently, which resulted in 183 clusters across all datasets (Fig. 1d). Cluster annotation was performed according to the following guidelines. First, an extensive list of marker genes was compiled with known cell-type- and tissue-specific expression in all *Arabidopsis* organs (Supplementary Table 1), including cell-type-specific markers recently identified from single-cell RNA-seq studies^18,19,32–35^. Many of these known cell-type-/tissue-specific expressed genes were enriched in specific clusters of our dataset (>1,000 genes), which aided cluster annotation (Supplementary Fig. 4a). Second, a cell-type enrichment score for each cluster was calculated on the basis of known cell-type markers to systematically infer cell types (Methods and Supplementary Fig. 4b). Third, we investigated newly identified cluster markers within each dataset using previously generated dissection-based and cell-type-specific transcriptomic studies (TAIR^36^ and ePlant^37,38^) to confirm the accuracy of our cluster annotations (Supplementary Table 2). Finally, we spatially validated a selection of cluster markers for each tissue/organ using sequencing- and imaging-based spatial transcriptomic technologies (Fig. 2 and Extended Data Figs. 2–6).

Our spatial transcriptome approach allowed us to validate examples of known cell-type marker genes across organs and development, as well as validate newly identified cell-type-specific and tissue-specific marker genes across all organs (see Supplementary Table 3 for 109 examples of new cell-type/tissue marker genes). In contrast, we were also able to identify and validate markers that do not universally specify cell types but rather demonstrate cell-type-specific expression only within the context of specific organs, as shown for epidermal cells of seedling hypocotyls (Extended Data Fig. 3b). Spatial transcriptomics confirmed the localization of cell-type-specific genes identified via snRNA-seq in most developmental stages and plant organs tested (Extended Data Figs. 2–6). In the seedling datasets, we were able to confirm cell identities such as mesophyll cells expressing *LIGHT-HARVESTING CHLOROPHYLL-PROTEIN COMPLEX II SUBUNIT B1* (*LHB1B1*; AT2G34430)^38^, epidermal cells (for example, *GLYCOSYL HYDROLASE 9B8* (*GH9B8*; AT2G32990) and AT2G12462, new epidermal markers restricted to the apical hook and cotyledons in seedlings, respectively) and vascular cells (*AINTEGUMENTA* (*ANT*; AT4G37750), which was shown to regulate *ERECTA-LIKE 1* (*ERL1*; AT5G62230) and *PHLOEM INTERCALATED WITH XYLEM* (*PXY*; AT5G61480), which maintain procambial cell identity^39^) that are co-expressed in the same cluster as *SUCROSE-PROTON SYMPORTER 2* (*SUC2*; AT1G22710)^40–42^ (Fig. 2 and Extended Data Fig. 3). We were also able to annotate clusters to cell states through the cluster-specific expression of cell-division-associated genes such as *CELLULOSE SYNTHASE LIKE-D* (*CSLD5*; AT1G02730), involved in cell plate formation^43^, and *HINKEL* (*HIK*; AT1G18370), which is involved in cytokinesis^44^ (Fig. 2 and Extended Data Fig. 3).

Visual inspection of spatial marker expression within the single-nucleus datasets did not always reveal cluster-specific expression patterns, as demonstrated by *LHB1B1* (Extended Data Fig. 3g), but quantitative evaluation of these markers revealed cluster- and subcluster-specific and enriched expression of these spatial markers both within individual samples (Extended Data Fig. 3k, left) and across tissues and developmental time points (Extended Data Fig. 3k, right), demonstrating conserved expression of these cell-type and cell-state markers across diverse tissues throughout the *Arabidopsis* life cycle.

In stems, we identified cell-type-specific gene expression localized to the procambium (for example, *USUALLY MULTIPLE AMINO ACIDS MOVE IN AND OUT TRANSPORTERS 11* (*UMAMIT11*; AT2G40900)), cortex (for example, *B-BOX DOMAIN PROTEIN 15* (*BBX15*; AT1G25440)), phloem (*H(+)-ATPase 3* (refs. 45,46) (*HA3*; AT5G57350)) and xylem tissues (*XYLEM CYSTEINE PEPTIDASE 2* (*XCP2*; AT1G20850) and *IRREGULAR XYLEM 3* (ref. 47) (*IRX3*; AT5G17420)), enabling high-resolution mapping of vascular differentiation in the stem (Extended Data Fig. 4). Spatial expression of markers in *Arabidopsis* floral tissue revealed epidermal markers (for example, AT2G37540), vascular-expressed genes (for example, *FLOWERING LOCUS T* (*FT*; AT1G65480)) and carpel-specific expression of *AGAMOUS-like 8* / *FRUITFULL* (AT5G60910; ref. 48) (Extended Data Fig. 5). Spatial transcriptomics also revealed distinct expression domains within the *Arabidopsis* silique, with *VEGETATIVE STORAGE PROTEIN 2* (*VSP2*; AT5G24770) and the TPX2 protein family (*TPXL6*; AT5G37478) marking the exocarp cell layer, and AT2G13810 showing specific localization in developing seeds (Extended Data Fig. 6). Together, these examples demonstrate the strength of our paired single-nucleus and spatial transcriptomic datasets on matched biological samples.

While we were able to annotate many clusters to individual cell types, we found that some clusters instead corresponded to anatomical regions or broad cellular states, such as the developing embryo or dividing cells (Supplementary Table 2). This led us to hypothesize that further transcriptional complexity could be resolved within each cluster when examined independently. Through systematic re-clustering of individual clusters (subclustering), we were able to determine further complexity within clusters, resulting in a total of 655 subclusters (Supplementary Fig. 5a). Correlating the aggregated transcriptomes (pseudobulk) of each subcluster, we found that subclusters generally grouped by sample type, but we also found examples of subclusters with unique gene expression patterns that grouped independent of dataset (Supplementary Fig. 5b). Together, these results demonstrate that subclustering captures subtle transcriptional states and uncovers rare or transitional cell populations that may play distinct roles across developmental or environmental contexts.

### Spatially resolved cell layer annotation

**Fig. 3 |:**
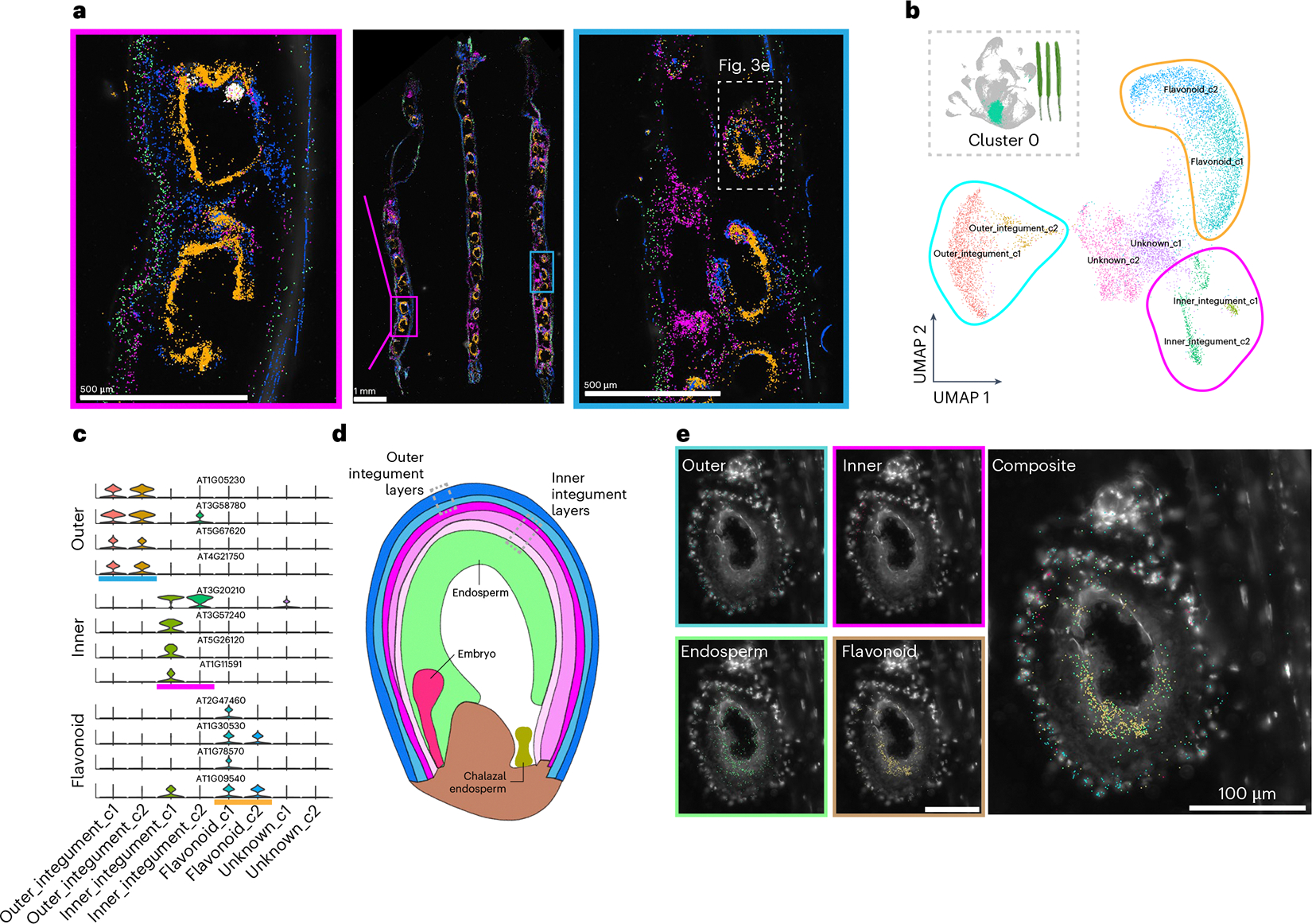
Spatial annotation of individual cell layers in seed sacs. **a**, Visualization of all 43 transcripts targeted with MERFISH in siliques. Groups of transcripts are false-coloured according to cell type and region-specific expression. Magnified images of individual seed sacs are also depicted. The scale bar lengths are shown. **b**,**c**, Subclustering of cluster 0 from the silique dataset. Groups of clusters corresponding to the outer integument (blue), inner integument (pink) and flavonoid biosynthesis (orange) clusters are depicted (**b**), with violin plots of select cluster markers (**c**). **d**, Relevant cell types and structures of developing seed sacs. **e**, Magnified images of an individual seed sac in the spatial dataset. Subcluster markers are coloured corresponding to the annotated cluster grouping. The scale bar length is shown. For the micrographs in **a** and **e**, results were observed in three siliques and at least ten seed sacs within the siliques.

The silique represents a complex system that includes distinct maternal and progeny tissues. We therefore used the silique dataset as a use case example of dataset annotation that is enhanced by the paired single-nucleus and spatial transcriptomic datasets (Fig. 3a). Within the proliferating cell population (cluster 0) that may encompass multiple cell types in siliques, subclustering revealed three transcriptionally distinct groups, with each defined by unique marker genes (Fig. 3b,c). Expression of these markers was restricted to the seed coat layer^49^, prompting further spatial investigation.

By mapping these markers in the spatial dataset, we were able to finely annotate subcluster groups corresponding to the seed coat’s outer and inner integument layers (Fig. 3a,d,e). This annotation of individual layers within developing seeds was further supported by a similar annotation of these genes in a single-nucleus transcriptome dataset of *Arabidopsis* seeds^35^. Consistent with a previous single-nucleus dataset of developing seeds^35^, we also identified subcluster markers corresponding to flavonoid biosynthetic processes within our subcluster groups (Fig. 3b,c and Supplementary Fig. 6), but spatial mapping revealed that these transcripts are detected in both the seed coat and endosperm layers of seed sacs (Fig. 3e, beige). While flavonoid secondary metabolites are known to function in seed coat colour and cause the transparent testa phenotype in mutants of flavonoid biosynthetic enzymes^50,51^, the spatial expression of *TRANSPARENT TESTA 4* (*TT4*; AT5G13930) and other flavonoid biosynthesis genes within the endosperm layer may instead be associated with the alteration of fatty acid levels in embryos. This has been observed in *tt2* (AT5G35550)^52^, *tt4* (AT5G13930)^53^ and *tt8* (AT4G09820)^54^ mutants. The simultaneous but spatially distinct expression of genes encoding flavonoid biosynthesis enzymes may suggest functionally diverse roles of flavonoid metabolites within discrete cell types of individual seeds. Together, these findings demonstrate that transcriptional states may be strongly associated with secondary metabolite production and inclusive of multiple cell types, further demonstrating that the paired application of single-nucleus and spatial transcriptome datasets enables the discovery and annotation of marker genes at the resolution of individual cell layers.

### Elucidation of polarity defines cellular states

Spatially confined expression of polarity regulators is a hallmark of de novo organogenesis in various *Arabidopsis* organs^10,55,56^. We hypothesized that our *Arabidopsis* life-cycle atlas would capture polarity-defined cells. In our single-nucleus datasets, we detected transcripts of canonical polarity markers across several clusters (Supplementary Fig. 7), suggesting the presence of such cells within our datasets. However, these transcripts were sparsely expressed, limiting our ability to fully resolve polarity domains on the basis of single-nucleus data alone (Supplementary Fig. 7).

**Fig. 4 |:**
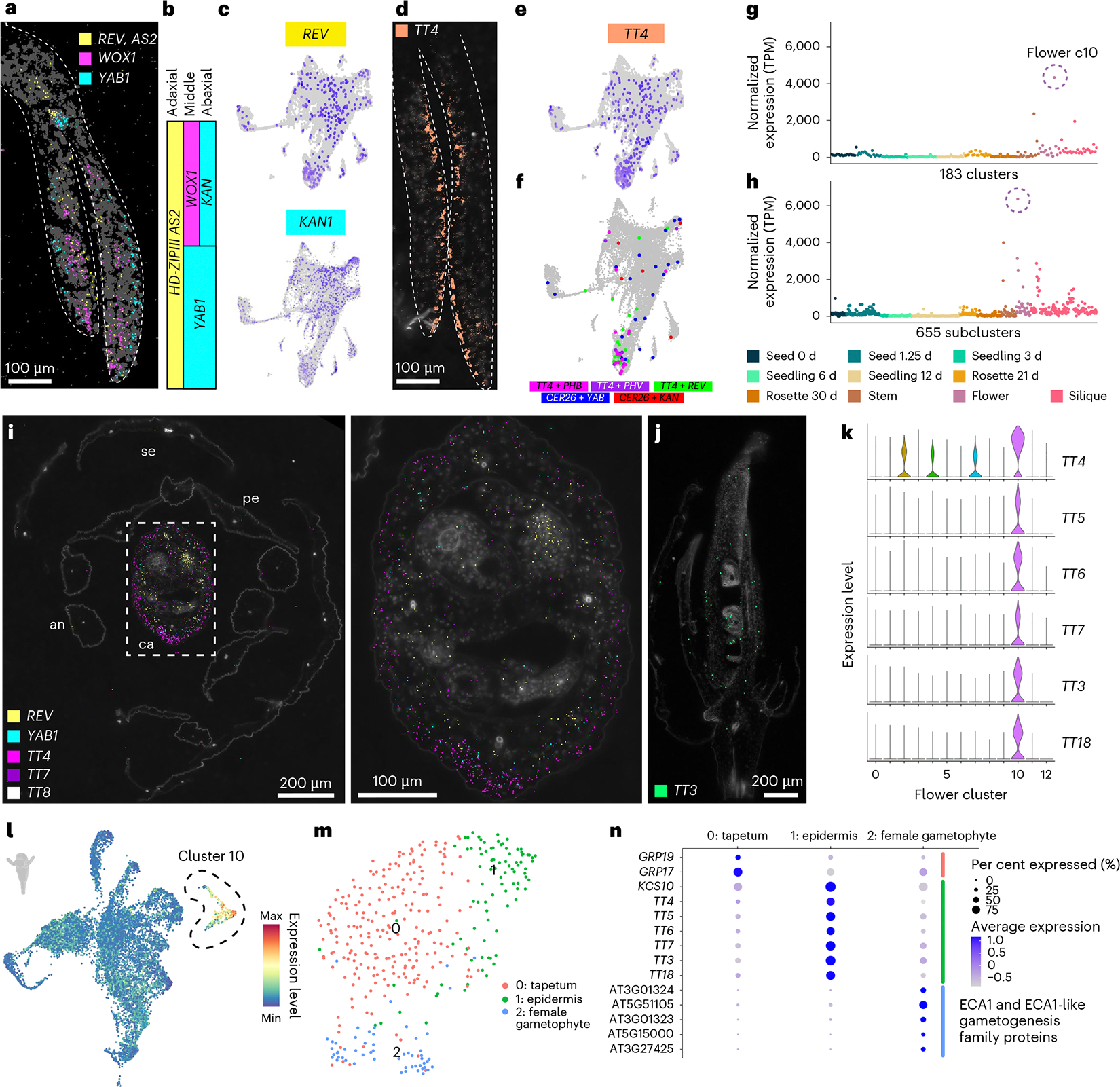
Identification of diverse cellular states throughout development. **a**, Spatial locations of transcripts of the three-domain model of leaf development in cotyledons of the three-day-old seedling spatial transcriptome dataset. The scale bar length is shown. **b**, Simplified three-domain model of leaf development^5^. **c**, Expression of organ polarity regulators *REV* and *KAN1* in the single-nucleus three-day-old seedling dataset. **d**,**e**, Expression of *TT4* in the spatial (**d**) and single-nucleus (**e**) datasets of three-day-old seedlings. The scale bar length is shown. **f**, Identification of nuclei with co-expression of canonical polarity regulators and transcripts with polar expression patterns in the three-day-old seedling spatial transcriptome dataset. **g**,**h**, Normalized pseudobulk expression of TT4 in all 183 major clusters (**g**) and all 655 subclusters (**h**). The points are coloured according to dataset. TPM, transcripts per million. **i**, Spatial expression of organ polarity regulators *REV* and *YAB1* and flavonoid biosynthesis enzymes *TT4*, *TT7* and *TT8* in a flower cross section. Right, zoomed-in image of the carpel. The scale bar lengths are shown. se, sepal; an, anther; pe, petal; ca, carpel. **j**, Spatial expression of *TT3* in a longitudinal section of a flower. The scale bar length is shown. **k**, Expression of the flavonoid biosynthesis enzymes *TT4–7*, *TT3* and *TT18* in clusters of the single-nucleus flower dataset. **l**, Average expression of all enzymes of the flavonoid biosynthesis pathway in the flower single-nucleus dataset. Cluster 10, which shows restricted expression of these transcripts, is circled. **m**,**n**, Subcluster results and expression of cell-type markers in cluster 10 of the flower dataset. For the micrographs in **a** and **d**, results were observed in eight seedlings. For the micrographs in **i** and **j**, results were observed in two cross-sectioned flowers and one longitudinally sectioned flower.

To overcome this limitation, we leveraged our spatial transcriptomics dataset and examined the expression of known polarity regulators in our three-day-old seedling dataset. We detected transcripts of known polarity regulators in expected regions of cotyledons and were able to spatially reconstruct the three-domain model of leaf development^57,58^ with the detection of *REVOLUTA* (*REV*; AT5G60690), *WUSCHEL-related homeobox 1* (*WOX1*; AT3G18010) and *YABBY1* (*YAB1*) / *ABNORMAL FLORAL ORGANS* (*AFO*) (AT2G45190) in the adaxial, middle and abaxial domains of cotyledons, respectively (Fig. 4a–c).

We identified new marker genes that were spatially co-expressed in regions of known polarity regulators, as exemplified by *TT4*, which was detected prominently and solely within the adaxial region of cotyledons (Fig. 4d and Supplementary Fig. 8). Furthermore, *TT4* transcripts were more abundant than those of the canonical adaxial polarity regulators *REV* and *AS2* (Fig. 4a,d), which could be because canonical polarity regulators encode transcription factors that are known to be expressed at lower levels in cells. Despite low detection of canonical polarity regulators in our single-nucleus datasets (Fig. 4c and Supplementary Fig. 7), we were able to identify nuclei that co-expressed polarity regulators with *TT4* (Fig. 4e) and other spatially validated transcripts with polar expression patterns for both abaxial and adaxial defined cells (Fig. 4f and Supplementary Fig. 9), in addition to known cell-type markers (Supplementary Fig. 10). These results demonstrate that these new markers with polar expression patterns in cotyledons denote polarity-defined cells (polarity positive), which is supported by the observation that the mutation of individual *TRANSPARENT TESTA* genes affects the development of specific cell types in seedlings^59^.

While the polarity-positive nuclei were not restricted to individual clusters (Fig. 4f), we instead found that the expression of canonical and newly identified polarity markers was more defined at the subcluster level (Fig. 4g,h). Together, these results reveal that polar specification demarcates cell subpopulations within individual cell types and may further explain the observed subcluster diversity (Supplementary Fig. 5).

### Organ-specific arrangement of cell identities

While *TT4* exhibited prominent adaxial localized expression in cotyledons of three-day-old seedlings, examining *TT4* expression across all organs and developmental stages revealed that *TT4* expression was the greatest in several flower clusters (Fig. 4g,h), which prompted us to examine the expression and spatial distribution of *TT4* within flowers. Consistent with prior findings, we found that transcripts of canonical polarity markers were spatially restricted to the expected adaxial (*REV*, yellow) and abaxial (*YAB1* (ref. 60), cyan) regions of the carpel (Fig. 4i). Interestingly, in contrast to our findings in cotyledons (Fig. 4a), we found that *TT4* transcripts were instead localized to abaxial layers of carpels (Fig. 4i). As *TT4* encodes the sole enzyme involved in chalcone synthesis^50^ upstream of flavonoid biosynthesis, we hypothesized that genes encoding other enzymes in the flavonoid biosynthetic pathway may also be spatially regulated in flowers. We found that the expression of other flavonoid biosynthesis genes (*TT3–7*, *LEUCOANTHOCYANIDIN DIOXYGENASE* (*LDOX*) / *TT18* (AT4G22880) and *FLAVONOL SYNTHASE 1* (*FLS1*; AT5G08640)) was enriched in the same flower cluster (Fig. 4k,l; cluster 10) and spatial regions as that of *TT4* (Fig. 4i,j), which suggests that the expression of entire secondary metabolite biosynthesis pathways may influence the transcriptional identity of individual cells (Fig. 4k). While we observed the highest expression of the flavonoid biosynthetic pathway within flower subclusters, some (but not all) organs were associated with clusters that specifically express flavonoid biosynthesis genes, revealing specific roles of flavonoids in specific organs throughout the *Arabidopsis* life cycle (Supplementary Fig. 11).

Because flavonoids have diverse roles in several biological processes in various flower tissues^61^, we questioned whether the flower cluster associated with high expression of flavonoid biosynthesis enzymes, which we consistently observed with altered clustering parameters (Supplementary Figs. 12 and 13), may instead represent diverse cell types that all produce flavonoids in contrast to a single cell type. Subclustering of the flavonoid biosynthetic cluster (cluster 10; Fig. 4m) revealed three subcluster populations that were enriched for markers of tapetum^62^ (subcluster 0, *GLYCINE RICH PROTEINS*), epidermal^63^ (subcluster 1, *3-KETOACYL-COA SYNTHASE 10* (*KCS10*; AT2G26250) and *TRANSPARENT TESTA* genes) and female gametophyte^64^ (subcluster 2, *ER-TYPE Ca*^*2*+^*-ATPase 1* (*ECA1*) and *ECA1*-like gametogenesis family genes) cells (Fig. 4m,n). Together, these results highlight an example of transcriptional complexity driven by the expression of secondary metabolism pathways among several cell types, where cell-type identity is resolved only at the resolution of subclusters.

### Cell-type-specific and organ-specific genes define the transcriptional identities of cells

**Fig. 5 |:**
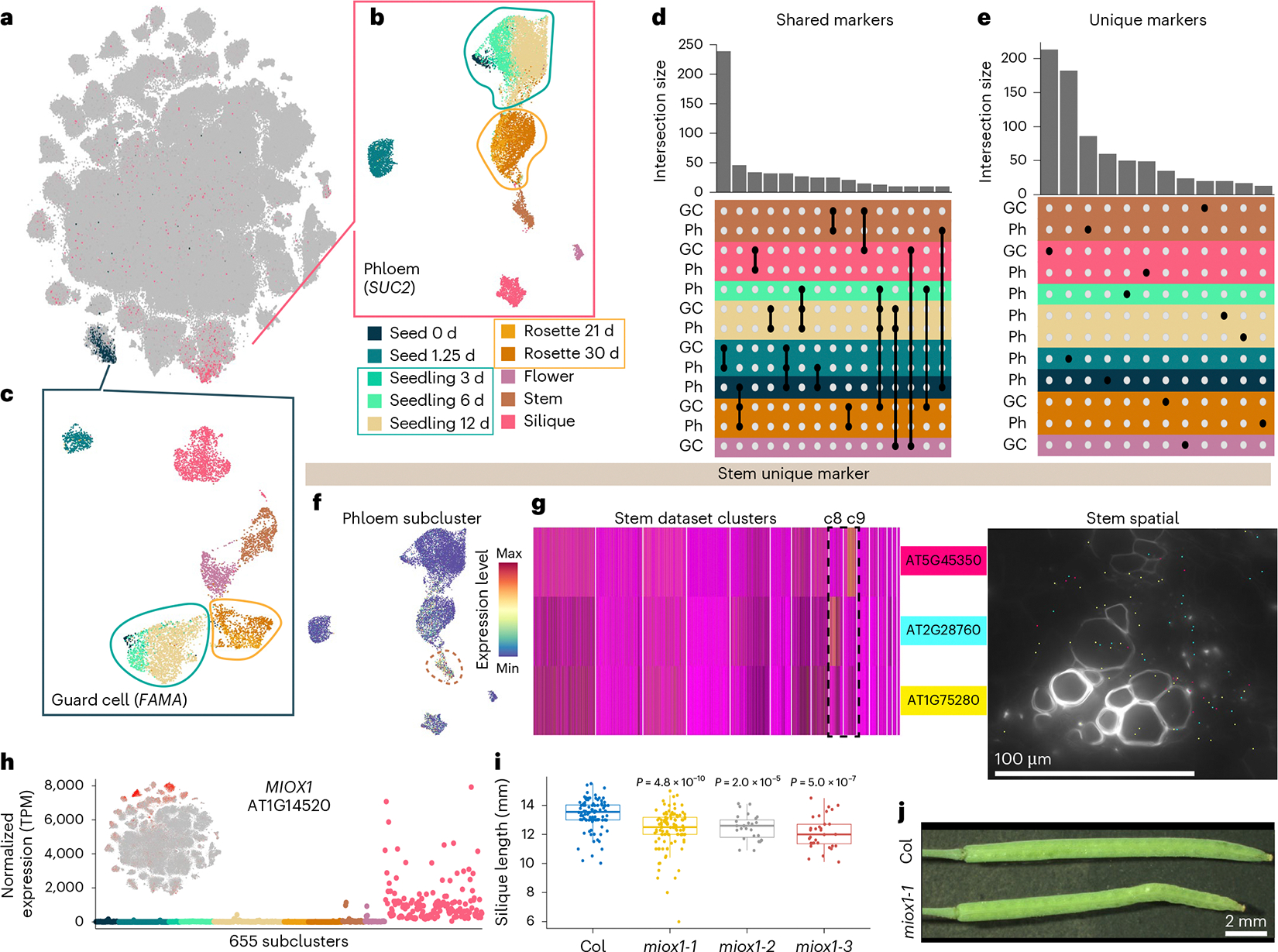
Identification and functional analysis of genes with unique cell-type-specific expression patterns. **a**, *t*-distributed stochastic neighbour embedding of the global dataset with expression levels of the stomatal lineage marker *FAMA* (blue, AT3G24140) and the phloem marker *SUC2* (pink, AT1G22710). **b**,**c**, Re-clustering of the phloem (**b**) and guard cell (**c**) lineage clusters. Cells are coloured according to tissue of origin. Seedling-derived and rosette-derived clusters are circled in blue and orange, respectively. **d**, Quantification of subcluster markers shared between phloem and guard cell lineage subclusters. Groups of subclusters with more than ten overlapping subcluster markers are depicted. The black dots indicate the overlapping groups of subclusters. Colour indicates the dataset of origin with the highest proportion of cells for each subcluster. **e**, Quantification of markers uniquely identified as subcluster markers in only phloem and guard lineage cell subclusters. Subclusters with at least ten uniquely identified genes are depicted. **f**,**g**, Expression of markers uniquely identified in phloem companion cells of the stem dataset. Panel **f** shows the average expression of all markers uniquely identified in stem phloem companion cells. Cluster 9 of the phloem companion cell analysis (Fig. 5b) with maximal expression is circled. Panel **g** shows the expression of three stem phloem companion cell markers in the single-nucleus dataset (left) and the magnified region depicted in Fig. 1e of a vascular bundle in the stem spatial transcriptomic dataset (right). The scale bar length is shown. **h**, Normalized pseudobulk expression of *MIOX1* (AT1G14520) across 655 subclusters. The points are coloured according to dataset. **i**, Quantification of the length of fully elongated siliques in Col-0 and three *miox1* T-DNA mutants. *n* > 9 individual fully elongated siliques were measured from individual plants for each genotype. Siliques from eight plants were measured for Col-0 and the *miox1–1* mutant, and siliques from two plants were measured for the *miox1–2* and *miox1–3* mutants. Silique measurements are displayed as box plots; the centre line indicates the median value, the box boundaries represent the 25th and 75th percentiles, and the whiskers extend to the minimum and maximum values within 1.5 times the interquartile range. The unpaired Wilcoxon test *P* value is reported for each *miox1* mutant compared to Col-0. **j**, Representative image of fully elongated siliques in Col-0 and the *miox1–1* T-DNA mutant. The scale bar length is shown. For the micrograph in **g**, results were observed in one stem.

In the integrated global dataset (Fig. 1f), we identified at least ten clusters that correspond to recurrent cell types, such as vascular (phloem), epidermal (epidermis, guard cells and trichomes) and meristematic (procambium) cell types, that are represented by nuclei from all tissues and developmental time points assayed in which those cell types are present (Supplementary Fig. 14a,b). While these recurrent cell types could be identified by the cluster-specific expression of individual or suites of cell-type markers, we questioned whether heterogeneity within individual cell types may exist at the whole-transcriptome level. We initially focused on phloem companion and guard cells that were identified by the specific expression of the phloem and guard cell marker genes *SUC2* (AT1G22710; refs. 41,42) and *FAMA* (AT3G24140; ref. 65), respectively (Fig. 5a). Independent subclustering of the phloem companion (Fig. 5b) and guard cell populations from the global dataset (Fig. 5c) revealed subcluster complexity within both of these cell types. We observed similar trends within both the phloem companion and guard cell populations, where nuclei from both the seedling and rosette time-series datasets formed individual aggregate clusters (Fig. 5b,c; circled). In contrast, nuclei from other organs, exemplified by silique nuclei, generally clustered separately (Fig. 5b,c), which suggests that organ-specific transcripts contribute to the observed complexity within individual cell types. Examining populations of other recurrent cell types identified from the global dataset (Supplementary Fig. 14a–c) consistently revealed similar trends where silique nuclei clustered separately from those of different organs. From these findings, we hypothesized that the observed heterogeneity within cell types may be due to (1) genes with expression that is restricted to individual organs or developmental time points yet expressed within multiple cell types, or (2) genes with expression under the dual regulation of cell-type and organ or developmental specificity.

To investigate this question, we examined the intersection of newly identified markers from the subsets of phloem companion and guard cells. This revealed two classes of marker genes: (1) markers that were shared across cell types within the same tissue, organ or organ system (shared markers; Fig. 5d and Supplementary Table 4) and (2) unique cell-type markers that were identified within only phloem companion or guard cell populations of individual sample types (unique; Fig. 5e and Supplementary Table 4). For example, *CYTOCHROME P450, FAMILY 709, SUBFAMILY B, POLYPEPTIDE 1* (*CYP709B1*; AT2G46960) and *ARABIDOPSIS NAC DOMAIN CONTAINING PROTEIN 29* (*ANAC029*; AT1G69490) were both identified as markers within silique guard cells (Supplementary Fig. 14d, left).

Examining the expression of these silique guard cell markers in the context of whole-plant development, we found that *CYP709B1* was restricted to the silique organ but was expressed broadly across cell types (shared marker gene), while *ANAC029* expression was unique to silique guard cells within the context of whole-plant development (unique marker gene; Supplementary Fig. 14d, right). Consistent with the broad expression of *CYP709B1* across silique cell types, we also identified shared markers of other tissues in the phloem companion and guard cell populations (Fig. 5d and Supplementary Fig. 14e). Together, these results demonstrate that transcriptional identities within individual cell types are defined both by the expression of genes shared among cell types within a tissue or organ (for example, flower specific) and by genes under the combined regulation of cell-type and developmental specificity (for example, expression restricted to only one cell type in only one organ, such as silique mesocarp). Extending this cell-type-level analysis to other recurrent cell populations revealed similar results (Supplementary Fig. 14f,g and Supplementary Table 4), demonstrating that this observation was not unique to the cell types of phloem companion and guard cells but is instead widespread across diverse cell types.

To systematically identify organ- and development-specific markers across *Arabidopsis* development, we compared the pseudobulk expression of marker genes of all 183 major clusters (Supplementary Fig. 15a, calculated from Supplementary Tables 5 and 6; Methods). Of 4,528 genes identified as markers of at least one of the major clusters, 331 (7.3%) genes were uniquely identified as markers within only one dataset (Supplementary Table 4). Consistent with the unique expression patterns in the pseudobulk data, plotting the expression of these markers both in their natively expressed dataset (for example, stem phloem; Fig. 5f,g) and in the globally integrated dataset revealed that the expression of many of these markers (>70%) was restricted to individual clusters and/or subsets of clusters (Supplementary Fig. 15b), demonstrating that our atlas identifies transcripts uniquely expressed in certain cell types or cell states, present only within specific datasets.

### Functional characterization of a new cell-type-specific gene in *Arabidopsis* siliques

**Extended Data Fig. 7 |:**
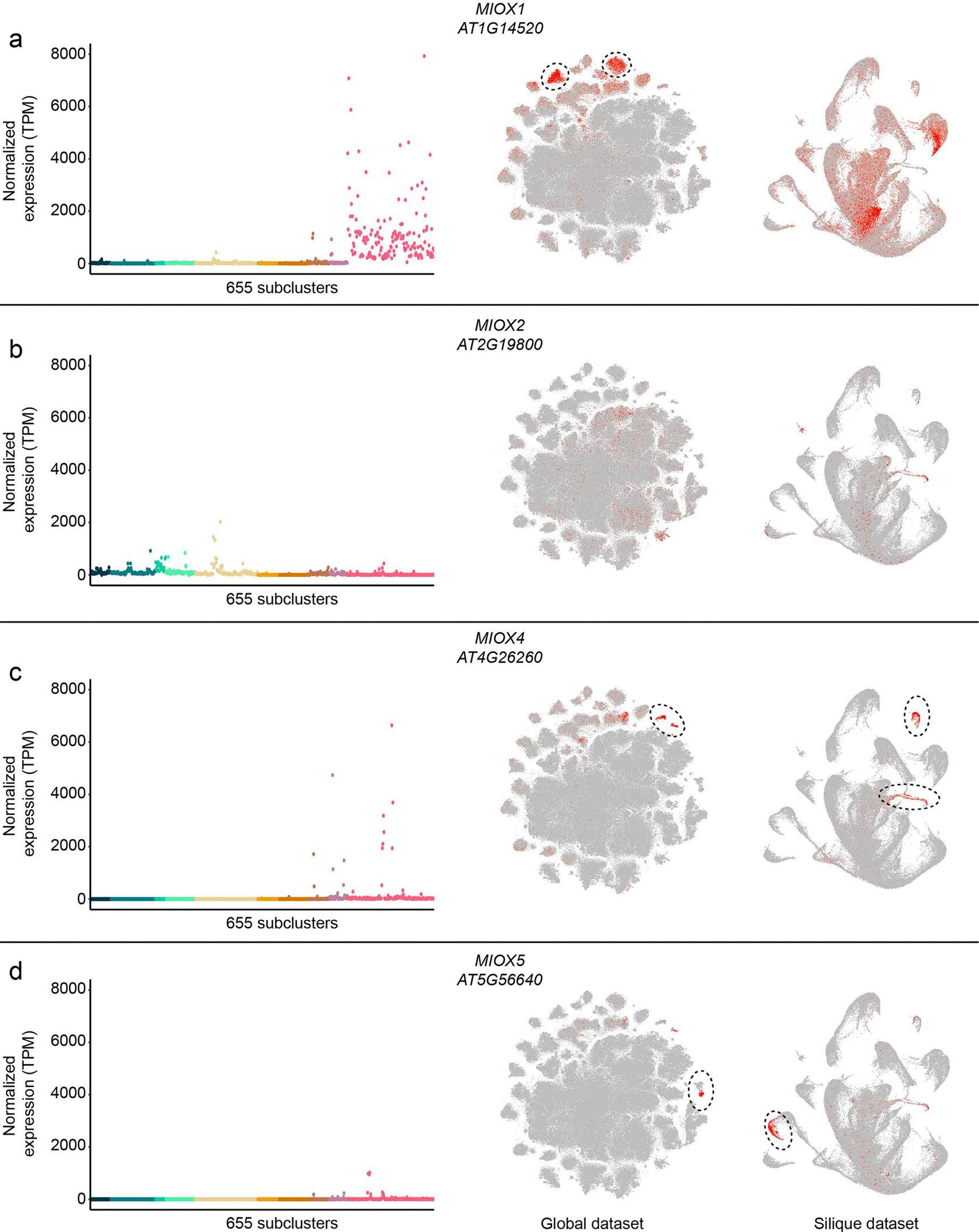
Expression of *myo*-inositol oxygenase (*MIOX*) family members are expressed in distinct cell populations throughout the Arabidopsis life cycle. **a** to **d**, Expression of the four *MIOX* family members (*MIOX1*, *MIOX2*, *MIOX4*, and *MIOX5*) within (left) the 655 identified subclusters and visualized within the (middle) global and (right) silique datasets. Clusters with high expression are circled for emphasis.

We next questioned whether genes with unique expression patterns may have functional relevance within the natively expressed sample type. We evaluated 94 transfer DNA (T-DNA) mutant alleles^66^ of 46 marker genes uniquely identified or enriched as cluster or subcluster markers within individual datasets (Fig. 5e, Supplementary Fig. 15c and Supplementary Table 4). From these mutants, we identified diverse phenotypes for seven genes (15%), including alterations in leaf morphology and physiology, petiole length, bolting time, premature leaf senescence and abnormalities in silique length and morphology (Fig. 5i,j and Supplementary Fig. 15c). Of note, six of the seven genes were not previously associated with phenotypes, demonstrating the capacity to identify new phenotypes in mutants of genes with unique expression patterns. As an example, we focused on mutants of *MYO-INOSITOL OXYGENASE 1* (*MIOX1*; AT1G14520), where we found that high expression of this gene was restricted to the silique dataset and was enriched within individual subclusters of this organ (Fig. 5h). In *miox1* mutants, we observed a reduction (~1 mm) in the length of fully elongated siliques (Fig. 5i,j) without observing extraneous phenotypes in other tissues and developmental stages. These results were consistent in several *miox1* mutants, suggesting a unique cell-type-specific role of *MIOX1* in silique development throughout the *Arabidopsis* life cycle. Examining the expression of the other *MIOX* family members revealed similar trends of subcluster-specific expression in siliques for *MIOX2* (AT2G19800), *MIOX4* (AT4G26260) and *MIOX5* (AT5G56640) (Extended Data Fig. 7), although in contrast to *MIOX1*, the expression of the other *MIOX* family members was instead restricted to other clusters and cell types in the silique dataset, which may suggest specific yet distinct roles of *myo*-inositol metabolism enzymes during silique development.

### The hypocotyl hook as a model of spatiotemporally regulated cellular states

**Fig. 6 |:**
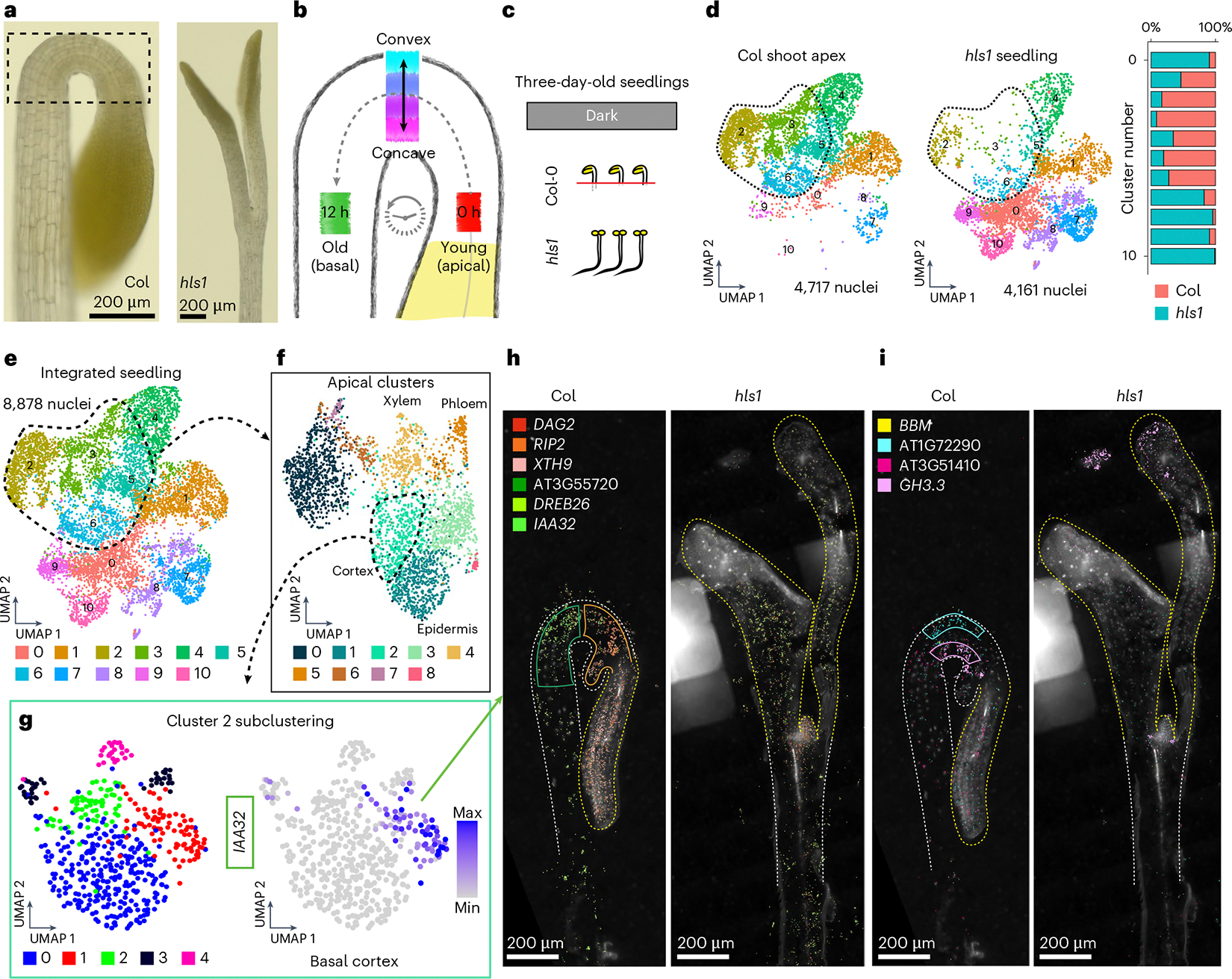
The apical hook as a model of transient cellular states. **a**, Bright-field images of the shoot apex of three-day-old dark-grown seedlings of Col-0 (left) and *hls1* (right). The scale bar lengths are shown. **b**, Model of cellular states associated with the development and maintenance of the apical hook. Maximal activity levels of ethylene and auxin are localized to the convex and concave apices of the apical hook, respectively. Domains associated with the apical–basal axis of development from the shoot apical meristem to the basal hypocotyl are also depicted. **c**, Illustration of the experiments performed. Nuclei were isolated from three-day-old seedlings, with dissected shoot apex tissues collected from Col-0 and whole seedlings from *hls1*. **d**,**e**, Clustering of Col-0 shoot apex cells and whole *hls1* seedlings. In **d**, nuclei from both datasets are plotted separately within the co-embedded UMAP space. The number of nuclei for each genotype among all clusters is depicted as a bar plot (right). Clusters that are overrepresented for cells of the apical hook and underrepresented in *hls1* are circled in red. In **e**, all nuclei from the Col-0 shoot apex and *hls1* seedlings are depicted. **f**, Re-clustering of clusters that majorly represent cells of the apical hook structure. Annotations of select clusters are depicted. A cluster corresponding to cortex cells is circled. **g**, Left, re-clustering of the cortex cell cluster. Right, expression of a newly identified cortex subcluster marker, *IAA32* (AT2G01200). The colour bar represents the expression level. **h**,**i**, Spatial locations of transcripts associated with cellular states in the apical hook. The shoot apices of three-day-old dark-grown seedlings of Col-0 (left) and *hls1* (right) are depicted. Transcripts with spatial expression patterns enriched within domains of the apical–basal axis of development (**h**) and the convex–concave domains of the apical hook (**i**) are depicted. The scale bar lengths are shown. For the micrographs in **a**, **h** and **i**, results were observed in at least eight seedlings for each genotype.

In dicotyledonous plants, the hypocotyl apical hook is a hallmark phenotype of dark-grown (etiolated) seedlings, which is necessary to prevent mechanical damage to the stem cells within the shoot apical meristem as seedlings emerge from soil^67^. To maintain the apical hook structure (Fig. 6a), it is understood that the opposing activity of the hormones auxin and ethylene causes epidermal and cortex cells to transiently undergo differential rates of cell elongation along the convex–concave axis, but as a ‘standing wave’ of growth^68^, where individual cells transit from the shoot apical meristem, progress through and ultimately exit the apical hook in fewer than 12 hours^69^ (Fig. 6b). The transient positions of all individual cells as they pass through the apical hook thus represent unique cellular states at the intersection of developmental and hormonal regulation. We therefore sought to use the apical hook as a model to study cellular states.

To provide a foundation for identifying cellular states within the apical hook, we generated paired single-nucleus and MERFISH datasets of dissected shoot apices containing the apical hook from wild-type seedlings, as well as whole seedlings of *hookless1* (*hls1*; AT4G37580), which undergoes normal seedling development but specifically lacks the apical hook structure^70^ (Fig. 6c). Co-clustering of these datasets revealed that specific clusters (clusters 2, 3, 5 and 6) were overrepresented by nuclei of the Col-0 shoot apex and underrepresented in *hls1* seedlings, revealing that these clusters represent cells in the apical hook (Fig. 6d,e).

**Extended Data Fig. 8 |:**
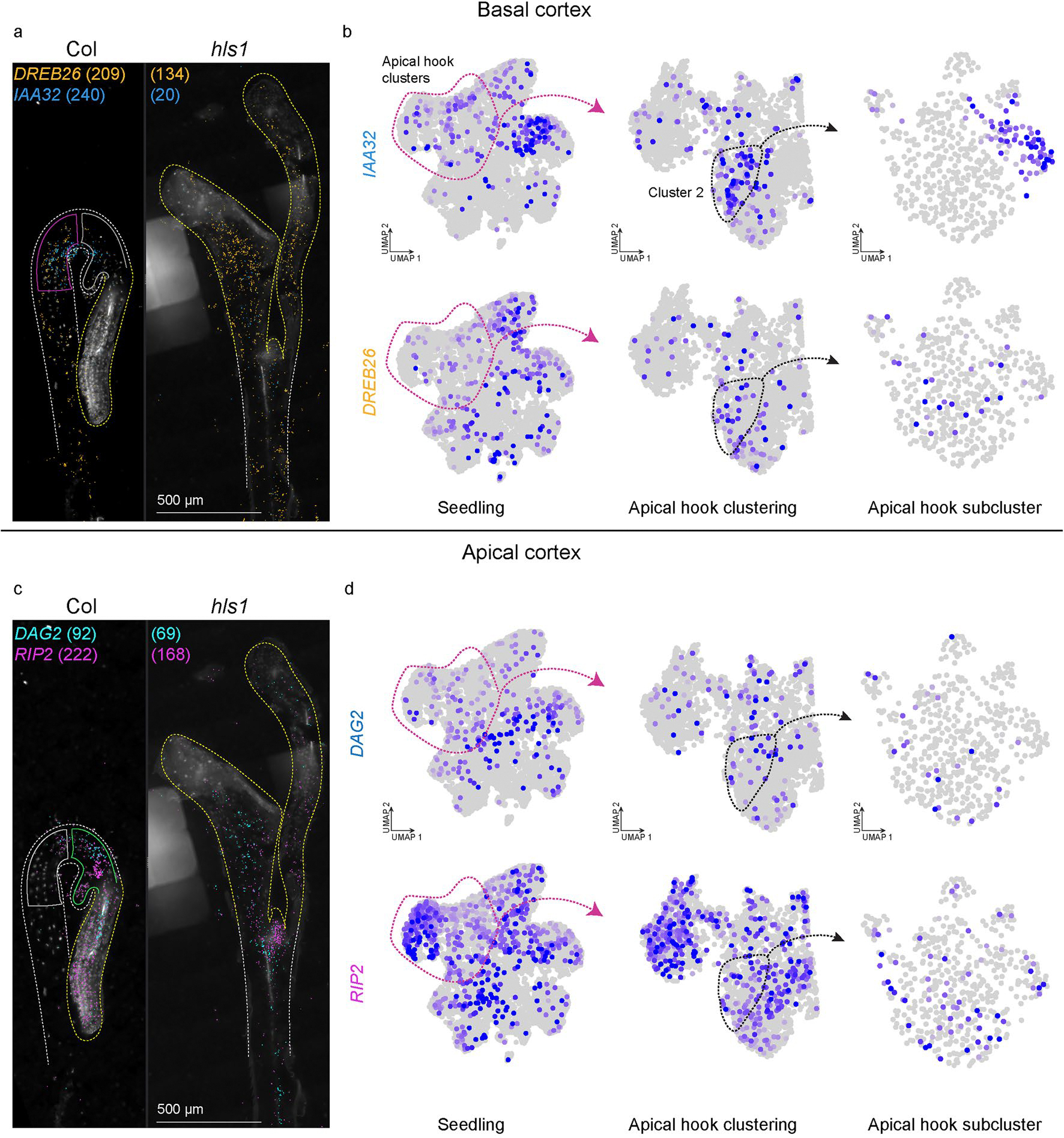
Subclusters within the apical hook dataset correspond to spatially regulated cellular states of cortex cells associated with the apical-basal axis of development. **a** and **b**, Expression of *IAA32* (*AT2G01200*) and *DREB26* (*AT1G21910*) that are spatially restricted to the basal hypocotyl cortex cells within the apical hook. (**a**) Spatial location of *DREB26* and *IAA32* transcripts in three-day-old etiolated seedlings of (left) Col-0 and (right) *hls1*. Quantification of *DREB26* and *IAA32* mRNA molecules in hypocotyls of Col and *hls1* is depicted in parentheses. (**b**) Expression of (top) *IAA32* and (bottom) *DREB26* within the (left) whole seedling clustering, (middle) apical hook re-clustering, and (right) subclustering of individual apical hook clusters. **c** and **d**, Expression of *DAG2* (*AT2G46590*) and *RIP2* (*AT2G37080*) that are spatially restricted to the cortex cells within the apical region of the shoot apex. (**c**) Spatial location of *DAG2* and *RIP2* transcripts in three-day-old etiolated seedlings of (left) Col-0 and (right) *hls1*. Quantification of *DAG2* and *RIP2* mRNA molecules in hypocotyls of Col and *hls1* is depicted in parentheses. (**d**) Expression of (top) *DAG2* and (bottom) *RIP2* within the (left) whole seedling clustering, (middle) apical hook re-clustering, and (right) subclustering of individual apical hook clusters. Scale bar = 500 μm. For the micrographs depicted in (**a**) and (**c**), results were observed in at least three seedlings per genotype.

**Extended Data Fig. 9 |:**
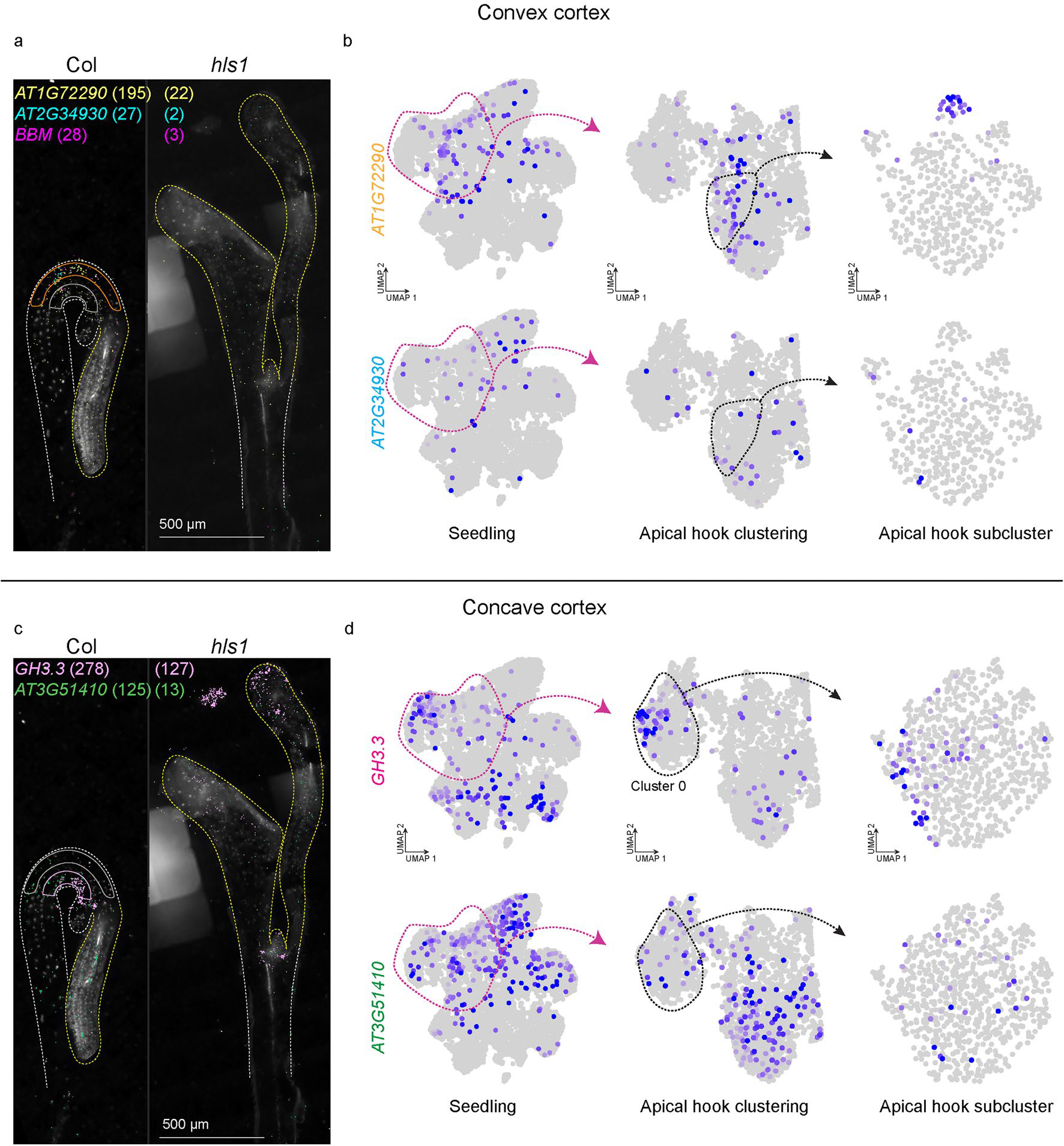
Subclusters within the apical hook dataset correspond to spatially regulated cellular states of cortex cells associated with convex-concave regions of the apical hook. **a** and **b**, Expression of *AT1G72290*, *AT2G34930*, and *BBM* (*AT5G17430*) that are spatially restricted to the hypocotyl cortex cells within the convex region of apical hook. (**a**) Spatial location of *AT1G72290*, *AT2G34930*, and *BBM* transcripts in three-day-old etiolated seedlings of (left) Col-0 and (right) *hls1*. Quantification of *AT1G72290, AT2G34930, and BBM* mRNA molecules in hypocotyls of Col and *hls1* are depicted in parentheses. (**b**) Expression of (top) *AT1G72290* and (bottom) *AT2G34930* within the (left) whole seedling clustering, (middle) apical hook re-clustering, and (right) subclustering of individual apical hook clusters. **c** and **d**, Expression of *GH3.3* (*AT2G23170*) and *AT3G51410* that are spatially restricted to the cortex cells within the concave region of the apical hook. (**c**) Spatial location of *GH3.3* and *AT3G51410* transcripts in three-day-old etiolated seedlings of (left) Col-0 and (right) *hls1*. Quantification of *GH3.3* and *AT3G51410* mRNA molecules in hypocotyls of Col and *hls1* is depicted in parentheses. (**d**) Expression of (top) *GH3.3* and (bottom) *AT3G51410* within the (left) whole seedling clustering, (middle) apical hook re-clustering, and (right) subclustering of individual apical hook clusters. Scale bar = 500 μm. For the micrographs depicted in (**a**) and (**c**), results were observed in at least three seedlings per genotype.

Re-clustering of the apical hook cells revealed additional clusters that correspond to known cell types in hypocotyls (Fig. 6f). Further investigation of heterogeneity within cell types through subclustering analyses revealed a total of 24 subclusters that may represent distinct cellular states in all cell types present in the apical hook. To focus on cell states that may regulate differential growth programs within the apical hook, we focused on cortex clusters, which represent the most abundant cell type in the apical hook by volume^71^. Subclustering of cortex cluster 2 revealed five discrete cell populations in this cluster (Fig. 6g), where we identified *INDOLE-3-ACETIC ACID INDUCIBLE 32* (*IAA32*; AT2G01200) as a subcluster marker with known expression in the basal concave region of the apical hook^72^, which we also confirmed in our spatial datasets (Fig. 6h and Extended Data Fig. 8a). We also identified new markers with spatial expression restricted to cortex cells in convex and concave regions of the apical hook (Fig. 6h), as well as along the apical–basal axis of development (Fig. 6i). Additionally, expression of these markers was absent or reduced in straightened hypocotyls of *hls1* (Extended Data Figs. 8 and 9), further suggesting a role of cell-state markers in apical hook regulation.

While we were able to identify new markers of apical–basal and convex–concave positioning, we observed varying levels of co-expression of these markers both spatially and at the single-cell level, which suggests further spatial complexity in this tissue structure. This observation is exemplified by the basal apical hook cortex markers *IAA32* and *DEHYDRATION RESPONSE ELEMENT-BINDING PROTEIN 26* (*DREB26*; AT1G21910), where we observed that *IAA32* expression is uniquely restricted to the basal apical hook, while *DREB26* expression is similarly expressed in the basal apical hook but also extends into the basal hypocotyl (Extended Data Fig. 8a). This observation is also reflected in the apical hook single-nucleus dataset, where we observed *IAA32* and *DREB26* co-expression in the same apical hook cortex cluster (cluster 2; Extended Data Fig. 8b, middle), but *IAA32* and *DREB26* expression becomes more distinct in apical hook cortex subclusters (Extended Data Fig. 8b, right). Together, these results highlight the fine-tuned spatiotemporal regulation of cortex cell neighbourhoods in the apical hook and suggests that *IAA32* and other markers of cellular states in the apical hook may be directly involved in the regulation of differential growth to maintain this structure.

**Extended Data Fig. 10 |:**
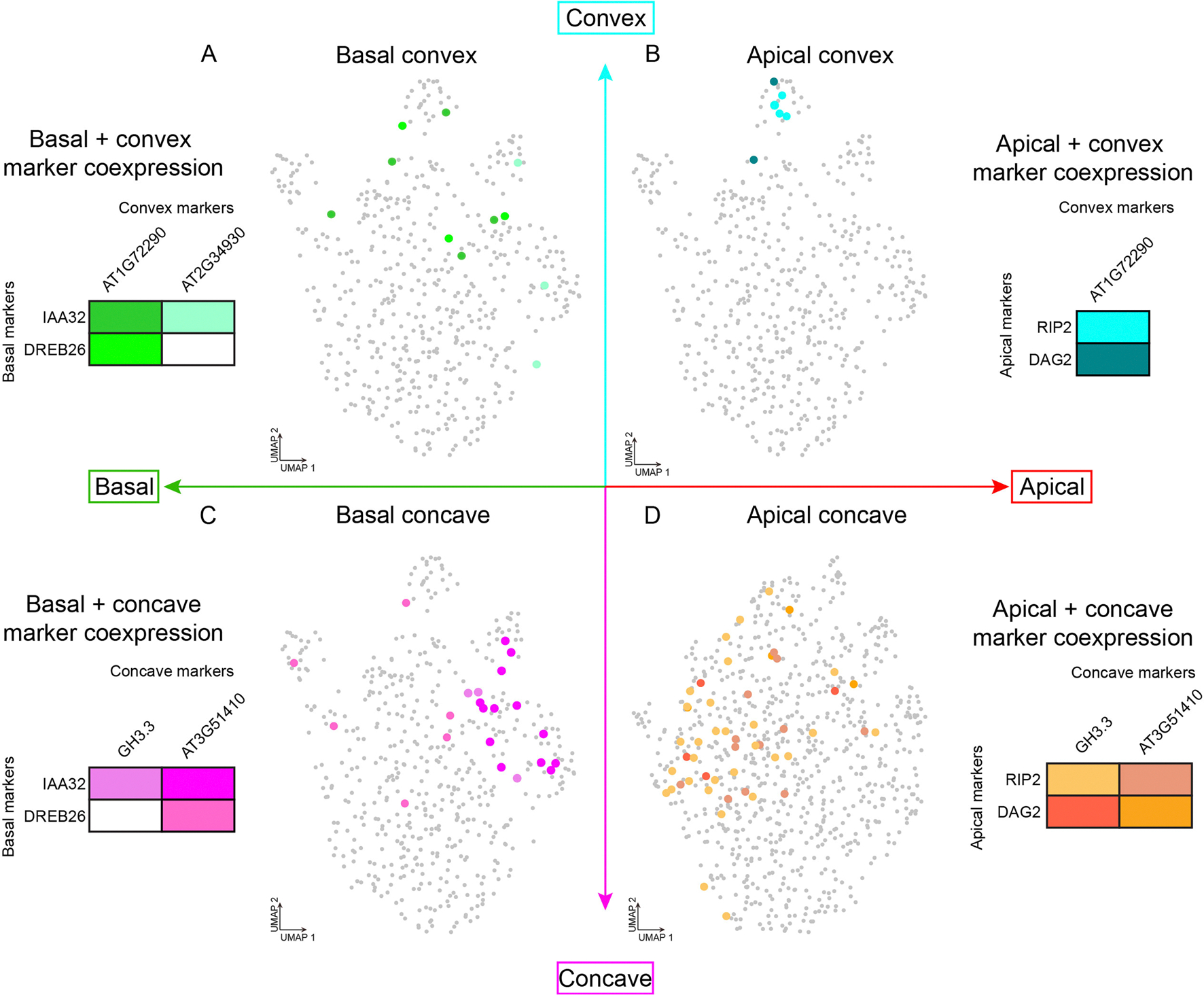
Cellular states defined by the combined regulation of development (apical-basal axis) and hormonal signaling (convex-concave axis) is associated with subclusters of cortex cells. **a** to **d**, Cortex cells that co-express combinations of markers associated with apical-basal or convex-concave regions within the apical hook. Apical markers: *DAG2* and *RIP2*. Basal markers: *DREB26* and *IAA32*. Convex markers: *AT1G72290* and *AT2G34930*. Concave markers: *GH3.3* and *AT3G51410*.

Finally, to understand the combinatorial interaction of cellular states that regulate orthogonal spatial axes aligned with developmental progression (apical–basal) and lateral asymmetry that underlies tissue bending (convex–concave), we examined cells that co-express markers of apical–basal and convex–concave patterning, which represent rare cell neighbourhoods of ten or fewer cells within a whole organism (for example, convex cells of the basal hypocotyl) (Extended Data Fig. 10). Independent analysis of all four cell groups revealed that markers of each of these populations were enriched for distinct and diverse Gene Ontology (GO) terms, many of which are associated with the regulation of growth or development, such as anatomical structure arrangement and pattern specification process, which are associated with apical–convex and apical–concave cell states, respectively (Supplementary Fig. 16). Furthermore, several newly identified markers in enriched GO categories for all cellular states analysed have demonstrated function in apical hook regulation. Our comprehensive analysis of cellular states within the apical hook thus revealed diverse cellular states, represented by over 20 subclusters, as well as a role of spatiotemporally regulated cell-state genes, with pinpoint precision of expression, in wild-type growth and development of the apical hook.

## Discussion

In this study, we used single-nucleus and spatial transcriptomic technologies to characterize cellular identities along the entire life cycle of the model plant *Arabidopsis*. As a resource of broad interest, complementary application of single-nucleus and spatial transcriptomic technologies enabled the annotation of known and new cell types and regions (Figs. 2–4) to diverse organs and tissues, many of which have not been investigated at high resolution or at the single-cell level. Our sampling of diverse organs allowed us to evaluate the transcriptomes of recurrent and unique cell populations across organs and developmental stages at the single-nucleus level, which revealed heterogeneity within individual cell types (Fig. 5). With an easily accessible web application, we believe that our life-cycle atlas will be of wide use to the plant science community and scientists interested in single-cell biology for hypothesis generation and reference for future single-cell genomics studies. The integration of our data with published single-cell protoplast-based datasets (Supplementary Fig. 17) demonstrates that our atlas can function as reference datasets onto which future single-cell omics data can be integrated to further annotate cell types and/or investigate cell-type-specific responses to environmental or chemical stimuli. Furthermore, proof-of-concept analyses demonstrate that spatial single-cell analyses are feasible using our datasets (Supplementary Figs. 18 and 19).

Across the ten developmental time points assayed, we identified immense transcriptional diversity represented by 183 major clusters and 655 subclusters. In our datasets, we identified known cell types and, with the aid of our paired spatial transcriptomic datasets, were able to more finely annotate cellular populations that were obscured in our single-nucleus datasets, including individual cell layers within seed sacs (Fig. 3) and polarity-defined cells within cell types (Fig. 4). Our life-cycle atlas also highlights a cellular state defined by the expression of enzymes of the flavonoid biosynthetic pathway that promotes co-clustering of distinct cell populations (for example, tapetum, epidermis and gametophyte; Fig. 4) at the transcriptome-wide level of single cells. What remains undetermined is the level of influence of other secondary metabolite pathways and their effect on cellular transcriptomes throughout the *Arabidopsis* life cycle. For each of the 655 individual cell populations, newly identified marker genes may be of use for future characterization of other distinct and potentially new cell populations (Supplementary Fig. 11 and Supplementary Table 3).

Comparative analyses of recurrent cell types across organs are a unique aspect of our developmental atlas compared with atlases of individual organs. By examining recurrent cell types, we revealed that the expression of organ-wide transcripts further influences the transcriptional identity of cells (Fig. 5). The expression of transcripts broadly within organ systems may indicate high levels of coordinated activity across cell types of a tissue, as is the case for flavonoid biosynthesis (Fig. 4), and may contribute to further transcriptional complexity within cell types.

Transcripts uniquely expressed in individual cell populations throughout development are of particular interest, as they may reveal unique functions and gene regulatory mechanisms. Interestingly, cell types of the reproductive tissues were associated with greater quantities of uniquely expressed transcripts than other tissues, which may be attributed to cell types present only within these tissues. A phenotypic investigation of genes with cell-type and developmental specificity revealed that loss-of-function mutants are associated with diverse phenotypes only within the tissue of native expression context (Fig. 5 and Supplementary Fig. 15c). Many of the genes for which we identified new phenotypes are members of multigene families, demonstrating non-redundant functions of individual gene family members within their native expression contexts, as was the case for *miox1*, where we identified a new shortened silique phenotype (Fig. 5i,j). We found that the expression of the remaining members of the *MIOX* family (*MIOX2*, *MIOX4* and *MIOX5*) are all enriched for distinct and separate cell types within siliques (Supplementary Fig. 7), which may suggest broad yet distinct spatially partitioned functional roles of *myo*-inositol metabolism in silique development. Higher-order mutation of *MIOX* family members may provide interesting future opportunities to investigate the role of *myo*-inositol metabolism in silique development in both cell-type-specific and organ-system contexts.

Reproductive organs are associated with the production of diverse and unique secondary metabolites^73–75^, such as flavonoids (Fig. 4), which may also be a basis for the observed unique expression patterns. We found that the expression of enzymes of the full flavonoid biosynthesis pathway was consistently associated with a single cluster in our flower dataset that encompasses epidermal, tapetal and female gametophyte cell types (Fig. 4m,n and Supplementary Figs. 12 and 13). An undefined alteration of cellular status or state within these cell types may occur upstream of flavonoid biosynthesis, and it will be interesting to investigate potential upstream regulatory programs that define this cellular switch in diverse cell types.

Finally, we demonstrated that the hypocotyl apical hook of *Arabidopsis* serves as an exemplary model for the investigation of complex, spatiotemporally regulated cellular states due to its intricate cellular composition and dynamic developmental processes. Our analysis uncovered 24 distinct cell-state subclusters in this compact anatomical structure that are associated with the apical hook’s characteristic differential growth patterns. In cortex cells, which represent the most abundant cell type in the apical hook, we identified new subcluster-specific marker genes that exhibited precise spatial localization to regions of the apical hook, demonstrating clear transcriptional demarcation along both the apical–basal and convex–concave axes. Importantly, the spatial expression of these marker genes was markedly diminished or completely lost in the *hls1* mutant, underscoring their critical roles in hook formation and curvature maintenance.

Further detailed analysis revealed cellular subpopulations co-expressing markers of both axes, such as cells uniquely positioned at intersections of apical–basal and convex–concave developmental gradients. Interestingly, we found that several of the newly identified markers of these cellular neighbourhoods have known functions in apical hook development, demonstrating that the spatiotemporal regulation of cellular states can be functionally relevant to wild-type form and function (Supplementary Fig. 16).

Collectively, our comprehensive analysis demonstrates the intricate cellular architecture and transcriptional specificity throughout the *Arabidopsis* life cycle. The complementary application of single-nucleus and spatial transcriptomic methodologies to diverse samples facilitated the annotation of the majority of clusters (138/183; 75%) in our life-cycle atlas. Several of the samples profiled currently lack comprehensively annotated single-cell reference datasets. As in situ spatial transcriptomic methodologies require knowledge of up to thousands of transcripts with distinct expression patterns, our datasets also function as a foundation for future spatial transcriptomics studies. Overall, our atlas highlights the complexity of cell types present throughout an organism’s entire life cycle. Moreover, this atlas serves as a foundation for future studies that seek to further characterize specific organs and developmental stages or investigate stress or stimulus-driven responses. Researchers can access the processed datasets for rapid exploration in a web-based interface available at our web portal (http://arabidopsisdevatlas.salk.edu/).

## Methods

### Plant growth and sampling

For the imbibed seeds (0 days old), germinating seeds (1.25 days old), light-grown 3-, 6- and 12-day-old seedling developmental time points, and 3-day-old etiolated seedlings, *Arabidopsis* Col-0 seeds (CS6000) were surface-sterilized via incubation in 70% (v/v) ethanol for 5 min, followed by incubation in 50% (v/v) bleach and 0.01% (v/v) TWEEN-20 for 5 min, followed by triplicate washes in double distilled water for 5 min. Seeds of *hls1–1* (CS3073) were sterilized identically. Sterilized seeds were placed onto 1 X Linsmaier and Skoog media (1.0 LS salts (Caisson Labs), 0.8% agar (w/v), 1% w/v Suc., pH 5.7) in 120-mm^2^ petri dishes at a density of 100 seeds per row for the 0-day and 1.25-day samples and 30 seeds per row for the 3-day, 6-day and 12-day samples. The imbibed seeds were stratified via incubation at 4 °C for three days in complete darkness. Following stratification, the plates were placed in a long-day (16 h light/8 h dark) growth chamber at 23 °C. For the Col-0 and *hls1* three-day-old etiolated seedlings, the plates were placed in sealed dark chambers and grown in a growth chamber without lights at 23 °C. For the rosette, flower and silique tissues, seeds were sterilized identically to the seed and seedling samples, directly sown onto soil and grown at 23 °C in long-day conditions.

### Nucleus extraction and single-nucleus sequencing

For all samples, at least two replicates, defined as multiple plates for seed and seedling time points or at least half a flat of plants (>18 plants) for later developmental stages, were prepared independently. Nucleus purification buffer (NPB; 20 mM MOPS (pH 7), 40 mM NaCl, 90 mM KCl, 2 mM EDTA, 0.5 mM EGTA, 0.5 mM spermidine, 0.2 mM spermine, 1:100 protease inhibitor cocktail, 2% BSA, 1:1,000 SUPERase IN) was prepared fresh and chilled on ice. All the following procedures were performed on ice or at 4 °C. Tissues were chopped in 500–1,000 μl of cold NPB with a razor blade on ice for 5 min to release nuclei and incubated in 10 ml of NPB. For the 0-day and 1.25-day seed samples, the nuclei were extracted in 5 ml of NPB with a Dounce homogenizer with 20 strokes of the loose pestle followed by 20 strokes of the tight pestle. The crude nucleus extract was sequentially filtered through 70-μm and 30-μm cell strainers. BSA, Triton X-100 and NP40 were added to final concentrations of 2%, 0.05% and 0.05%, respectively, and the extract was incubated at 4 °C for 10 min with rotation. Nuclei were pelleted via centrifugation at 700 *g* for 5 min with a swing-rotor centrifuge. The pellet was resuspended in 10 ml of NPBd (NPB, 0.05% Triton X-100, 0.05% NP40, 2% BSA) via pipetting and incubated for 10 min, followed by centrifugation at 700 *g* for 5 min. For seedlings and rosettes, additional washes in NPBd were performed until the pellet became translucent. The pellet was then resuspended in 1 ml of NPB and centrifuged at 50 *g* for 3 min in a fixed-rotor centrifuge to pellet non-nucleus debris (including pollen grains from flower tissues), and the supernatant was taken. This step was repeated until the supernatant cleared or visible pellet was not observed. For root-containing tissues (seedlings), the nucleus suspension was filtered through Flowmi strainers (40 μm) to remove additional sources of debris. Finally, nuclei were pelleted via centrifugation at 700 *g* for 10 min with a swing-rotor centrifuge and resuspended in an appropriate volume of NPB. Nuclei were counted manually with a haemocytometer. Nuclei from each replicate were evenly distributed into at least four independent samples, with a targeted recovery of up to 20,000 nuclei per reaction. Each nucleus suspension was loaded into the 10x Genomics Chip M, which was then processed with a 10x Genomics Chromium X controller, and sequencing libraries were constructed following the manufacturer’s instructions. Each sequencing library was sequenced with the Illumina NovaSeq 6000.

### Sample multiplexing

For the stem sample, three regions of the stem (base, branched area and apex) were harvested separately and multiplexed using the 3′ CellPlex Kit following the manufacturer’s specifications (10x Genomics). Briefly, equal quantities of purified nuclei from each sample were individually labelled with separate CellPlex barcodes. Following labelling, the nuclei were pelleted and washed twice with PBS containing 2% BSA with RNase inhibitor. The nuclei were then quantified again using a haemocytometer and were pooled in equal quantities and brought to the appropriate volume for loading into the 10x Genomics Chip M as described previously.

### snRNA-seq pre-processing

Demultiplexed FASTQ files were used to generate gene-by-cell matrices with Cell Ranger (v.7.0.0)^76^ and were aligned to the *Arabidopsis* nuclear transcriptome using the include-introns flag, which was prepared with the TAIR10 genome and Araport 11 transcriptome. Downstream analyses were performed with the R (v.4.1.3)^77^ package Seurat (v.4.1.0)^78^. Low-quality and potential doublet nuclei were removed from the datasets. Nuclei that did not meet a cut-off of >300 and <7,000 genes detected, >400 UMI per nucleus, <5% mitochondrial or <15% chloroplast reads were removed.

### snRNA-seq analysis

For the analysis of single-tissue datasets, after filtering, 2,000 integration features for the aggregated datasets were determined for data scaling and dimensionality reduction using the SelectIntegration-Features function. Each of the datasets was then integrated using the IntegrateData function with 20 principal components (PCs). Following integration of each dataset, the entire dataset was scaled and dimensionality reduction was performed with PC analysis (PCA) followed by UMAP using 30 PCs. Identification of nearest neighbours and clustering were then performed using the FindNeighbors and FindClusters functions, respectively. Cluster markers were then identified for each cluster using the FindAllMarkers function with logfc.theshold = 0.25 and min.pct = 0.1. For iterative subclustering, each individual cluster was subset and re-normalized, 2,000 variable features were identified, the data were scaled, dimensions were reduced via PCA and UMAP using 30 PCs, and neighbours and clusters were identified.

For the globally integrated dataset, all of the individual stringently filtered datasets were merged, and the data were normalized. Following the guidelines of refs. 79,80, 4,000 variable features were identified and used for PCA, and pre-clustering was performed with Harmony (v.0.1.0)^81^ using 50 PCs. *t*-distributed stochastic neighbour embedding was then performed according to ref. 82 using the harmony embeddings with the perplexity value equal to the number of nuclei in the dataset divided by 100 and the learning rate equal to the number of nuclei divided by 12.

### Cell-type annotation

For cell annotation, we used marker genes identified in previous studies using promoters and GUS/GFP fusions, in situ hybridization assays, bulk RNA-seq studies of dissected tissues and single-cell atlases of roots, shoot apical meristem, young leaves and inflorescence stems (Supplementary Table 1). For newly identified marker genes of each cluster, the numbers of curated cell-type markers were divided by the number of cluster marker genes to calculate the cell-type enrichment score. We also explored the expression of top cluster markers in databases such as TAIR (www.arabidopsis.org) and ePlant (bar.utoronto.ca/eplant/). Finally, we used spatial transcriptomics generated during this study for seeds, seedlings, stems, flowers and siliques to validate cell identities and uncover identities of unannotated clusters. For annotating silique major clusters, a curated list of marker genes was used^35^.

### Single-nucleus and single-cell dataset integration and cell-type prediction

To integrate our 21-day-old and 30-day-old rosette single-nucleus datasets with a published leaf protoplast dataset^19^, each dataset was individually loaded, normalized and scaled using SCTransform^83^, and 30 PCs were used for dimensionality reduction. Integration anchors were calculated using the FindTransferAnchors function using the protoplast dataset as the reference dataset. Cell-type label transfer, low-dimensional embedding correction and dataset projection of the nucleus datasets were performed using the MapQuery function.

### Seedling and rosette dataset integration

To integrate the seedling and rosette time-series datasets, each dataset was pre-processed as described previously, and each dataset within the developmental time series was merged. For each merged dataset, PCs were calculated, and Harmony (v.0.1.0)^81^ was used to integrate the datasets regressing variables of dataset input and percentage of mitochondrial and chloroplastic reads, rounded to the nearest integer. Dimensionality reduction, nearest neighbour identification and clustering were then performed using 20 harmony dimensions. Identification of cluster markers was performed as described previously for individual datasets.

### Identification of unique cell population marker genes

First, we identified marker genes for individual clusters of each tissue type (that is, for 183 clusters) using the FindAllMarkers function of Seurat (adjusted *P* < 0.0005, average log_2_(fold change) > 2). We then checked whether these cluster marker genes are highly expressed in other organs using pseudobulk expression data of each cluster. A marker gene candidate was removed when (1) its normalized expression level in the target cluster was not more than fourfold higher than that in any other clusters in the other organs or (2) the log_2_ TPM value of the gene was higher than five in any clusters in the other organs. In this way, we removed genes that are cell-type-specific in a particular organ but are also expressed in other organs. In this analysis, we defined organs in the following way: imbibed and germinating seeds (0 days and 1.25 days), seedlings (3 days, 6 days and 12 days), rosettes (21 days and 30 days), stems, flowers and siliques.

### Cross-tissue cell-type analysis

Clusters identified from the global dataset that were well represented by several datasets were selected and individually subset. Re-clustering was performed as described previously. For cross-tissue comparisons of datasets, the tissue dataset that comprised the majority population of each subcluster was specified as the organ identity for each subcluster. Systematic identification of subcluster markers was performed, and unique and overlapping sets of subcluster marker genes were visualized using ComplexUpset (v.1.3.3)^84^. Intersections of subcluster groups with fewer than ten shared subcluster markers were filtered for visualization.

### GO term enrichment analysis

GO term enrichment of cluster and subcluster markers was performed with ClusterProfiler (v.4.2.2)^85^ using the enrichGO function, and *P* values were adjusted via the Benjamini–Hochberg procedure.

### Silique MERFISH panel design

A list of marker genes of silique major clusters and subclusters were filtered using the following criteria: (1) more than 25 specific probes could be designed on the basis of Vizgen’s software, and (2) the TPM value was below 1,000 in the silique pseudobulk RNA-seq data (obtained by aggregating all the silique cells). After the filtering, 140 genes representing most major clusters and selected subclusters were chosen from the remaining marker genes. The panel design was balanced so that the total TPM value of 140 transcripts was below 11,000 to avoid overcrowding of signal.

### 1,000-target MERFISH panel design

A list of marker genes of three-day-old seedling shoot apex major clusters and subclusters was filtered using the following criteria: (1) more than 25 specific probes could be designed on the basis of Vizgen’s software, and (2) the TPM value was below 1,000 in the three-day-old seedling pseudobulk RNA-seq data^86^. After filtering, 600 genes representing most major clusters and subclusters were selected in addition to 400 transcription factors with expression that was specific to clusters and/or subclusters in the three-day-old seedling dataset. The panel design was balanced so that the total TPM value of 1,000 transcripts was below 15,000 to avoid overcrowding of signal.

### Tissue cryosectioning, fixation and mounting

Plants were grown according to the previously described methods. Tissues of three-day-old etiolated seedlings, the stem apex, mature unpollinated flowers and fully elongated siliques matching the aforementioned developmental stages were excised and immediately incubated in OCT (Fisher) for 5 min to acclimate the tissue. Following incubation, the tissue was placed into cryomolds (Sakura), which were filled with OCT and immediately frozen in an isopentane bath cooled by liquid N_2_. Tissue blocks were acclimated to −18 °C in a pre-cooled cryostat chamber (Leica) for 1 h. The tissue blocks were trimmed until the tissue was entered, after which 10-μm sections were visually inspected until the region of interest was exposed. Sample mounting and preparation were performed according to the MERSCOPE user guide, with slight modifications. Briefly, a 10-μm section was melted and mounted onto a room-temperature MERSCOPE slide (Vizgen, 20400001), placed into a 60-mm petri dish and re-frozen via incubation in the cryostat chamber for 5 min. Subsequent steps were performed with the mounted samples in the petri dish. The samples were then baked at 37 °C for 5 min and incubated in fixation buffer (1× PBS, 4% formaldehyde) for 15 min at room temperature. The samples were then washed with 1× PBS containing 1:500 RNAse inhibitor (Protector RNAse Inhibitor, Millipore Sigma) for 5 min at room temperature in triplicate. Following the final PBS wash, the samples were dehydrated via incubation in 70% EtOH at 4 °C overnight.

### MERFISH experiments

Tissue sections were processed following Vizgen’s protocol. After the 70% ethanol was removed, the sample was incubated in the Sample Prep Wash Buffer (PN20300001) for 1 min and then incubated in the Formamide Wash Buffer (PN20300002) at 37 °C for 30 min. After the Formamide Wash Buffer was removed, the sample was incubated in MERSCOPE Gene Panel Mix at 37 °C for 42 h. The 1,000-target panel was used for the three-day-old etiolated seedling (Col and *hls1*), stem apex and flower (cross section) samples, while the silique panel was used for the flower (longitudinal) and silique samples. After the probe hybridization, the sample was washed twice with the Formamide Wash Buffer at 47 °C for 30 min and once with the Sample Prep Wash Buffer at room temperature for 2 min. After the washing, the sample was embedded in hydrogel by incubating it in the Gel Embedding Solution (Gel Embedding Premix (PN20300004), 10% w/v ammonium persulfate solution and *N*,*N*,*N*′,*N*′-tetramethylethylenediamine) at room temperature for 1.5 h. The sample was then cleared by first incubating it in the Digestion Mix (Digestion Premix (PN 20300005) and 1:40 RNase inhibitor) at room temperature for 2 h, followed by the incubation in the Clearing Solution (Clearing Premix (PN 20300003) and Proteinase K) at 47 °C for 24 h and then at 37°C for 24 h. The cleared sample was washed twice with the Sample Prep Wash Buffer and stained with DAPI and PolyT Staining Reagent at room temperature for 15 min and then washed with the Formamide Wash Buffer at room temperature for 10 min and rinsed with the Sample Prep Wash Buffer. The sample was imaged with the MERSCOPE Instrument, and detected transcripts were decoded on the MERSCOPE Instrument using a Codebook generated by Vizgen. Transcripts were visualized on Vizgen’s Vizualizer.

### Curio Seeker spatial transcriptomics

Germinating seeds (1.25 days) were grown as previously described and were subsequently submerged in OCT in a cryomold (Sakura) and flash-frozen in a liquid N_2_ cooled isopentane bath. Cryosectioning of the 1.25-day-old germinating seeds was performed as described above. Following sectioning, the frozen section was arranged onto the centre of the Curio Seeker Spatial Mapping Kit (Curio Bioscience) and gently melted and stored at −80 °C until further processing according to the manufacturer’s specifications. Briefly, the frozen tile was gently thawed to room temperature and submerged in Hybridization Reaction Mix for 15 min, followed by sequential transfers to wash buffer and RT Reaction Mix at room temperature. Reverse transcription was performed via incubation at 52 °C for 30 min followed by tissue digestion with the addition of Tissue Clearing Reaction Mix and incubation at 37 °C for 30 min. Following tissue digestion, the beads were dissociated from the tile by the addition of Bead Wash Buffer and pipetting. The released beads were transferred to a new tube, pelleted via centrifugation and washed twice in Bead Wash Buffer. Following the second wash, the beads were incubated at 95 °C for 5 min and then pelleted and immediately resuspended Second Strand Mix and incubated at 37 °C for 60 min. Following incubation, Bead Wash Buffer was added, and the beads were washed three times with Bead Wash Buffer. After the third wash, the beads were resuspended in and amplified in cDNA Amplification Mix via PCR. The amplified cDNA was then purified and quantified, followed by tagmentation and clean-up. The libraries were then sequenced on the NovaSeq 6000.

### Curio spatial transcriptomics analysis

Spot demultiplexing was performed according to the manufacturer’s specifications using the Seeker Bioinformatic Pipeline (Curio Bioscience). The resulting spot-by-gene matrix was used for downstream analysis with Seurat (v.4.1.0). Empty and low-quality spots with fewer than 125 genes detected or 125 UMIs were removed. The filtered dataset was normalized with SCTransform with the variable features calculated with a residual variance cut-off of 1. Spatially aware clustering was performed on a SpatialExperiment (v.1.12.0) object using Banksy (v.0.99.7)^87^ with the parameters lambda = 0.2 and k_geom = 10, and the resulting matrix was used for PCA followed by UMAP dimensionality reduction with 20 PCs, followed by nearest neighbour identification and clustering. Identification of cluster markers was performed as described previously.

### Cell segmentation of the flower MERFISH datasets

Cell segmentation was performed using Baysor^88^ (v.0.7.1) on the processed transcript coordinate matrix with the following parameters: s, 6; x, global_x; y, global_y; g, gene; m, 20; p; n-clusters, 10; scale-std, 90%; count-matrix-format, ‘tsv’.

### Spatial single-cell analysis of the flower MERFISH datasets

Cell segmentation boundaries, centroids, molecule positions and cell-by-gene count matrices were analysed with Seurat v.5.2.1. All datasets were analysed individually and were normalized with SCTransform for all targets detected in each spatial dataset with the parameter clip. range = c(−10, 10). PCs were calculated and used for UMAP dimensionality reduction with 30 PCs, followed by nearest neighbour identification and clustering. Identification of cluster markers was performed as described previously.

### Silique length measurement

Plants were grown as previously described until all flowers had dehisced. All fully elongated siliques from the primary stem (*n* > 9) were harvested from all individual plants. The length of the silique was measured using digital calipers from the tip to the base of the silique and was rounded to the nearest 0.1 mm. For Col-0 and the *miox1–1* mutant, siliques from a total of eight individuals across three biological replicates were measured. For the *miox1–2* and *miox1–3* mutants, silique measurements were taken from two individuals from two biological replicates. For the statistical analysis, an unpaired Wilcoxon rank sum test was performed for each *miox1* mutant compared with Col-0, and the figures were generated using ggplot2 (v.3.3.6)^89^ and ggpubr (v.0.6.0)^90^.

### Statistics and reproducibility

For the spatial transcriptomic experiments, similar results were observed across individual samples and across independent experiments. For the germinating seed dataset, *n* > 10 independent seeds were profiled simultaneously in one experiment. For the Col-0 seedling dataset, *n* = 8 independent seedlings were simultaneously profiled in one experiment. Technical replication of adjacent tissue sections was performed for the same seedlings. Identical sampling of *n* = 8 independent seedlings was performed for the *hls1* seedling dataset. For the stem dataset, *n* = 1 stem was profiled. For the flower dataset, *n* = 3 independent flowers were profiled (*n* = 2 in transverse orientation, *n* = 1 in longitudinal orientation) across two experiments. For the silique dataset, *n* = 3 independent siliques were profiled in one experiment.

## Extended Data

## Supplementary Material

Sup Table 6

Sup Table 5

Sup Table 4

Sup Table 3

Sup Table 2

Sup Table 1

Supplementary Figures

**Supplementary information** The online version contains supplementary material available at https://doi.org/10.1038/s41477-025-02072-z.

## Data Availability

All datasets can be accessed through our web tool at http://arabidopsisdevatlas.salk.edu/. The raw and processed sequencing datasets are available via GEO (accession number GSE226097). The processed spatial datasets are available for download at our web resource.
