# Supplementary material for: A single-cell, spatial transcriptomic atlas of the Arabidopsis life cycle
Source: Nat Plants. 2025 Aug 19;11(9):1960–75. doi: 10.1038/s41477-025-02072-z (PMC12416547; doi:10.1038/s41477-025-02072-z)
Supplement: Supplementary file 2 — Reporting Summary [file 41477_2025_2072_MOESM2_ESM.pdf]

nature portfolio

Corresponding author(s): Joseph R. Ecker

Last updated by author(s): Jul 3, 2025

Reporting Summary

Nature Portfolio wishes to improve the reproducibility of the work that we publish. This form provides structure for consistency and transparency in reporting. For further information on Nature Portfolio policies, see our Editorial Policies and the Editorial Policy Checklist.

Statistics

For all statistical analyses, confirm that the following items are present in the figure legend, table legend, main text, or Methods section.

n/a	Confirmed	
☐	☒	The exact sample size (*n*) for each experimental group/condition, given as a discrete number and unit of measurement
☐	☒	A statement on whether measurements were taken from distinct samples or whether the same sample was measured repeatedly
☐	☒	The statistical test(s) used AND whether they are one- or two-sided<br>*Only common tests should be described solely by name; describe more complex techniques in the Methods section.*
☒	☐	A description of all covariates tested
☒	☐	A description of any assumptions or corrections, such as tests of normality and adjustment for multiple comparisons
☐	☒	A full description of the statistical parameters including central tendency (e.g. means) or other basic estimates (e.g. regression coefficient) AND variation (e.g. standard deviation) or associated estimates of uncertainty (e.g. confidence intervals)
☐	☒	For null hypothesis testing, the test statistic (e.g. *F*, *t*, *r*) with confidence intervals, effect sizes, degrees of freedom and *P* value noted<br>*Give P values as exact values whenever suitable.*
☒	☐	For Bayesian analysis, information on the choice of priors and Markov chain Monte Carlo settings
☒	☐	For hierarchical and complex designs, identification of the appropriate level for tests and full reporting of outcomes
☒	☐	Estimates of effect sizes (e.g. Cohen's *d*, Pearson's *r*), indicating how they were calculated

Our web collection on statistics for biologists contains articles on many of the points above.

Software and code

Policy information about availability of computer code

Data collection	Illumina NovaSeq 6000, Vizgen MERSCOPE, Curio Seeker
Data analysis	R (4.1.3), Seurat (4.1.0), Seurat (5.2.1), ggplot2 (3.3.6), dplyr (1.0.9), harmony (0.1.0), ggpubr (0.6.0), Banksy (0.99.7), SpatialExperiment (1.12.0), ComplexUpset (1.3.3), clusterProfiler (4.2.2), Baysor (0.7.1), CellRanger (7.0.0)

For manuscripts utilizing custom algorithms or software that are central to the research but not yet described in published literature, software must be made available to editors and reviewers. We strongly encourage code deposition in a community repository (e.g. GitHub). See the Nature Portfolio guidelines for submitting code & software for further information.

Data

Policy information about availability of data

All manuscripts must include a data availability statement. This statement should provide the following information, where applicable:

- Accession codes, unique identifiers, or web links for publicly available datasets
- A description of any restrictions on data availability
- For clinical datasets or third party data, please ensure that the statement adheres to our policy

All datasets can be accessed through our web tool at: arabidopsisdevatlas.salk.edu. Raw and processed sequencing datasets are available at GEO (accession number GSE226097). Processed spatial datasets are available for download at our web resource (arabidopsisdevatlas.salk.edu). Arabidopsis GO terms were compiled from the TAIR 220801 release.

Research involving human participants, their data, or biological material

Policy information about studies with human participants or human data. See also policy information about sex, gender (identity/presentation), and sexual orientation and race, ethnicity and racism.

Reporting on sex and gender	N/A
Reporting on race, ethnicity, or other socially relevant groupings	N/A
Population characteristics	N/A
Recruitment	N/A
Ethics oversight	N/A

Note that full information on the approval of the study protocol must also be provided in the manuscript.

Field-specific reporting

Please select the one below that is the best fit for your research. If you are not sure, read the appropriate sections before making your selection.

☒ Life sciences ☐ Behavioural & social sciences ☐ Ecological, evolutionary & environmental sciences

For a reference copy of the document with all sections, see nature.com/documents/nr-reporting-summary-flat.pdf

Life sciences study design

All studies must disclose on these points even when the disclosure is negative.

Sample size	No sample size caluclation was performed. For the single-nucleus experiments, the number of nuclei that were sequenced was determined based on the ability to identify and annotate known cell types within each tissue sampled. For the spatial transcriptome experiments, for the seed (n > 10), seedling (n > 8), flower (n = 3), and silique tissues (n = 3), samples of several independent plants were assayed simultaneously in single or multiple experiments which enabled the consistent observation of expression patterns in multiple individual plants. For the stem sample, one sample was profiled where we found expression patterns that were highly consistent with known expression patterns.<br><br>For the silique measurement experiments, all fully elongated siliques from the primary stem (n > 9) were collected from individual plants. For the Col-0 and miox1-1 allele, siliques were measured from n > 8 individual plants across three biological replicates to ensure reproducibility of results. For the miox1-2 and miox1-3 alleles, siliques from two individual plants from two biological replicates were measured to ensure consistent phenotypes are observed in other indepent T-DNA alleles of miox1.
Data exclusions	No data was excluded in the analysis of this manuscript.
Replication	For each single nuclei datasets, tissue from n > 18 individual plants were harvested and pooled for nuclei extraction. For each replicate, the total nuclei were pooled for downstream purification and then evenly split into at least two individual 10X 3' Gene Expression reactions. All attempts at replication were successful.<br><br>For silique length phenotyping experiments, all fully elongated siliques (n > 9) were harvested from the primary stem of one plant. For the primary experiment, this was performed for greater than eight individual plants over three biological replicates for both Col and the miox1-1 allele. For the miox1-2 and miox1-3 alleles, the phenotyping was performed for two individuals from two biological replicates. All attempts at replication were successful.
Randomization	For seed and seedling samples, all plants were grown seeded and grown in petri dishes which were randomly placed within growth chambers. For all remaining tissues, plants were grown in pots that were placed randomly into larger flats for uniform watering. From the randomized flats, plants were randomly selected for harvest for experimental groups.
Blinding	Blinding was not performed in our study. Mutants with visible phenotypes were also investigated in our study which could be identified.

Reporting for specific materials, systems and methods

We require information from authors about some types of materials, experimental systems and methods used in many studies. Here, indicate whether each material, system or method listed is relevant to your study. If you are not sure if a list item applies to your research, read the appropriate section before selecting a response.

Materials & experimental systems

n/a	Involved in the study	
☒	☐	Antibodies
☒	☐	Eukaryotic cell lines
☒	☐	Palaeontology and archaeology
☒	☐	Animals and other organisms
☒	☐	Clinical data
☒	☐	Dual use research of concern
☐	☒	Plants

Methods

n/a	Involved in the study	
☒	☐	ChIP-seq
☒	☐	Flow cytometry
☒	☐	MRI-based neuroimaging

Dual use research of concern

Policy information about dual use research of concern

Hazards

Could the accidental, deliberate or reckless misuse of agents or technologies generated in the work, or the application of information presented in the manuscript, pose a threat to:

No	Yes	
☒	☐	Public health
☒	☐	National security
☒	☐	Crops and/or livestock
☒	☐	Ecosystems
☒	☐	Any other significant area

Experiments of concern

Does the work involve any of these experiments of concern:

No	Yes	
☒	☐	Demonstrate how to render a vaccine ineffective
☒	☐	Confer resistance to therapeutically useful antibiotics or antiviral agents
☒	☐	Enhance the virulence of a pathogen or render a nonpathogen virulent
☒	☐	Increase transmissibility of a pathogen
☒	☐	Alter the host range of a pathogen
☒	☐	Enable evasion of diagnostic/detection modalities
☒	☐	Enable the weaponization of a biological agent or toxin
☒	☐	Any other potentially harmful combination of experiments and agents

Plants

Seed stocks	Col-0 CS60000 seed was used for all experiments. T-DNA alleles for phenotypic analysis were collected from the SALK and SALKseq collections deposited to ABRC. The following T-DNA alleles are presented in the manuscript. miox1-1 (CS326247), miox1-2 (CS850198), miox1-3 (CS337844), kinesin 7.3-1 (SALK_105057C), kinesin 7.3-2 (SALK_038138C), atfp3 (SALK_038143C)
Novel plant genotypes	No novel genotypes were generated for this study.
Authentication	All T-DNA lines aside from the miox1-2 allele are single insertion lines and aside from the miox1-2 allele, confirmed homozygous for the T-DNA insertion. For the segregating miox1-2 allele (CS850198), homozygous plants were identified by PCR with primers designed by http://signal. salk.edu/tdnaprimer.2.html
