# Supplementary material for: A single-cell, spatial transcriptomic atlas of the Arabidopsis life cycle
Source: Nat Plants. Author manuscript; Available in PMC 2025 Sep 9. (PMC12416547; doi:10.1038/s41477-025-02072-z)
Supplement: Supplementary file 1 [file NIHMS2106499-supplement-Supplementary_Figures.pdf]

nature plants

Supplementary information https://doi.org/10.1038/s41477-025-02072-z

A single-cell, spatial transcriptomic atlas of the *Arabidopsis* life cycle

In the format provided by the authors and unedited

The PDF file includes:

Supplementary Figs. 1 to 19
Supplementary Fig. 1. Transcriptional complexity of nuclei among all datasets
Supplementary Fig. 2. Transcriptional complexity of nuclei among all datasets
Supplementary Fig. 3. Expression of senescence regulated transcription factors in 21d- and 30d-old-rosettes
Supplementary Fig. 4. Cluster annotation of cell types within individual datasets
Supplementary Fig. 5. Subcluster diversity throughout the Arabidopsis lifecycle
Supplementary Fig. 6. GO term enrichment of subcluster markers of the dividing cell cluster in siliques
Supplementary Fig. 7. Expression of polarity regulators and transcripts with novel polar expression patterns within all datasets
Supplementary Fig. 8. Spatial detection of transcripts in adaxial regions of cotyledons
Supplementary Fig. 9. Co-expression of known polarity regulators and novel markers with polar spatial expression patterns
Supplementary Fig. 10. Co-expression of TT4 and mesophyll and epidermal cell type markers
Supplementary Fig. 11. Expression diversity of flavonoid biosynthesis enzymes across organs
Supplementary Fig. 12. Modification of clustering parameters consistently reveals cluster specific expression of TT4
Supplementary Fig. 13. Modification of clustering parameters consistently reveals cluster specific enrichment of flavonoid biosynthesis enzymes
Supplementary Fig. 14. Cross-tissue cell type analysis of ten cell type populations
Supplementary Fig. 15. Investigation and functional validation of genes with cell type and developmental specificity
Supplementary Fig. 16. GO term enrichment of markers of cortex cellular states within the apical hook
Supplementary Fig. 17. Integration of the rosette single-nuclei datasets with a protoplast dataset
Supplementary Fig. 18. Spatial single-cell analysis of the longitudinal flower MERFISH dataset
Supplementary Fig. 19. Spatial single-cell analysis of a MERFISH flower MERFISH cross section dataset

Other Supplementary Materials for this manuscript include the following:

Extended Data Figs. 1 to 10
Supplementary Tables 1 to 6

Supplementary Figures

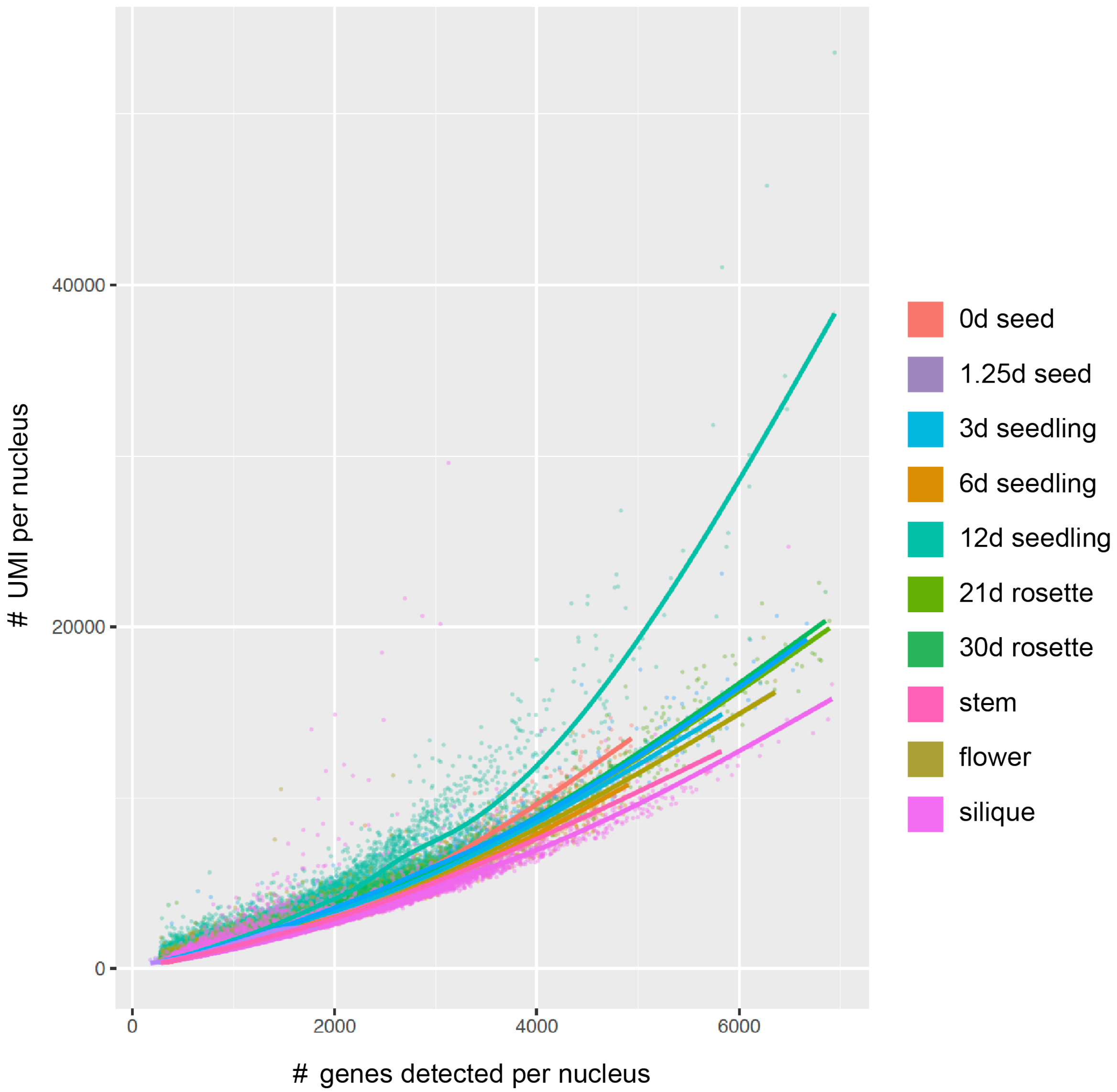

Supplementary Fig. 1. Transcriptional complexity of nuclei among all datasets

The number of genes detected and the total # of UMI identified within nuclei of all datasets. Dots are colored according to dataset of origin. The line depicts the mean relationship between genes detected and UMI per nucleus.

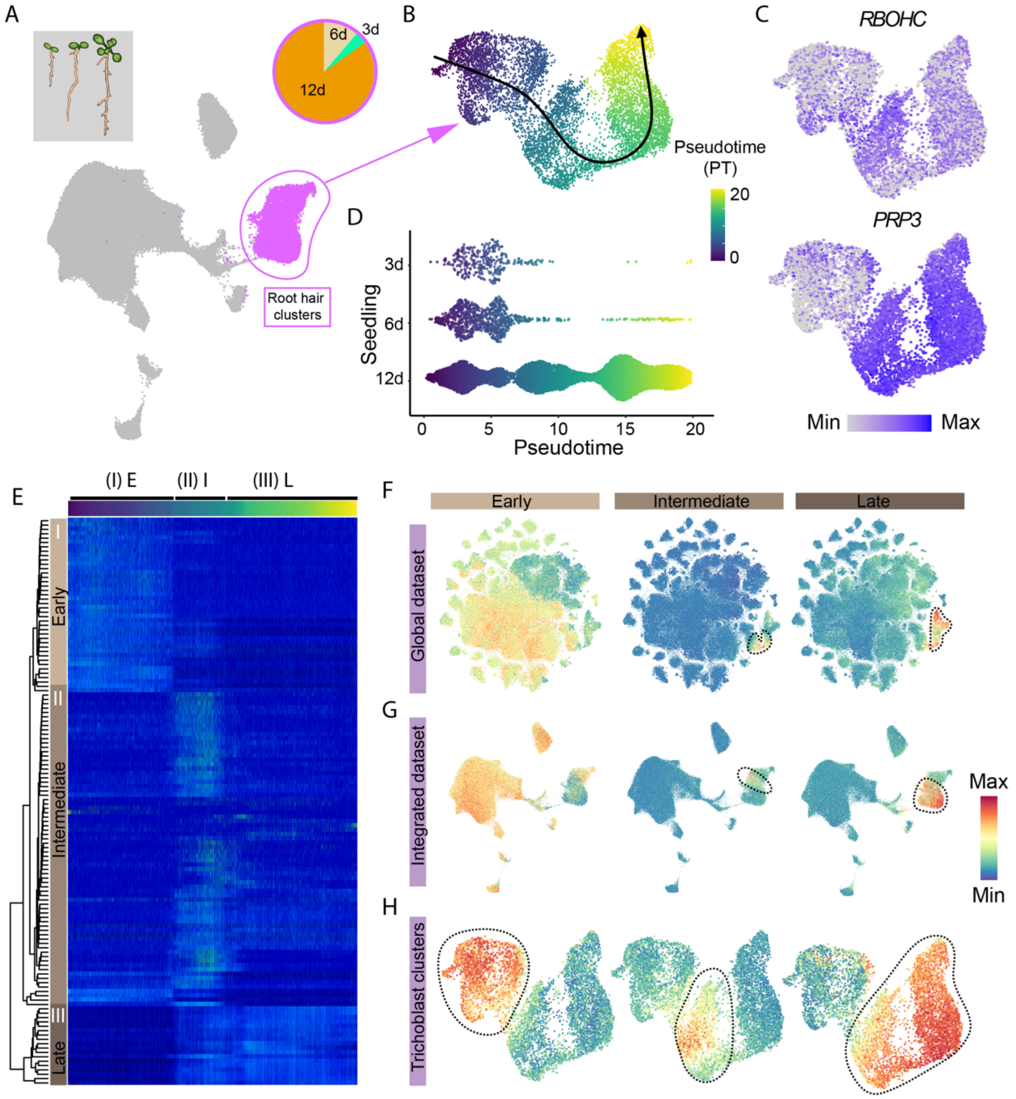

Supplementary Fig. 2. Transcriptional programs of root hair development across the Arabidopsis lifecycle

a, Integrated clustering of the 3d, 6d, and 12d seedling datasets. Root hair (trichoblast) clusters are circled, and the distribution of nuclei within the trichoblast clusters among developmental time points is depicted. b, Re-clustering and pseudotime trajectory of annotated root hair clusters. c, Expression of known marker genes of root hair development (*ROBHC* [*AT5G51060*] and *PRP3* [*AT3G62680*]) d, Distribution of root hair cells from 3d-, 6d-, and 12d-old seedlings across pseudotime. e, Heatmap of 170 genes differentially expressed along pseudotime of root hair cells. Three major classes of genes corresponding to (I) E=early, (II) I=intermediate, and (III) L=late pseudotime, are depicted. f to h, Expression of the three gene modules identified from early, intermediate, and late pseudotime of root hair development in the (f) global, (g) integrated seedling, and (h) root hair re-clustering.

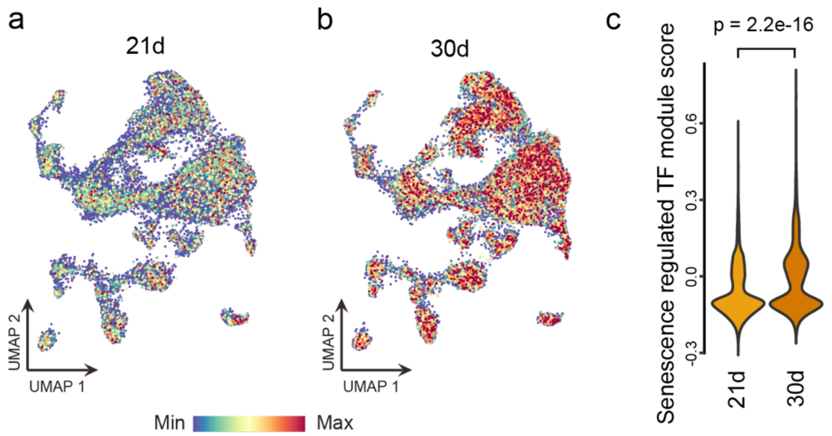

Supplementary Fig. 3. Expression of senescence regulated transcription factors in 21d- and 30d-old-rosettes

a to c, Expression of senescence-regulated transcription factors in the 21d- and 30d-old rosette datasets. (a and b) UMAP of the integrated rosette dataset with the 21d- (a) and 30d-old (b) rosette datasets depicted separately. Nuclei are colored by the senescence TF expression score. c, Violin plot showing the averaged expression of 12 senescence transcription factors[8] within all nuclei of the 21d- (n = 26,864) and 30d-old (n = 37,876) rosette datasets. Significance result of a two-sided Welch's t-test is depicted.

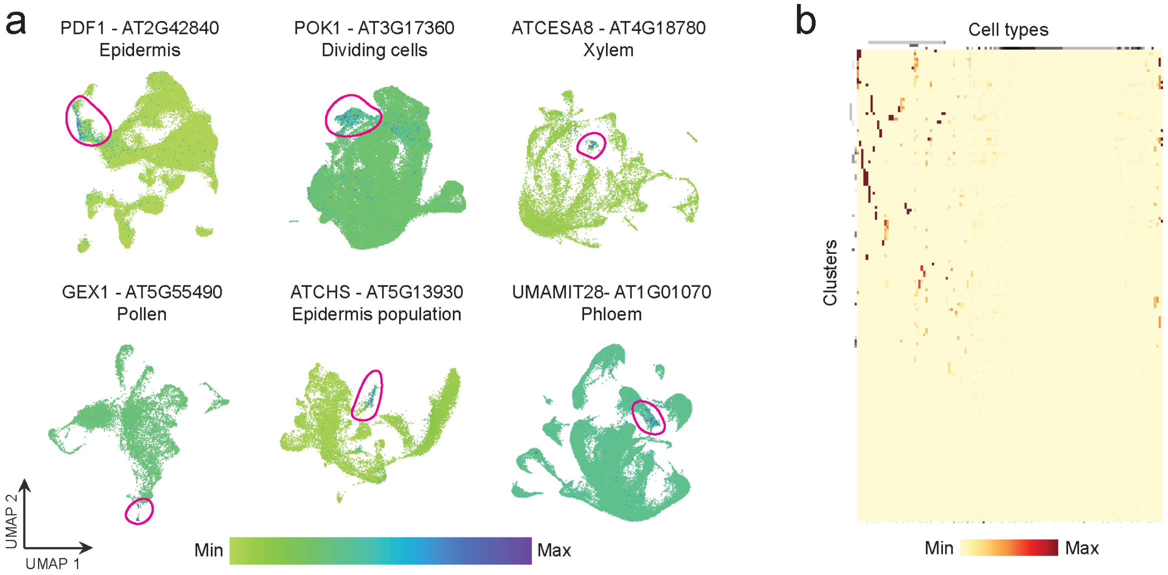

Supplementary Fig. 4. Cluster annotation of cell types within individual datasets
a, Expression patterns of known cell type-specific marker genes as visualized in our Arabidopsis developmental atlas browser. Regions of enriched expression are circled. b, Marker gene enrichment score for each cell type/state in each major cluster.

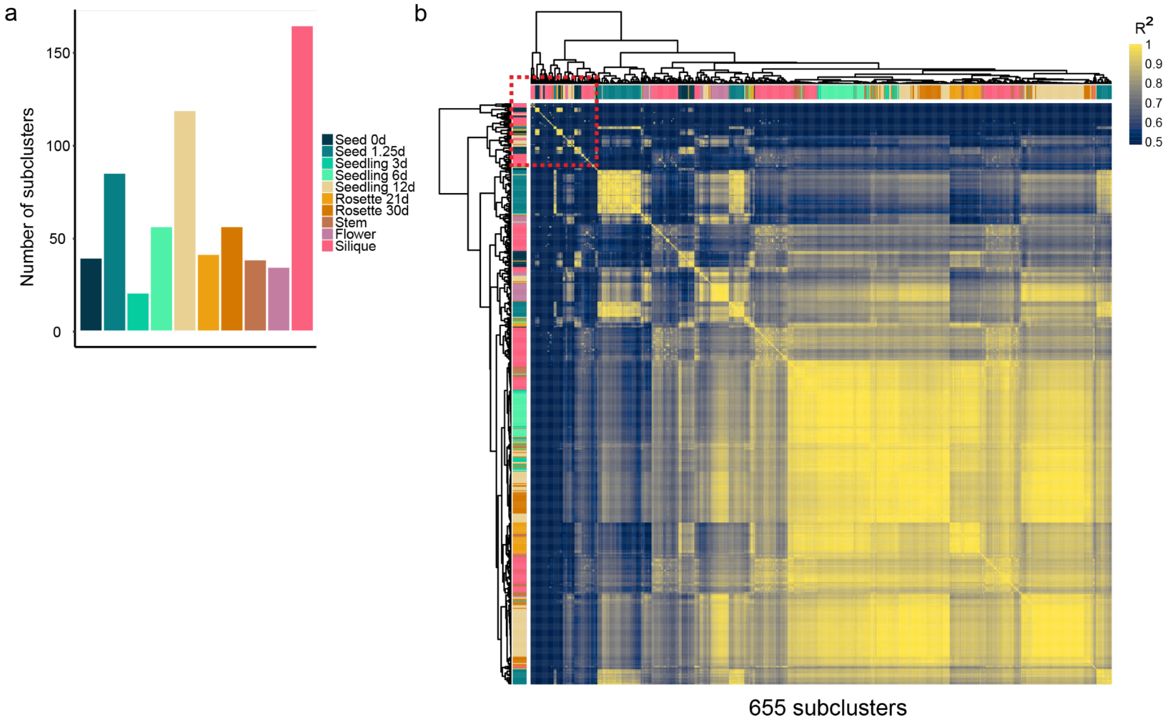

Supplementary Fig. 5. Subcluster diversity throughout the Arabidopsis lifecycle

a, The total number of subclusters identified within each dataset. b, Correlation heatmap of pseudobulk transcriptomes across 655 subclusters. The top color bars indicate the sample of origin. The highlighted region depicts expression diversity within subclusters of diverse samples.

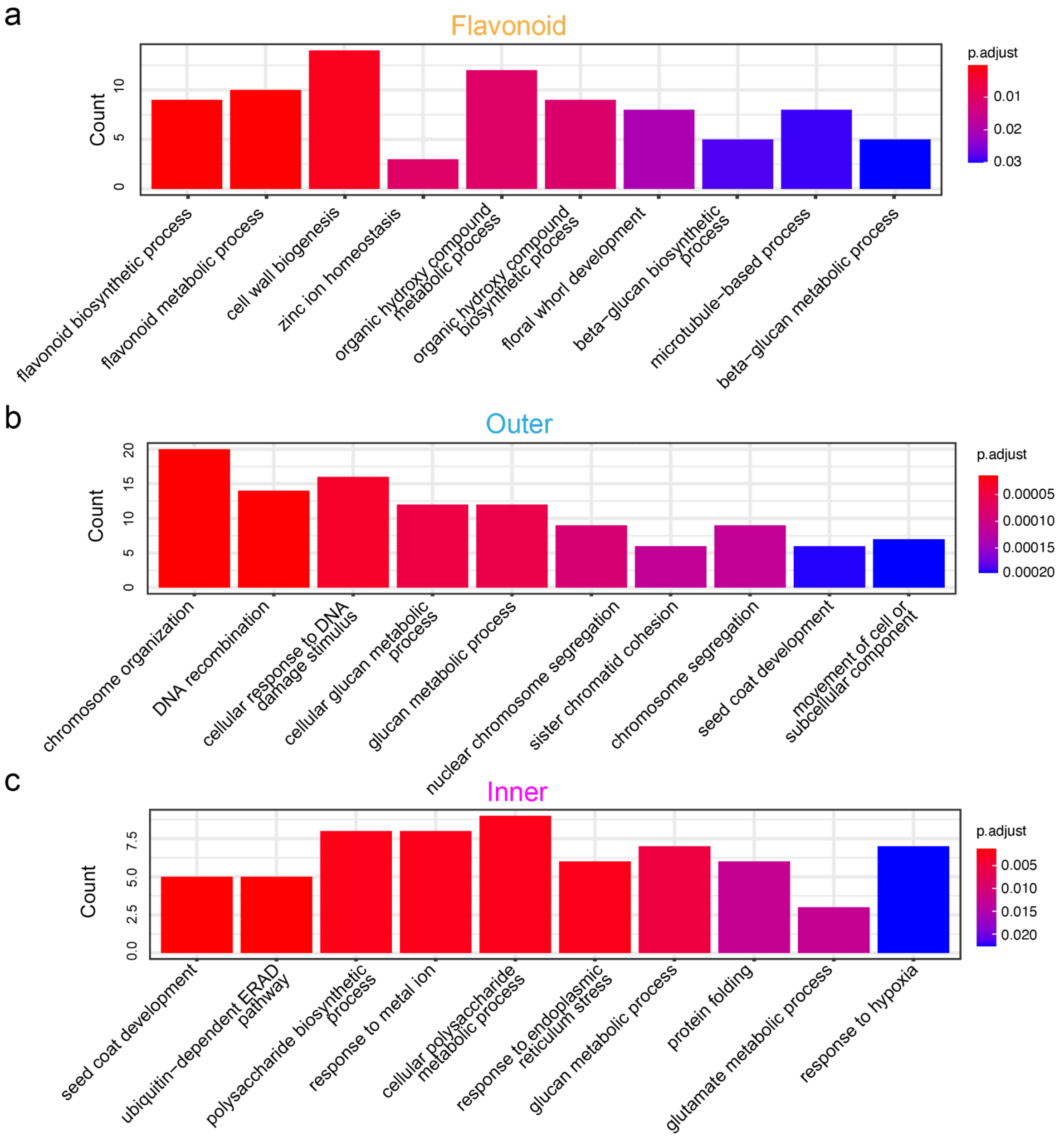

Supplementary Fig. 6. GO term enrichment of subcluster markers of the dividing cell cluster in siliques

a-c, Top 10 enriched GO terms for subcluster markers corresponding to the outer integument (blue), inner integument (pink), and flavonoid biosynthesis (orange) subclusters of the proliferating cells cluster (cluster 0) of the silique dataset. Adjusted p-values were calculated using a one-sided hypergeometric test followed by Benjamini-Hochberg correction.

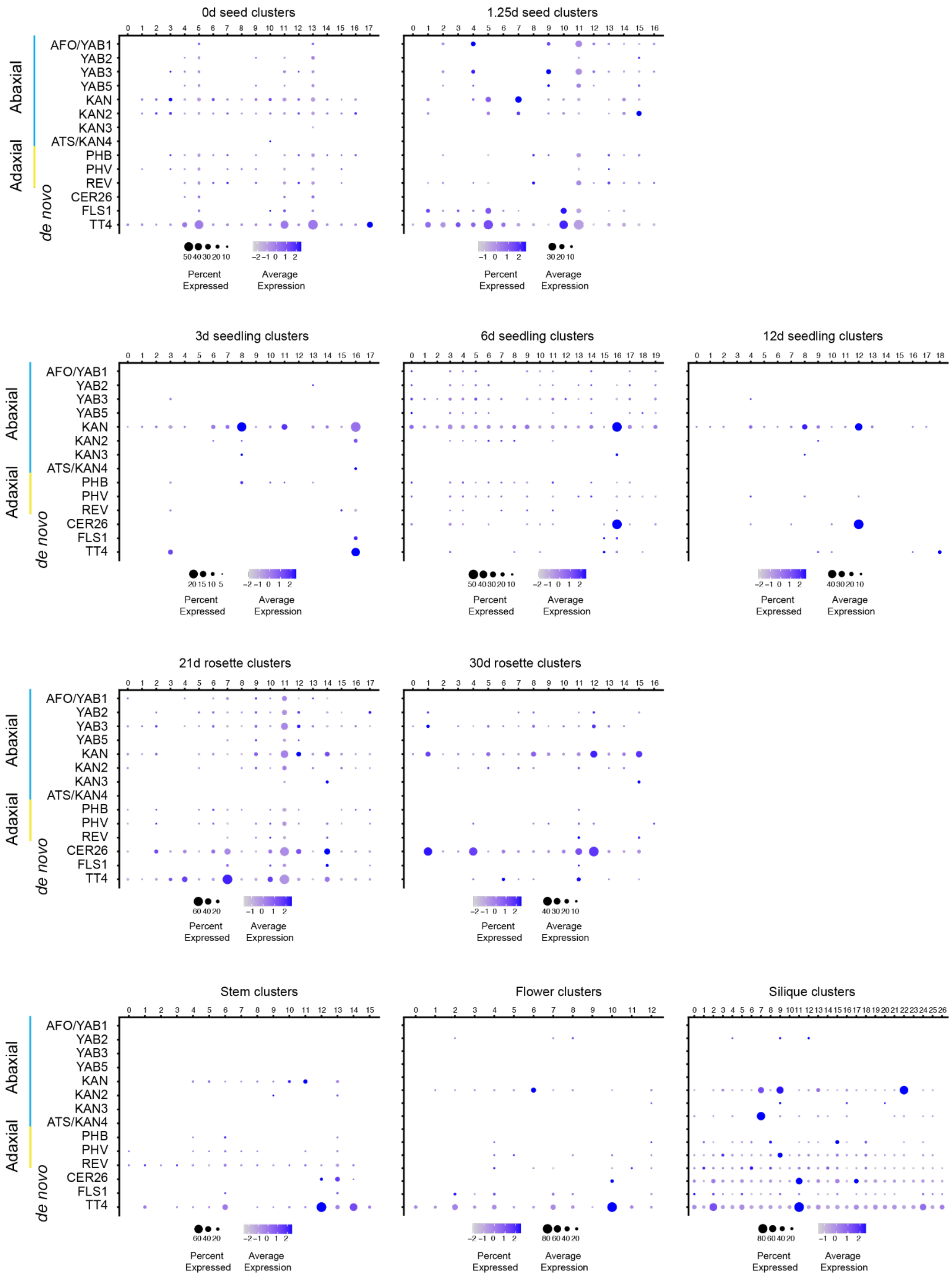

Supplementary Fig. 7. Expression of polarity regulators and transcripts with novel polar expression patterns within all datasets

Expression of regulators of abaxial and adaxial cell fate and transcripts with novel polar spatial expression patterns within clusters of all datasets.

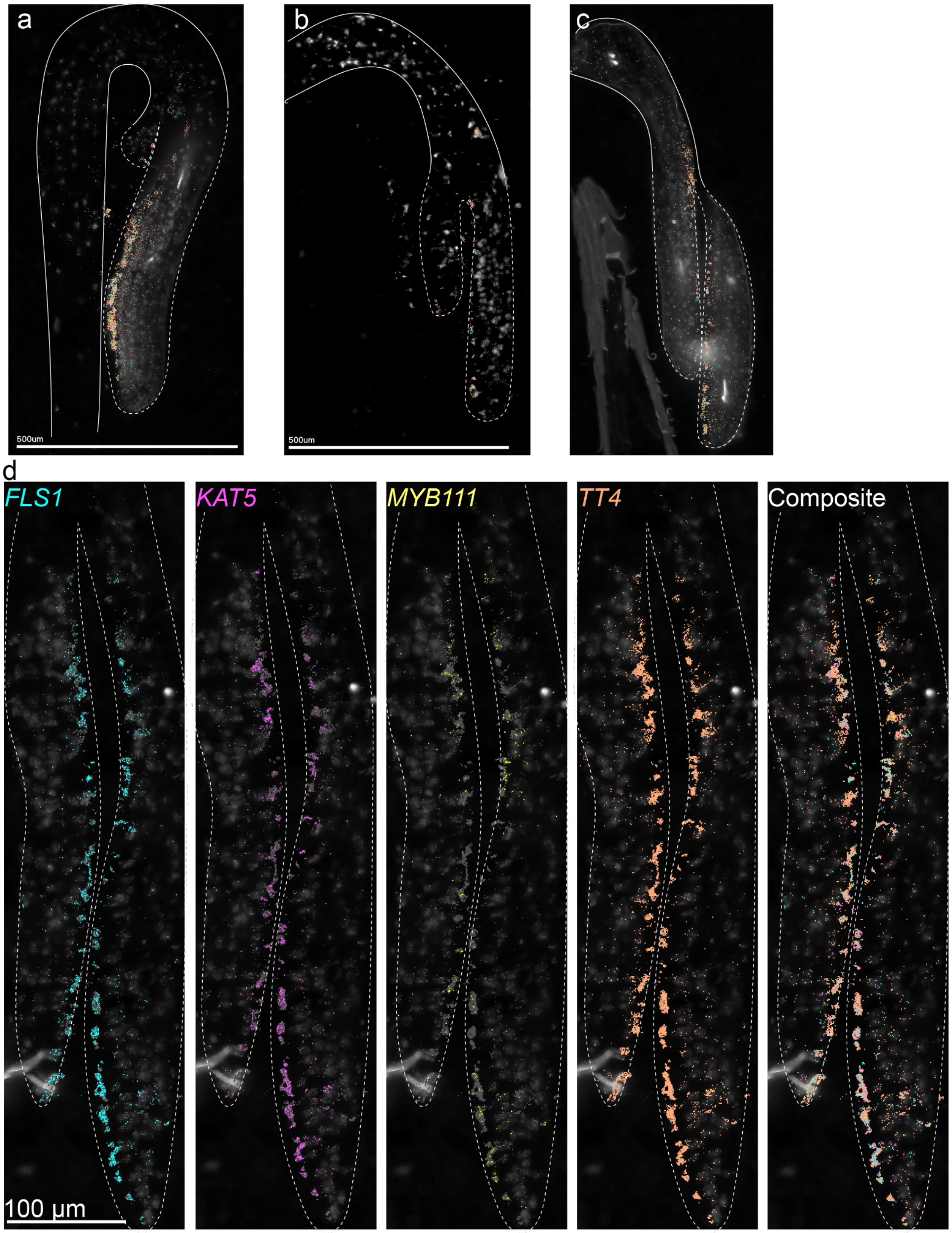

Supplementary Fig. 8. Spatial detection of transcripts in adaxial regions of cotyledons

a-d, Spatial detection of transcripts localized to adaxial cells of cotyledons in 3-day-old seedlings. Individual mRNA molecules of *FLS1*, *KAT5*, *MYB111*, and *TT4* are depicted as colored in (d). Scale bar size is depicted. DAPI signal is colored white. Scale bar length is depicted. For the micrographs depicted in a-d, results were observed in five seedlings.

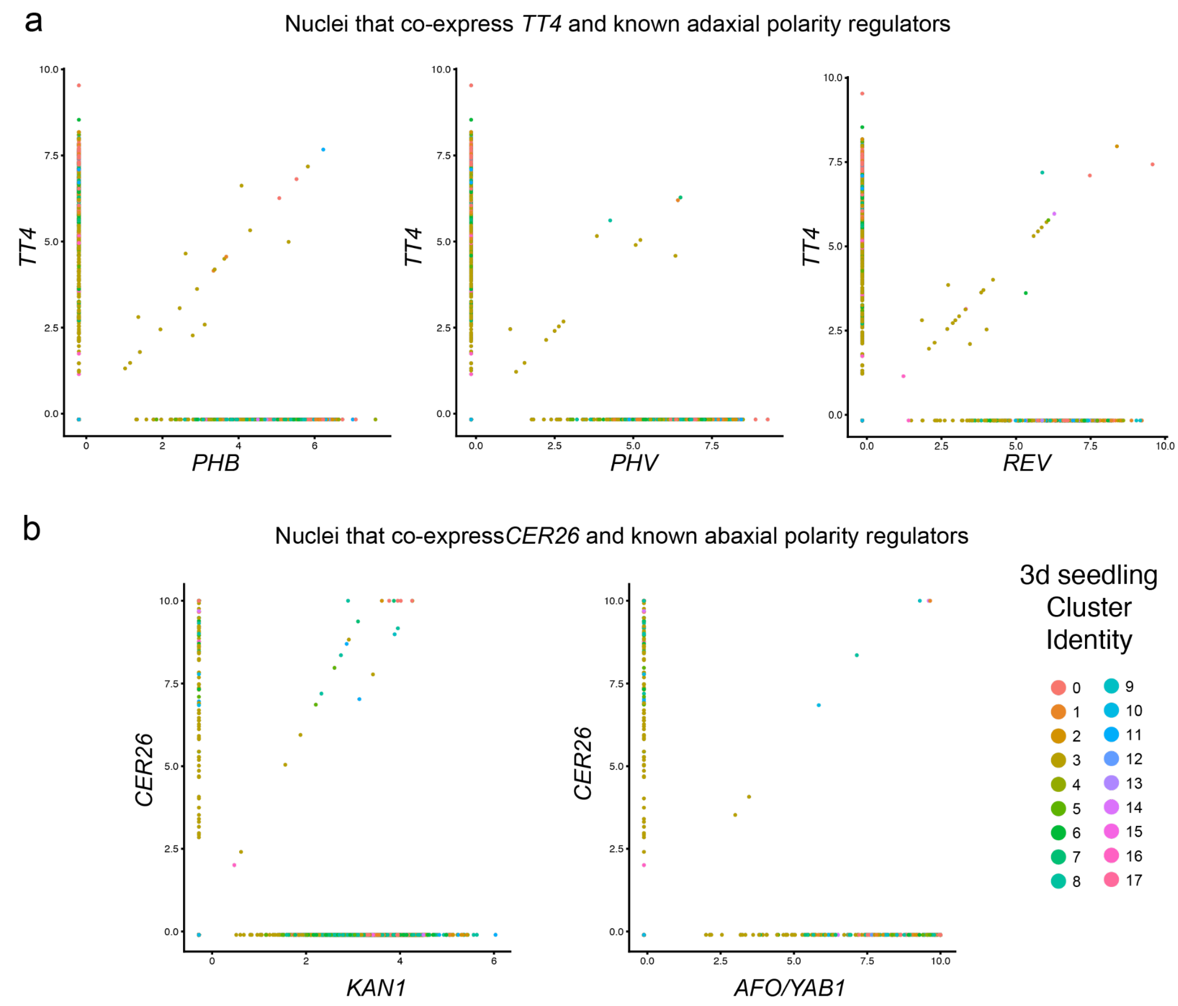

Supplementary Fig. 9. Co-expression of known polarity regulators and novel markers with polar spatial expression patterns

a, Co-expression level of *TT4* and the adaxial polarity regulators *PHB*, *PHV*, and *REV* within nuclei of the 3d-old-seedling dataset. Nuclei are colored by cluster. b, Co-expression level of *CER26* and the abaxial polarity regulators *KAN1* and *AFO/YAB1* within nuclei of the 3d-old-seedling dataset. Nuclei are colored by cluster.

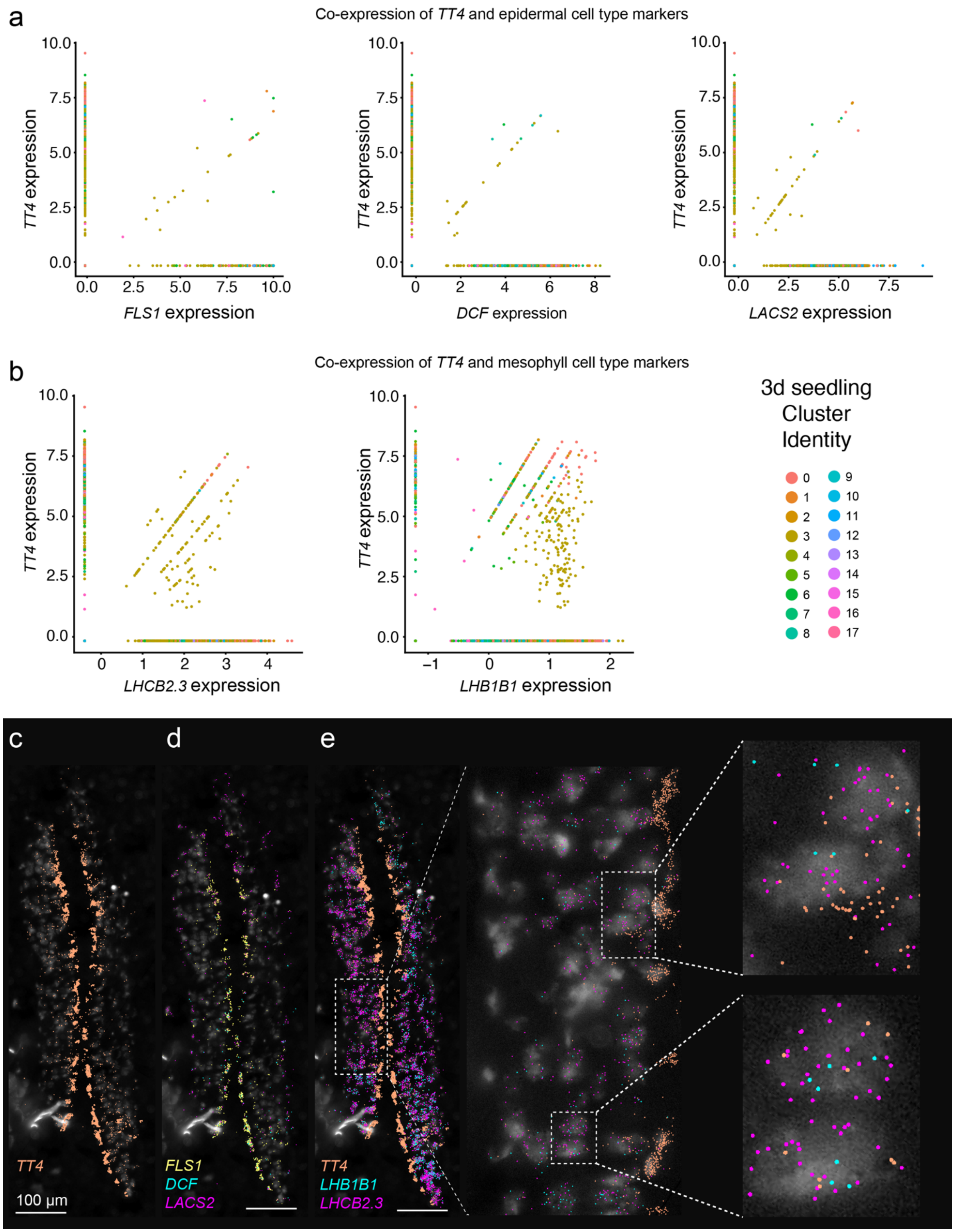

Supplementary Fig. 10. Co-expression of *TT4* and mesophyll and epidermal cell type markers
a, Co-expression level of *TT4* and the epidermal cell type markers *FLS1*, *DCF*, and *LACS2* within nuclei of the 3d-old-seedling dataset. Nuclei are colored by cluster. b, Co-expression level of *TT4* and the mesophyll

cell type markers *LHCB2.3* and *LHB1B1* within nuclei of the 3d-old-seedling dataset. Nuclei are colored by cluster. c to e, Spatial co-expression of *TT4* within epidermal and mesophyll cell types. (c) Spatial detection of *TT4* transcripts within adaxial cells of cotyledons. (d) Spatial detection of the epidermal cell type markers *FLS1, DCF,* and *LACS2* transcripts within the adaxial epidermis of cotyledons. (e) Spatial co-detection of *TT4* and the mesophyll cell type markers *LHB1B1* and *LHCB2.3* transcripts within cotyledon mesophyll cells. Magnified images of the highlighted ROI of the cotyledon cell layers (middle) and at single-cell resolution (right) are depicted. Individual mRNA transcripts are colored according to color legends. Scale bar size is depicted. DAPI signal is colored white. For the micrographs depicted in c-e, results were observed in five seedlings.

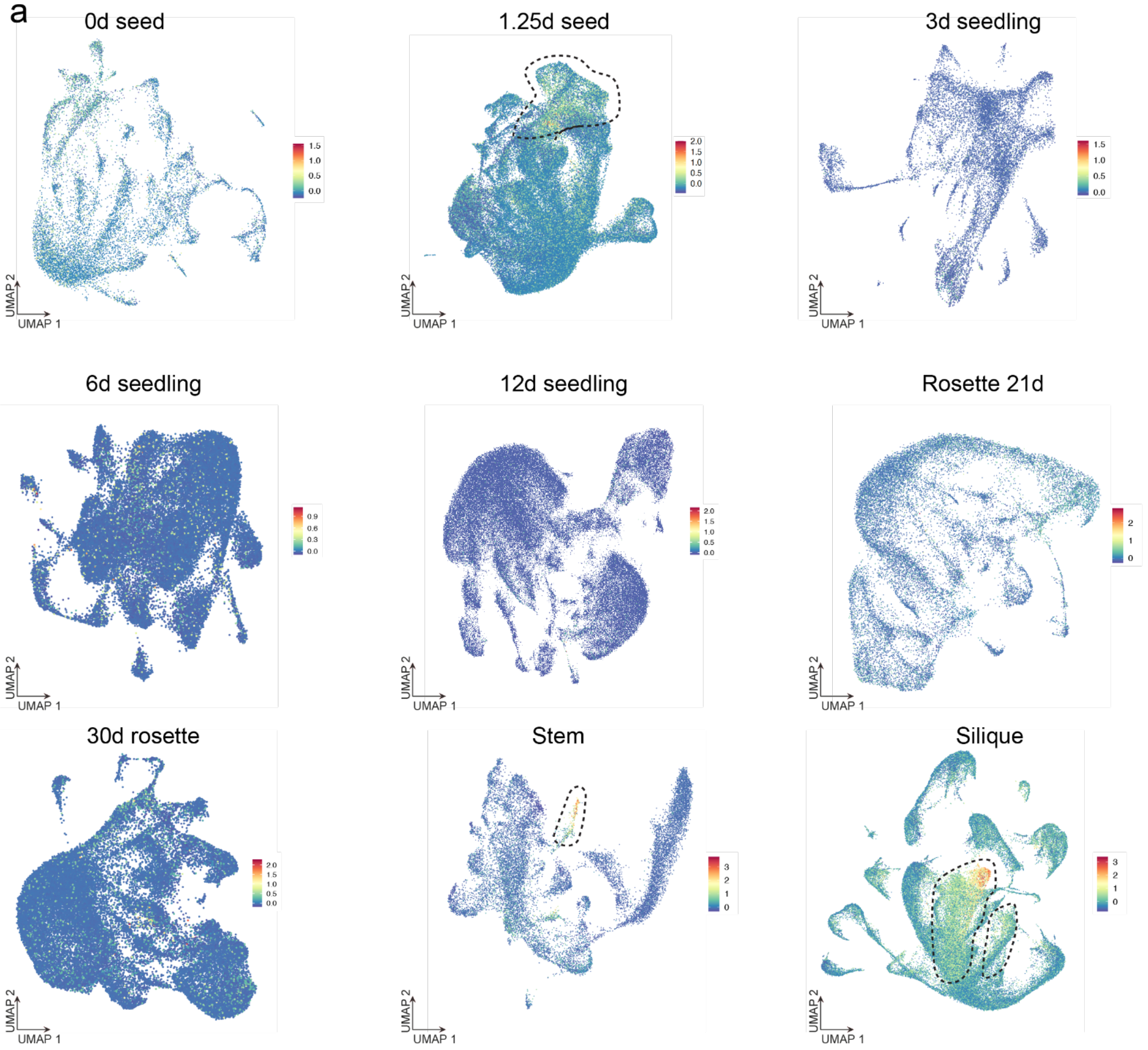

Supplementary Fig. 11. Expression diversity of flavonoid biosynthesis enzymes across datasets
a, Average expression of enzymes of the flavonoid biosynthesis pathway (*TT3-7*, *TT18*) within the remaining single-nucleus datasets. Clusters with enriched expression of flavonoid biosynthesis genes are circled.

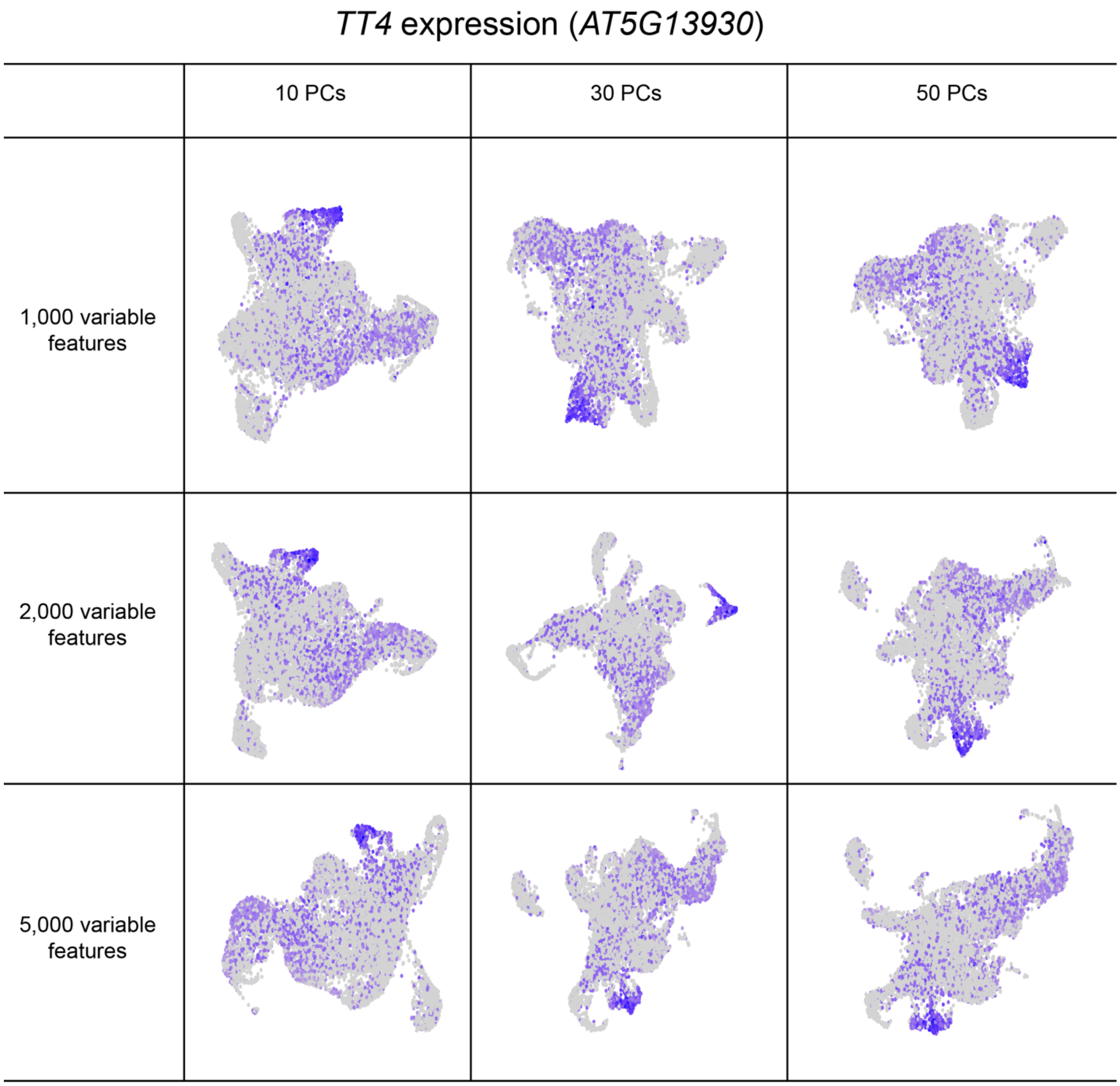

Supplementary Fig. 12. Modification of clustering parameters consistently reveals cluster specific expression of *TT4* in flowers

Expression of *TT4* within the flower dataset using various combinations of variable features (rows) and principal components (columns) for clustering. For all combinations of parameters, *TT4* expression is enriched within a single cluster.

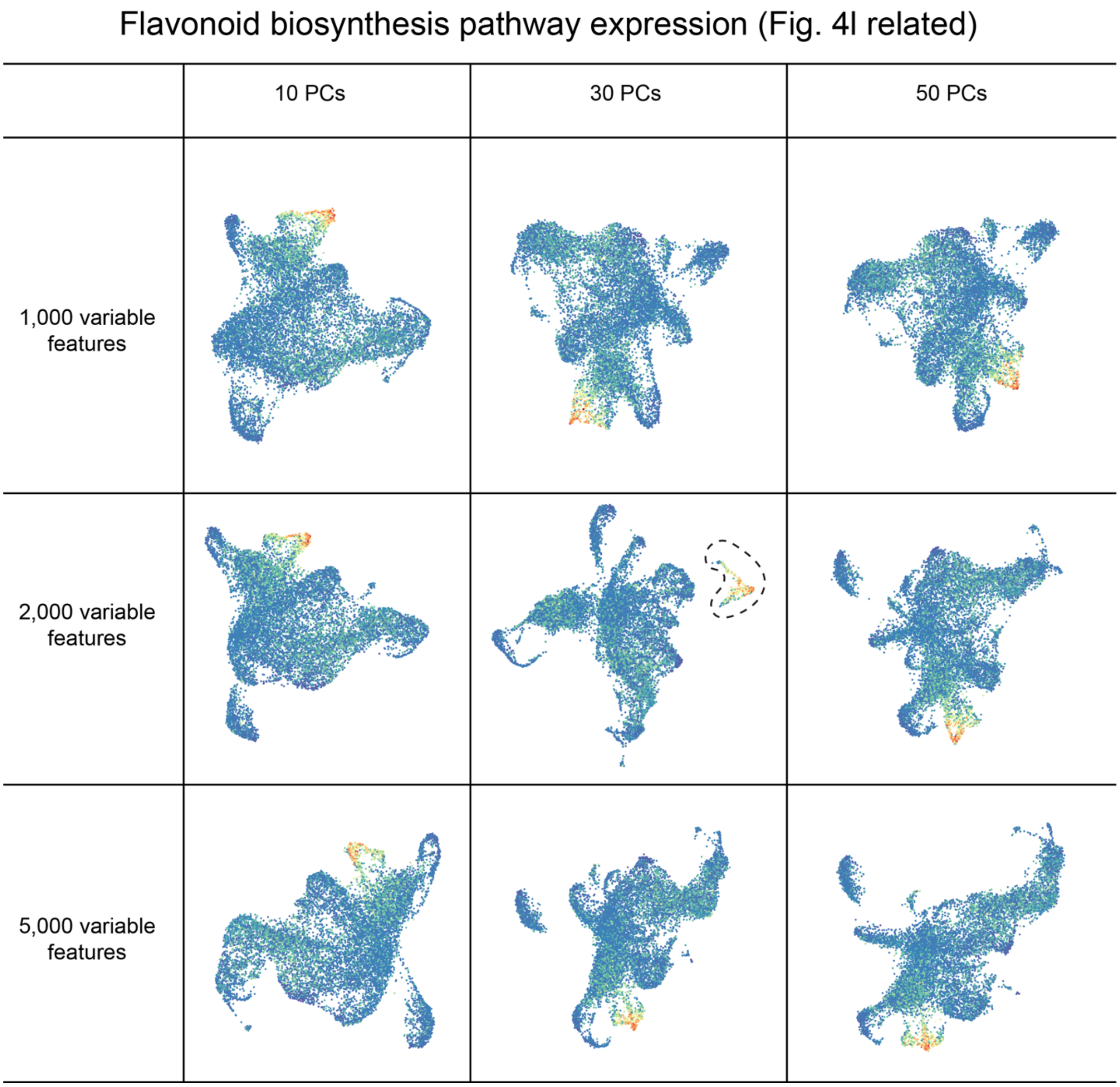

Supplementary Fig. 13. Modification of clustering parameters consistently reveals cluster specific enrichment of flavonoid biosynthesis enzymes in flowers
Expression of all flavonoid biosynthetic enzymes (*TT4, TT5, TT6, TT7, TT3, and TT18*) within the flower dataset using various combinations of variable features (rows) and principal components (columns) for clustering. For all combinations of parameters, the expression of flavonoid biosynthesis enzymes is enriched within a single cluster.

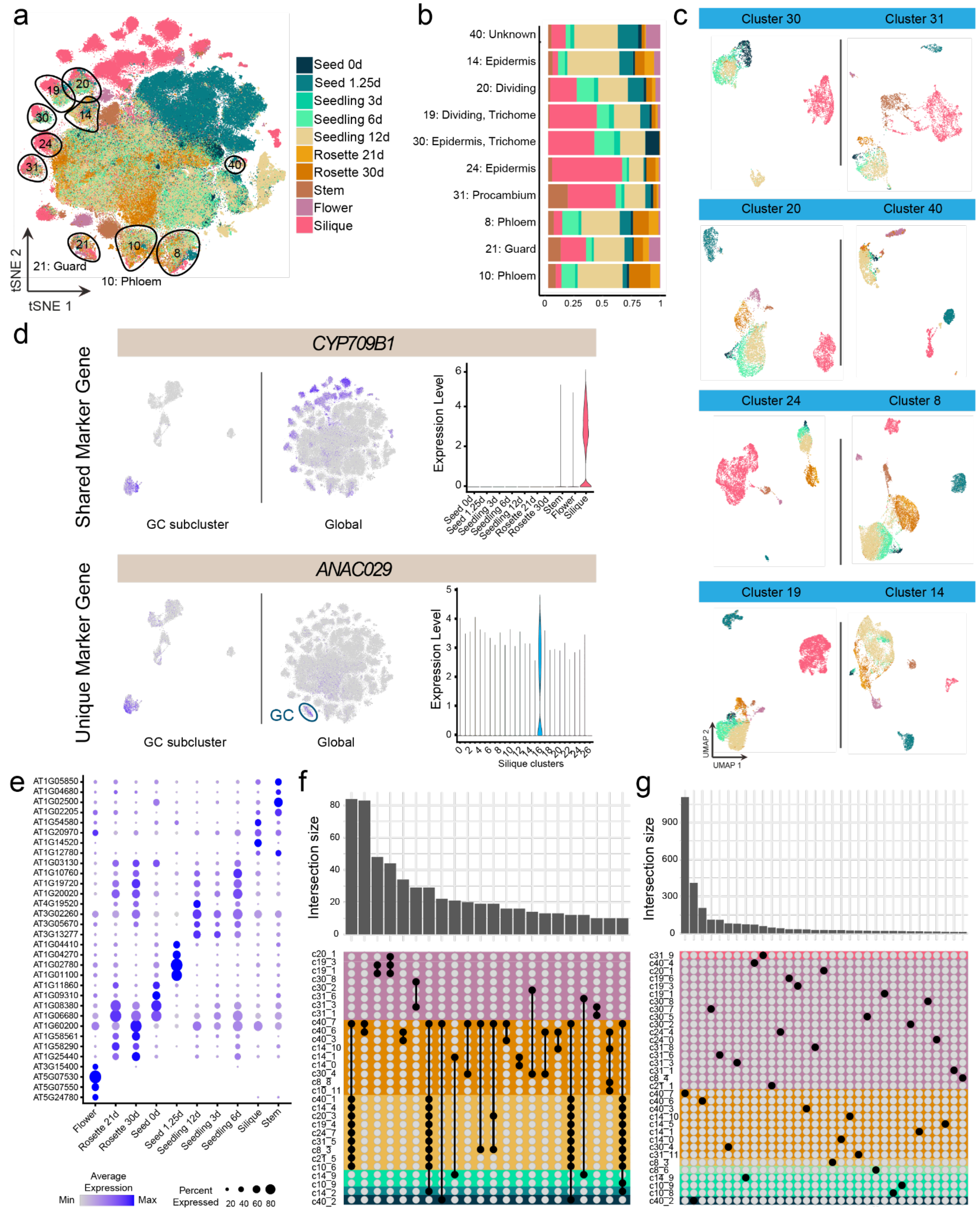

Supplementary Fig. 14. Cross-tissue cell type analysis of ten cell type populations
a, The ten cell populations included in the cross-cell type analysis are highlighted in the globally clustered tSNE..b, Proportion of cells per dataset for the ten clusters analyzed. c, Subclustering of the remaining eight clusters included in the ten-cluster analysis. Nuclei are colored by the dataset of origin. d, Expression of a

representative shared tissue (*CYP709B1* [*AT2G46960*]) and unique marker (*ANAC029* [*AT1G69490*]) in the stomatal lineage subclustering, global integrated dataset, and expression within each tissue dataset. e, Expression of representative tissue-level markers identified for each tissue. f, Quantification of identified markers shared between subcluster populations. Groups are named by cluster of origin and subcluster number. Tissue of origin is depicted by the colored bar. Subcluster intersections with greater than ten shared markers are shown. g, Quantification of subcluster markers uniquely identified as markers in only one subcluster population. Groups are named by cluster of origin and subcluster number. Tissue of origin is depicted by the colored bar. Subclusters with greater than ten uniquely identified marker genes are depicted.

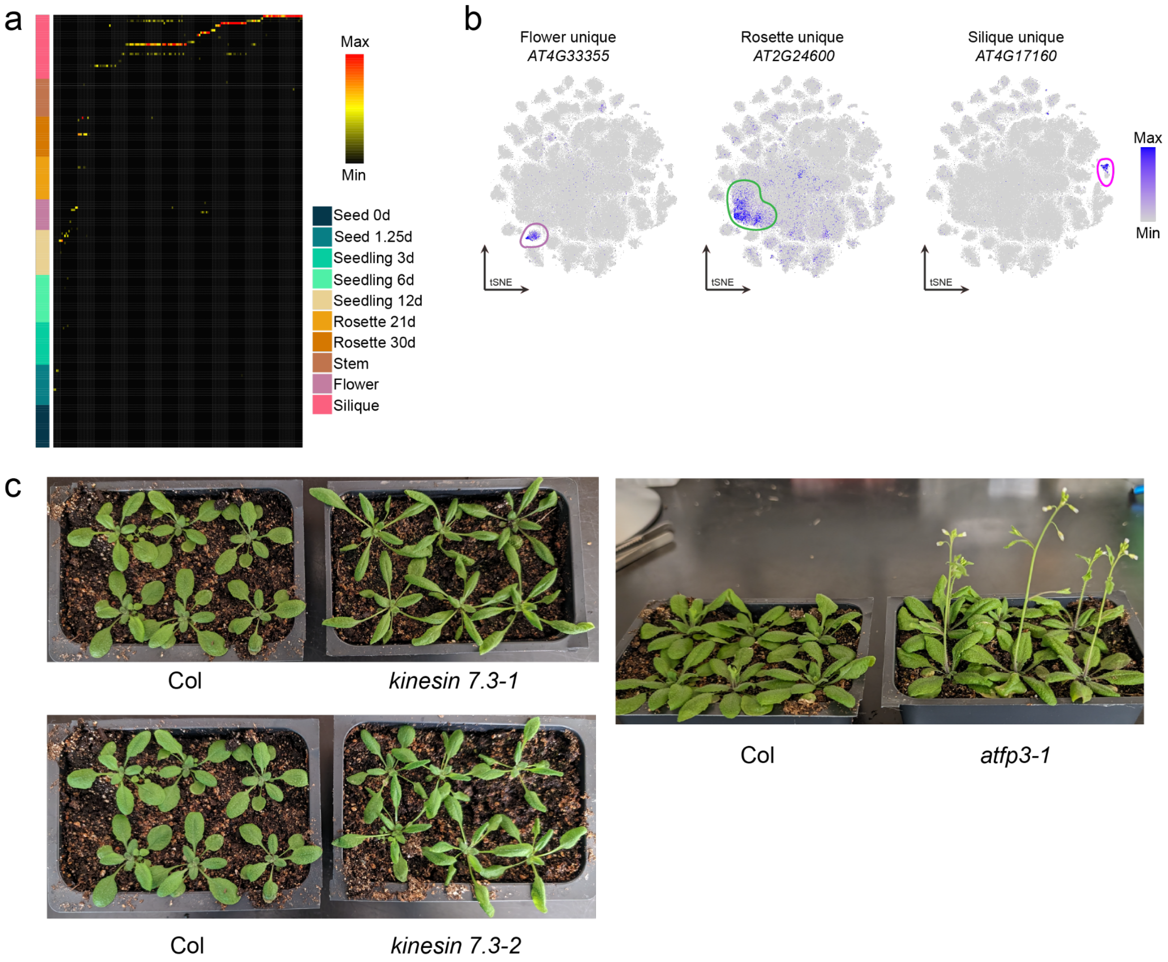

Supplementary Fig. 15. Investigation and functional validation of genes with cell type and developmental specificity
a, Heatmap showing expression of genes uniquely enriched within only one of 183 clusters. b, Expression of unique cluster markers from the flower (left), rosette (middle), and silique (right) datasets at the global clustering level. c, Images of T-DNA mutants with phenotypic defects in petiole length and leaf morphology (*kinesin 7.3* [*AT3G12020*], left) and early bolting (*atfp3* [*AT5G63530*], right).

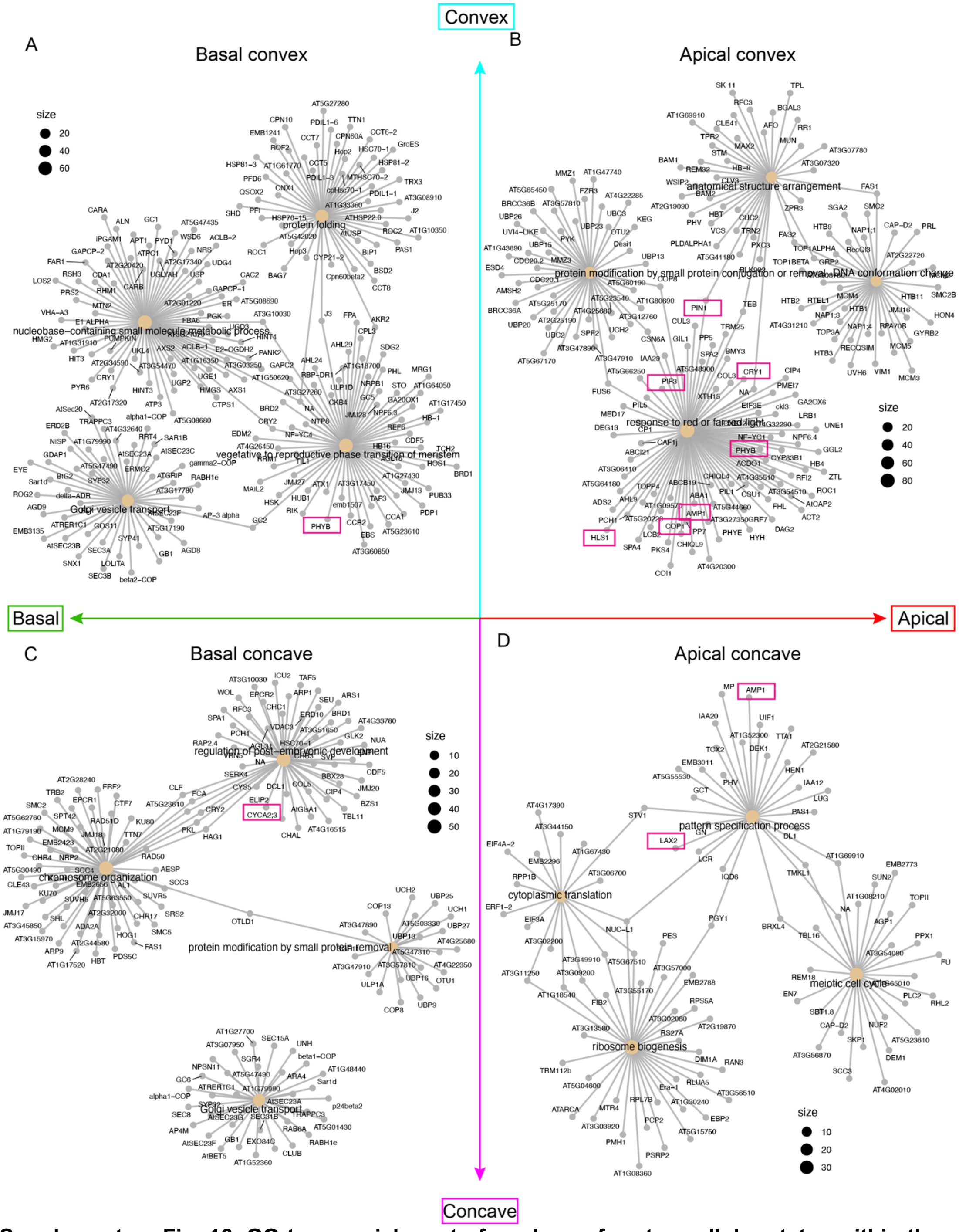

Supplementary Fig. 16. GO term enrichment of markers of cortex cellular states within the apical hook

a to d, The top four enriched GO terms and associated genes of marker identified within each combination of cellular states within the apical hook (Extended Data Fig. 10). Genes with known function in apical hook regulation are highlighted.

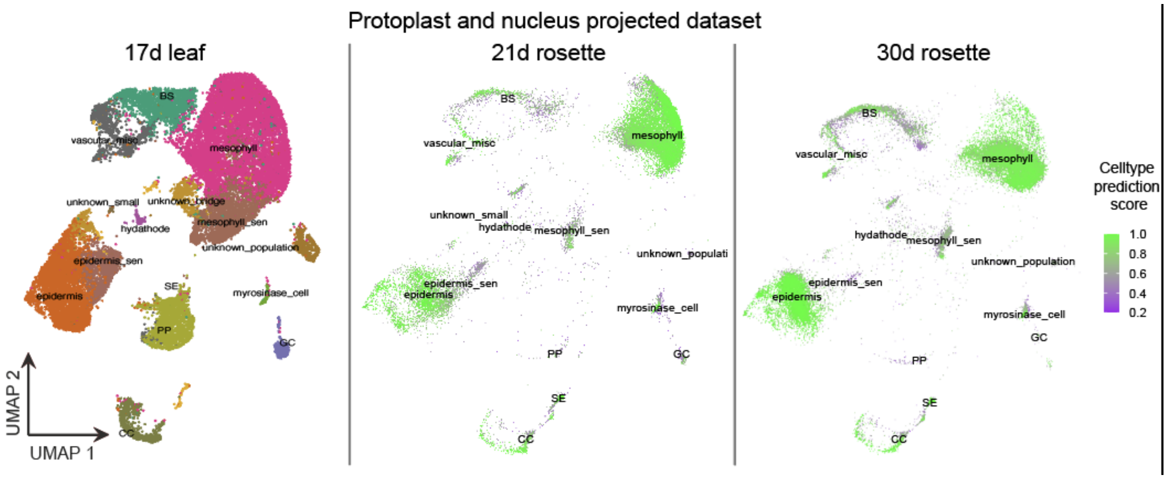

Supplementary Fig. 17 Integration of the rosette single-nuclei datasets with a protoplast dataset
Cell type prediction scores of the 21d- (middle) and 30d-old (right) rosette nuclei datasets when projected onto a leaf protoplast dataset (left; Procko et al., 2022).

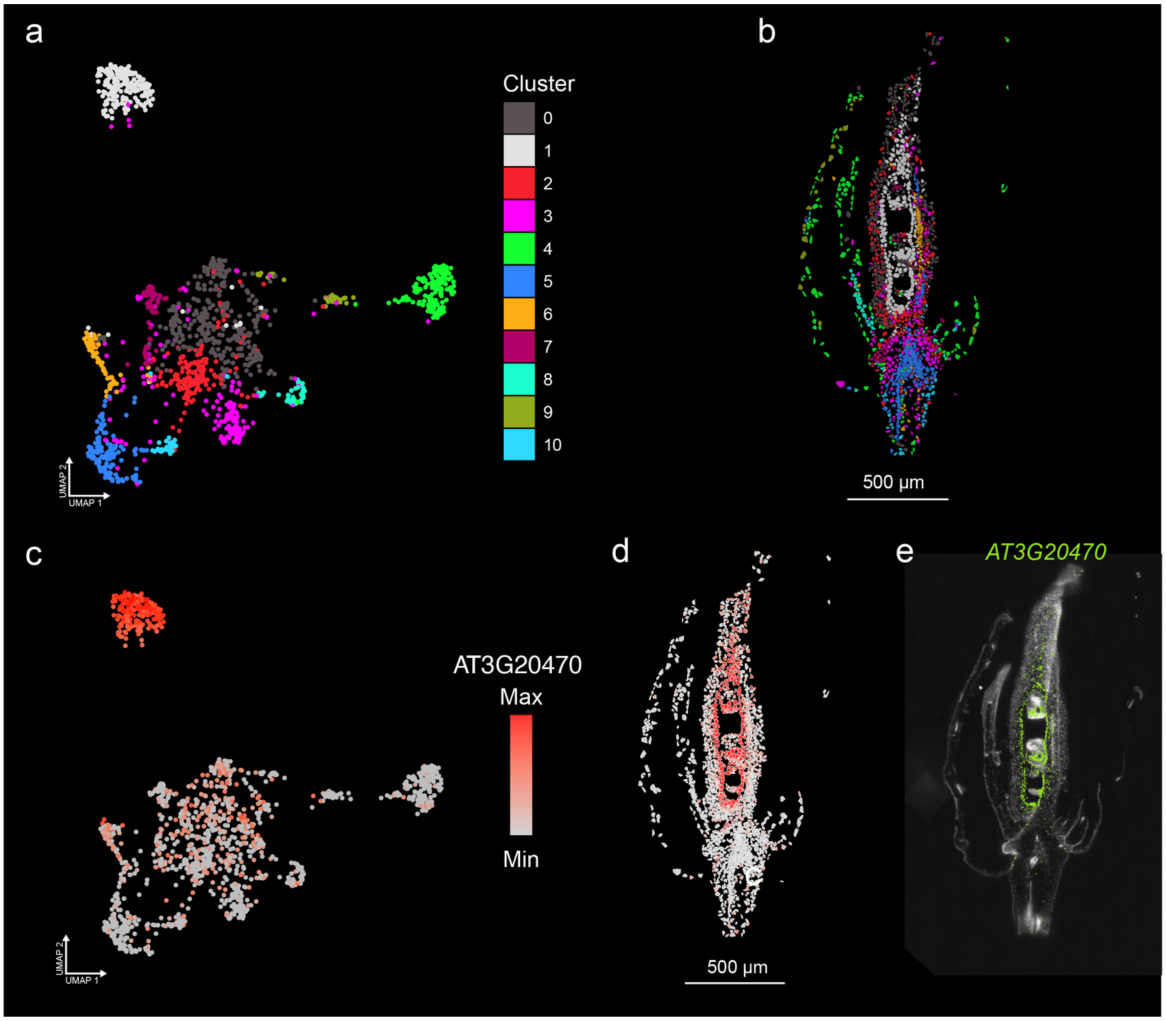

Supplementary Fig. 18 Spatial single-cell analysis of the longitudinal flower MERFISH dataset
a and b, *de novo* clustering of the segmented longitudinal flower dataset. Cells are colored according to cluster number and are displayed in (a) UMAP reduced dimensions and (b) spatial coordinates of cells. Scale bar = 500 µm. c and d, Single-cell expression of a novel cluster marker *AT3G20470* in (c) UMAP reduced dimensions and (d) spatial coordinates of cells. Scale bar = 500 µm. e, Spatial single-molecule detection of *AT3G20470*. DAPI signal is colored white.

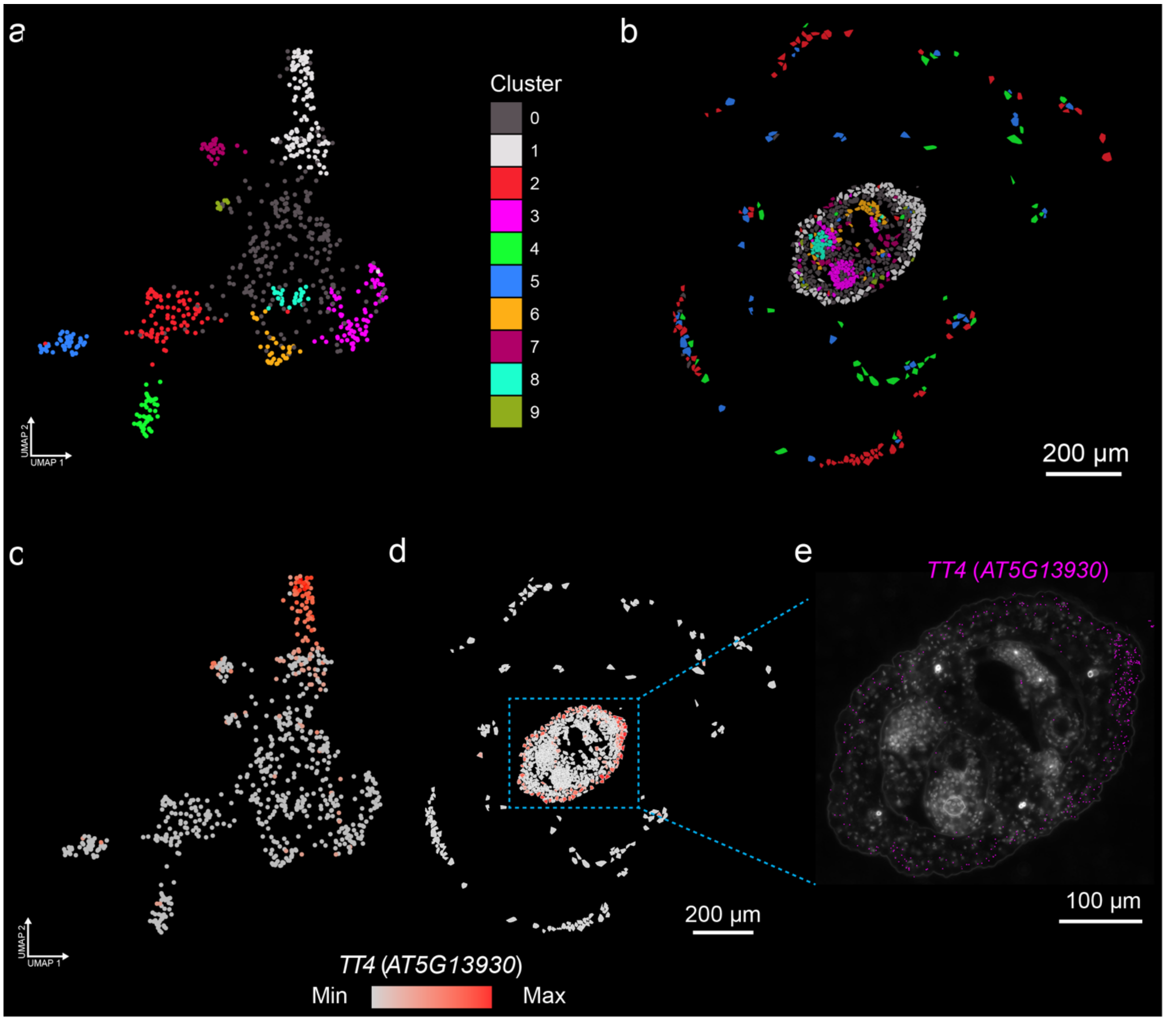

Supplementary Fig. 19 Spatial single-cell analysis of a MERFISH flower cross section dataset
a and b, *de novo* clustering of the segmented cross sectioned flower dataset. Cells are colored according to cluster number and are displayed in (a) UMAP reduced dimensions and (b) spatial coordinates of cells. c and d, Expression of a novel cluster marker *TT4* (*AT5G13930*) in (c) UMAP reduced dimensions and (d) spatial coordinates of cells. Scale bar = 200µm. e, Spatial single-molecule detection of *TT4* in the area depicted in (d) Scale bar = 100µm. DAPI signal is colored white.
